# Toward high-selectivity CO_2_ photoelectroreduction: mechanistic foundations, recent advances and challenges

**DOI:** 10.1039/d5sc02284c

**Published:** 2025-06-12

**Authors:** Guosheng Zhou, Zhenzhen Wang, Junjie Gong, Shijie Shen, Wenwu Zhong

**Affiliations:** a Zhejiang Key Laboratory for Island Green Energy and New Materials, Taizhou University Jiaojiang 318000 Zhejiang P. R. China shensj@tzc.edu.cn zhongww@tzc.edu.cn; b School of Chemistry and Chemical Engineering, Shaoxing University Shaoxing 312000 P. R. China

## Abstract

Photoelectrochemical carbon dioxide reduction reaction (PEC CO_2_RR) is a promising strategy for converting CO_2_ into high-value chemicals that contribute to carbon neutrality. However, CO_2_ reduction is often accompanied by various competitive reaction pathways in the actual reaction process, which may generate a variety of products. The selective regulation of different products not only directly affects the yield and separation cost of the target product, but also influences the energy efficiency and economic feasibility of the whole process. Improving product selectivity is essential for increasing product yield and understanding the reaction mechanism. This review systematically summarizes recent advances and challenges in achieving high selectivity in PEC CO_2_RR. First, the basic concept and principle of the PEC CO_2_RR are summarized. Next, the key factors affecting product selectivity are discussed, including catalyst design (catalyst type, modification, composition, and morphology), reaction conditions (applied voltage, light intensity and wavelength, reaction temperature, and electrolyte type) and reactor design (photoelectrode area, synergistic oxidation effect, geometric structure, and gas diffusion electrode). In addition, kinetic and thermodynamic aspects such as the CO_2_ adsorption model, band gap structure, and reaction free energy are also explored. Then, the research progress over the past five years on different products is described in detail, focusing on the current status and challenges in the study of C_1_ products and C_2_ products. Subsequently, the primary factors leading to the failure of PEC CO_2_RR are summarized, and various cooperative strategies are introduced to achieve long-term stability in product selectivity. Finally, the challenges and future directions for developing PEC CO_2_RR systems with enhanced selectivity are introduced. In particular, the importance of innovative catalyst design, reaction stability, reaction environment optimization, advanced equipment structure and reaction mechanism analysis for promoting PEC CO_2_RR in industrial applications is emphasized.

## Abstract 2

This review systematically explores strategies to enhance product selectivity in PEC CO_2_RR, and further analyzes recent advances in C_1_/C_2_ product synthesis, challenges in selectivity control, and future directions.

## Graphical abstract

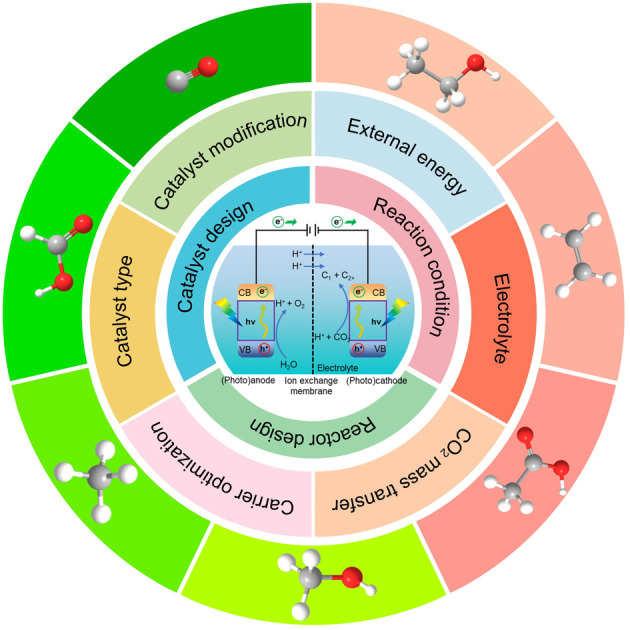

## 1 Introduction

**Fig. 1:**
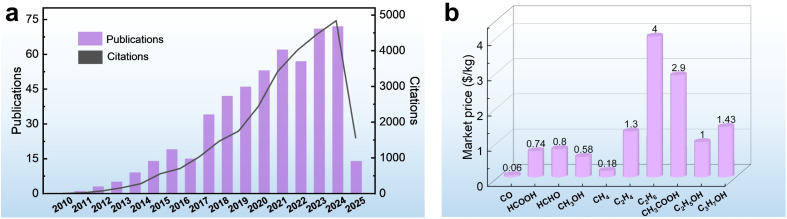
(a) The number of publications and citations related to PEC CO_2_RR, according to the data collected from *Web of Science* (the key word is “Photoelectrocatalytic CO_2_ Reduction”, and the data are collected up to May 2025). (b) The market prices of different products.^14^

With the rapid advancement of industrialization, the excessive consumption of fossil fuels has led to a dramatic increase in carbon dioxide (CO_2_) emissions, posing a serious threat to the stability of the global climate system.^1–5^ As one of the major greenhouse gases, the concentration of CO_2_ in the atmosphere has risen from 280 ppm before the Industrial Revolution to an annual average of 419 ppm in 2022.^6^ To address these challenges, scientists are working to develop efficient and environmentally friendly technologies to capture and convert CO_2_ to achieve carbon neutrality. Among these technological pathways, the photoelectrochemical CO_2_ reduction reaction (PEC CO_2_RR) is regarded as one of the ideal solutions for realizing “artificial photosynthesis” due to its ability to synergistically drive the conversion of CO_2_ into high-value-added carbon-based fuels by directly utilizing both solar energy and electrical energy.^7–10^ The technology is expected to not only reduce the concentration of CO_2_ in the atmosphere, but also convert renewable energy sources into chemical energy storage, providing a dual solution to address the energy crisis and the imbalance of the carbon cycle.^11,12^ Since 2009, the number of publications and citations on PEC CO_2_RR has increased year by year, indicating the progress in this field (Fig. 1a). However, the current technology is still in its infancy and faces many challenges, especially the problem of product selectivity control.

The CO_2_RR usually involve multiple competing reduction pathways that lead to the formation of different products such as carbon monoxide (CO), formic acid (HCOOH), methanol (CH_3_OH), methane (CH_4_), acetic acid (CH_3_COOH), ethylene (C_2_H_4_), ethanol (C_2_H_5_OH), *etc.*^13^ There are significant differences in the market value of these products (Fig. 1b).^14^ For example, the price of HCOOH is 12.3 times that of CO. The differing economic values of the products are prompting researchers to focus on how to modulate the reaction pathway through catalysts to achieve highly selective product generation. From the perspective of industrial applications, a single product not only reduces the complexity of subsequent separation and purification, but also effectively reduces the cost of post-processing, making the process of product extraction and conversion more economically viable.^15,16^ For example, in CO_2_ reduction systems, the products exist as mixed gases (*e.g.*, CO/CH_4_) or liquid mixtures (*e.g.*, CH_3_OH/C_2_H_5_OH); additional energy-intensive processes such as distillation, adsorption, or membrane separation are required, resulting in a decrease in efficiency and economy. Therefore, achieving highly selective generation of target products is the core challenge for improving the efficiency of CO_2_ resource utilization, both in terms of product value and process feasibility.

The multiple barriers to selective modulation are due to the high stability of the CO_2_ molecule and the complexity of the multi-electron transfer process. The linear symmetric structure of CO_2_ and the high bond energy of the C=O bond (approximately 750 kJ mol^−1^) necessitate overcoming significant thermodynamic barriers for its activation.^7,17,18^ In the photoelectrocatalytic process, CO_2_ first forms key intermediates (*e.g.*, *CO_2_, *COOH, or *OCHO) *via* proton-coupled electron transfer (PCET), and then undergoes multi-step reduction and protonation reactions to generate the final product.^19^ In this process, the energy barriers of different reaction pathways differ by only tens to hundreds of millielectronvolts, and small energy fluctuations can lead to the diversion of reaction pathways.^20^ For example, the generation of C_2+_ products requires the adsorption of at least two CO intermediates, their migration and C–C coupling, followed by successive hydrogenation steps. The activation energy of each reaction step can be a rate-determining step. In addition, the HER is an important competing reaction that competes with the generation of the target product for protons and electrons, further increasing the difficulty of regulating product selectivity.^21^ For example, on the surface of copper-based catalysts, the Faraday efficiency (FE) of C_2_H_4_ can reach 61% when the applied potential is −0.74 V *vs.* RHE, but at the same time the hydrogen evolution reaction accounts for nearly 30%.^22^ The prevalence of such competing reactions makes selective modulation a delicate balance between thermodynamic driving force and kinetics.

In recent years, researchers have made remarkable progress in material design, reactor optimization and mechanistic investigation related to the regulation strategy of product selectivity. First of all, in the design of photocatalysts, it is necessary to accurately construct a suitable catalytic system based on the chemical properties of the target product.^23^ Through band engineering, the semiconductor bandgap structure can be adjusted to match the solar spectrum, and the electronic density distribution can be optimized by combining defect engineering, elemental doping, and surface modification.^24,25^ Furthermore, the design of heterojunction interfaces promotes the directional migration of photogenerated carriers, while the use of co-catalysts can enhance the stabilization of specific intermediates through localized surface plasmon resonance effects or selective adsorption properties.^26–28^ In addition, nanostructural morphology modification (*e.g.*, porous frameworks, nanoflowers and core–shells) can increase active site density and improve reactant mass transfer efficiency.^29–31^ Secondly, in the optimization of reaction conditions, temperature, pressure, light intensity, light wavelength, and electrolyte type and parameters are systematically regulated to enhance the desired reaction pathways and inhibit the side reaction pathways by balancing the thermodynamic and kinetic parameters of the reaction, such as adjusting the rate of proton supply or decreasing the competitive activity of hydrogen precipitation to enhance the selectivity of the target products.^32^ Reaction device design innovation can be achieved by optimizing the photoelectrode structure, mass transfer channels and reactor configuration, anodic oxidation reaction design, *etc.*, to achieve a synergistic enhancement of light energy absorption efficiency and reaction kinetics; it can also be combined with a visible light-responsive photocathode microbial system to achieve the effective immobilization of CO_2_ and highly selective generation of reduction products.^32^ A deep understanding of the reaction mechanism is also indispensable for guiding the experimental design. By combining *in situ* characterization techniques to track the dynamic evolution of intermediates in real time and leveraging quantum chemical calculations to analyse the electronic structure of active sites and the energy barrier distribution of reaction pathways, the microscopic mechanisms of selective control are revealed at the atomic level. This provides theoretical guidance for catalyst design and reaction condition optimization, driving the transition of PEC CO_2_RR from empirical trial-and-error to rational design.

**Fig. 2:**
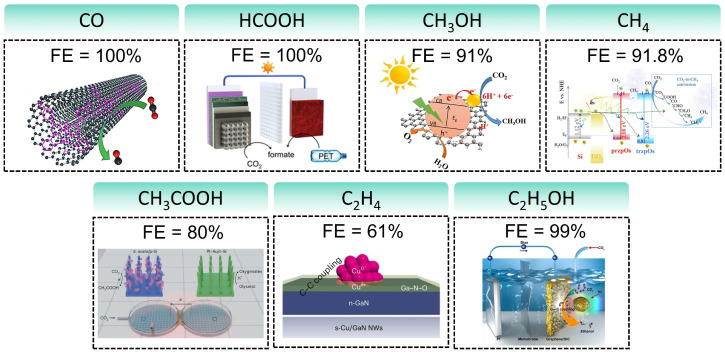
The highest FEs of different PEC CO_2_RR products in the past five years. Reproduced with permission from ref. 22, 107, 114, 205, 222, 230, and 254. Copyright 2022, Wiley-VCH GmbH. Copyright 2024, The Royal Society of Chemistry. Copyright 2021, Elsevier B.V. All rights reserved. Copyright 2022, Elsevier B.V. All rights reserved. Copyright 2024, Springer Nature. Copyright 2024, Springer Nature. Copyright 2023, Wiley-VCH GmbH.

Therefore, this review aims to systematically elucidate the factors influencing the product selectivity in PEC CO_2_RR and summarize the recent research progress on typical products, with a view to providing references and insights for future research. First, this review introduces the fundamental concepts of PEC CO_2_RR and the factors affecting product selectivity, including catalyst design, reaction condition design, and device structure design. The product selectivity of PEC CO_2_RR is also explored from both kinetic and thermodynamic perspectives. Subsequently, this review provides a detailed description of the research progress over the past five years on different products, with a focus on C_1_ products (such as CO, HCOOH, CH_3_OH, and CH_4_) and C_2+_ products (such as C_2_H_4_, C_2_H_5_OH, and CH_3_COOH), highlighting the current research status and challenges. Based on the above progress, we summarized the representative studies of PEC CO_2_RR products with the highest FE, as shown in Fig. 2. Finally, this review provides an outlook on the future development directions of PEC CO_2_RR technology, emphasizes the importance of improving product selectivity and efficiency, and highlights the areas that need to be further explored. Through systematic sorting and summarization, this review aims to provide valuable references for researchers and promote continuous innovation and advancement in PEC CO_2_RR technology.

## 2 Basic principles of PEC CO_2_RR

### 2.1 Fundamentals of PEC CO_2_RR

#### 2.1.1 A brief introduction to PEC CO_2_RR

To better understand the PEC CO_2_RR process, it is necessary to understand the device structure and related basic concepts. First, the basic concepts and the four types of devices are introduced in detail. Then, the types and application scenarios of the electrode system and ion-exchange membranes are introduced. Finally, the key parameters related to the selectivity of the catalytic reaction are briefly described.

PEC CO_2_RR is a technology that combines light and electrical energy to drive the CO_2_ reduction reaction. It is based on the synergistic effect of photocatalysis and electrocatalysis. Photoelectrocatalysis generates photogenerated electrons and holes through the absorption of light energy by the photocatalyst. These photogenerated carriers are subsequently separated and transported through an external circuit to participate in the reduction and oxidation reactions, respectively. In the CO_2_ reduction process, photogenerated electrons are transported to the reduction reaction site, where they promote the reduction of CO_2_. The process utilizes light energy and electrical energy, improving the reaction efficiency and product selectivity. However, photocatalytic technology has significant drawbacks: firstly, the high carrier complexation rate and the tendency of electrons and holes to recombine before migrating to the catalyst surface, resulting in low energy utilization; secondly, the reaction kinetics are slow.^33^ CO_2_ molecules exhibit strong chemical inertness and require high energy for activation, while the reduction ability of photogenerated electrons is limited, making it difficult to drive multi-electron transfer reactions (such as generating C_2+_ products). In contrast, electrocatalytic technology drives the reaction through an externally applied electric field, directly reducing CO_2_ at the catalytic sites on the electrode surface. Theoretically, applying an electrocatalytic field can regulate the electron transfer rate and the reaction path orientation to produce specific products. However, its limitations are also prominent: firstly, CO_2_ reduction requires energy input to overcome high thermodynamic barriers and easily triggers the competitive HER, resulting in the reduction of FE. Secondly, CO_2_ reduction involves multi-electron and proton transfer processes, in which the difference in adsorption strength of different intermediates (such as *COOH, *CO, *etc.*) makes it difficult to control product selectivity. In addition, the electrode material is prone to corrosion or poisoning under long-term high voltage or acidic/alkaline conditions, affecting the sustainability of the reaction.^34,35^

**Fig. 3:**
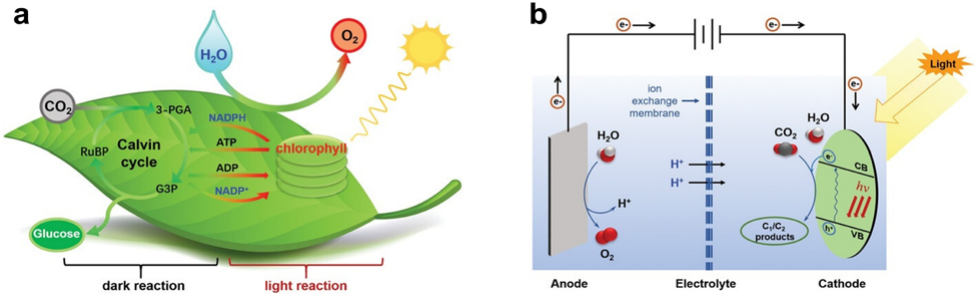
Schematics of (a) natural photosynthesis, and (b) PEC CO_2_RR. Reproduced with permission from ref. 37. Copyright 2022, Wiley-VCH GmbH.

PEC CO_2_RR technology combines the advantages of photocatalysis and electrocatalysis and overcomes their limitations. Firstly, photoelectrocatalysis utilizes the synergistic effect of light and electric energy, significantly improving the reaction efficiency and energy conversion efficiency. Secondly, by designing the structure and composition of the photocatalyst, the reaction pathway can be effectively regulated to achieve high selectivity for the target products.^36^ In addition, the utilization of solar energy as the driving energy source is sustainable. Similar to photosynthesis in nature, PEC CO_2_RR technology is regarded as an artificial simulation of photosynthesis (Fig. 3).^37^ In photosynthesis, photosynthetic system II in chloroplasts absorbs photons and excites electron transitions to produce high-energy electrons and holes. Electrons drive the oxidation of water through the transfer chain (generating O_2_ and H_2_O), while generating ATP and NADPH (energy carrier). In the Calvin cycle, the energy provided by NADPH and ATP fixes CO_2_ to glucose, a process catalyzed by enzymes. Semiconductor materials (*e.g.* TiO_2_ and perovskite) absorb photons to produce electron–hole pairs, and photogenerated electrons are injected into the conduction band while holes participate in oxidation reactions (*e.g.*, water decomposition). The electrocatalytic phase is similar to the Calvin cycle of carbon fixation. By regulating the reaction path through an external circuit, the external electric field drives the electrons to migrate to the catalytic sites, reducing CO_2_ to produce the target product. Compared with photosynthesis, photocatalysis has better controllability and flexibility: by designing the structure of the photocatalyst and tuning the reaction conditions, precise regulation of the reaction path and product can be achieved. In addition, photocatalytic technology can work in a wider spectral range, improving the utilization of light energy. Overall, the PEC CO_2_RR technology overcomes the limitations of traditional methods and shows both potential for efficiency and sustainability. By further optimizing the design and performance of the photocatalysts, photoelectrocatalysis is expected to achieve large-scale CO_2_ resource utilization in the future, providing an important solution to address global climate change and energy crisis.

**Fig. 4:**
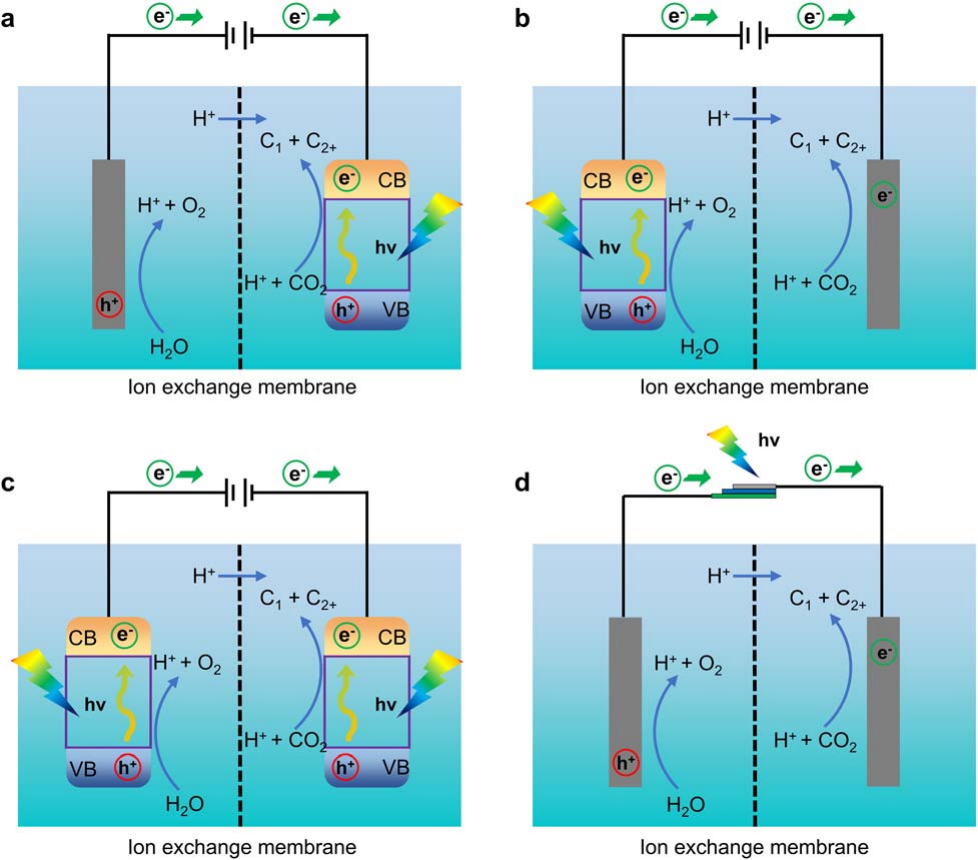
Device types of photocatalytic CO_2_ reduction systems. (a) The photocathode drive system, (b) the photoanode drive system, (c) the photoanode and photocathode co-drive system, and (d) the hybrid optical system combining the electrode with the photovoltaic cell.

The types of devices for PEC CO_2_RR systems can be categorized into four main types based on the combination of optical and electrical drives (Fig. 4): photocathode-driven systems, photoanode driven systems, photoanode and photocathode-*co*-driven systems, and hybrid photovoltaic systems that combine electrodes with photovoltaic cells.^38^ Each of these four device types has its own design characteristics and functionalities suited to different experimental needs and scenarios. A photocathode-driven system is a photocatalytic system based on a photocathode, which mainly reduces CO_2_ to high value-added chemicals or fuels. In this system, the photocathode is usually made of a p-type semiconductor material with photocatalytic capability, and requires the assistance of an external power source to provide additional energy to overcome the high activation energy of the reaction. The advantage of this system is that it can regulate the selectivity of the product by adjusting the applied potential. It is suitable for studying the performance of photocatalysts in reduction reactions. In contrast, the photoanode-driven system uses the photoanode to decompose water to produce oxygen, while achieving CO_2_ reduction reaction on the photocathode. This system does not require an external power source because the photogenerated charge generated by the photoanode drives the reaction itself. Photoanodes are usually made of n-type semiconductor materials. This device is characterized by efficient use of light energy, and can achieve CO_2_ reduction without external energy input, which is suitable for the research of self-driven photocatalytic systems. The photo-anode and photocathode co-driven systems combines a photo-anode and a photocathode to enable simultaneous oxidation and reduction reactions. In this system, the photoanode and photocathode are responsible for the oxidation of water and the reduction of CO_2_, respectively, while achieving internal charge balance through the charge transport network. The advantage of this system is that it can realize the efficient utilization of light energy, and can effectively improve the overall reaction efficiency by simultaneously conducting oxidation and reduction reactions. The co-driven system is suitable for studying the performance of photocatalysts in complex reaction systems and exploring their potential for practical applications. Hybrid light systems combining electrodes with photovoltaic cells are an innovative type of device. In this system, a conventional electrode is combined with a photovoltaic cell, and the photogenerated voltage generated by the photovoltaic cell is utilized to drive the reduction reaction of CO_2_. Control of the reaction pathway is achieved through the regulation of the external circuit. The advantage of this system is that it can combine the adjustment of photo-generated voltage with an external power supply to achieve the dual optimization of product selectivity and reaction efficiency. The hybrid light system is suitable for exploring the synergistic effect of photoelectrocatalysis with other energy technologies and for providing new ideas for the practical application of photoelectrocatalysis. Overall, these four device types show their respective advantages and features in the CO_2_ reduction reaction through different driving modes and electrode designs. Based on specific experimental requirements and application scenarios, choosing an appropriate device type can effectively enhance the efficiency and product selectivity of PEC CO_2_RR, thus providing important technical support for realizing the resourceful utilization of CO_2_ and sustainable development.

**Fig. 5:**
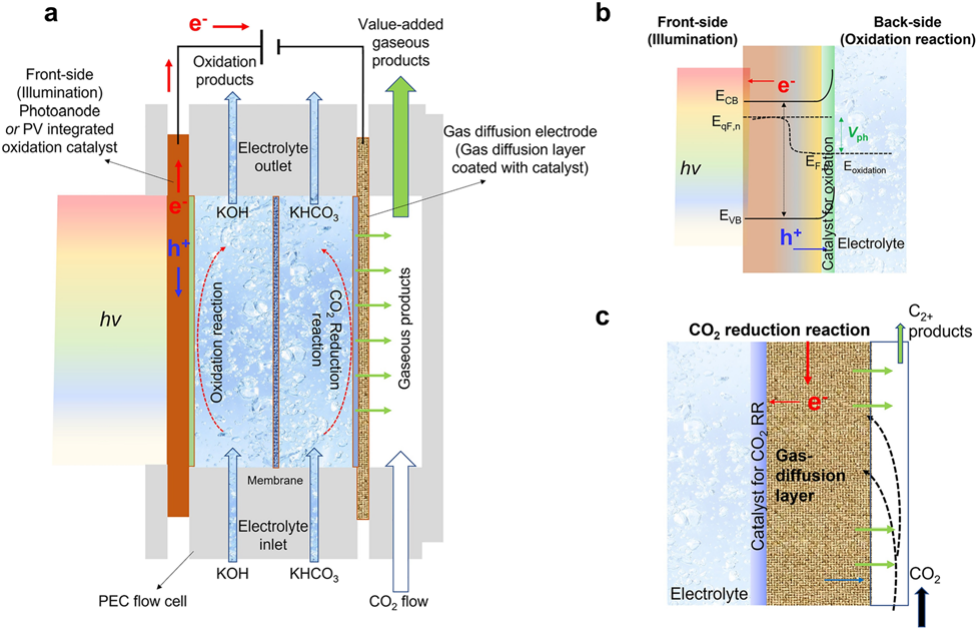
Schematic of a PEC flow cell for CO_2_RR using a GDE. (a) Schematic of PEC flow cell for CO_2_RR using a photoanode or a PV-integrated oxidation catalyst and a GDE. (b) Requirements of a photoanode/oxidation catalyst. (c) The GDE consists of a CO_2_RR catalyst coated on a porous gas-diffusion layer. Reproduced with permission from ref. 9. Copyright 2023, Elsevier B.V. All rights reserved.

Currently, a large number of studies employ H-type reactors for testing. This reactor features a separated, static design in which the anode and cathode chambers are divided by an ion exchange membrane, offering the advantages of a simple structure and low cost. However, mass transfer in this system relies primarily on natural diffusion or stirring, which limits mass transfer efficiency.^7^ Furthermore, the low solubility and diffusion rate of CO_2_ often result in low current density and faradaic efficiency. In recent research, the PEC flow cell reactor equipped with a gas diffusion electrode (GDE) has emerged as a highly promising PEC CO_2_RR reactor with significant potential for future development.^9^ Its design aims to integrate photoelectrocatalysis with fluid dynamics to achieve efficient gas–liquid–solid three-phase interfacial reactions, overcoming the limitations of CO_2_ solubility and enhancing CO_2_ mass transfer. As shown in Fig. 5, a typical PEC flow cell can be regarded as an evolution of the photoanode-driven system, in which the photoanode decomposes water to produce oxygen while CO_2_ reduction occurs at the photocathode.^9,39^ Unlike conventional setups, the flow cell reactor operates in a continuous flow regime, integrating gas diffusion electrodes and microchannel designs. By continuously supplying CO_2_ and removing products *via* forced flow, it significantly enhances mass transfer efficiency and reaction rates, achieving current densities ranging from tens to hundreds of mA cm^−2^, high CO_2_ utilization, and high selectivity for C_2+_ products, thereby demonstrating excellent scalability and industrial potential. The key lies in depositing catalyst particles onto a porous gas diffusion layer composed of carbon particles or nanofibers and a carbon paper substrate to fabricate the gas diffusion electrode. This electrode effectively transports CO_2_ gas while isolating the electrolyte, forming an efficient gas–liquid–solid three-phase reaction interface that enhances CO_2_ mass transfer, enables direct power delivery to the electrode, and increases current density (Fig. 5c). Additionally, the compact design reduces the distance between the two electrodes, thereby minimizing the system's internal resistance. However, the integration of multiple components, the stability of the gas diffusion electrode, and the collection of products increase the complexity of system design, maintenance, and operation.

Three-electrode and two-electrode systems are often used in the experimental design of PEC CO_2_RR. These two systems are suitable for different experimental conditions and targets. The three-electrode system consists of a working electrode (WE), a counter electrode (CE) and a reference electrode (RE). The WE is usually a conductive substrate loaded with a photocatalyst, such as FTO glass or Ti sheet. The CE uses a material with great conductivity, such as platinum, graphite or molybdenum. The RE is used to measure the potential and the commonly used materials include Ag/AgCl, Hg/Hg_2_SO_4_, and Hg/HgO. The three-electrode system is usually used in conjunction with a potentiostat to perform experiments at a constant potential. The reaction path is precisely controlled by adjusting the potential to achieve the selective control of the product. It is suitable for the situations where electrochemical measurements are required, such as current–voltage curves and electrochemical impedance spectroscopy analysis. The advantage of the three-electrode system is that it can accurately control the potential to study the electrochemical behavior of the photocatalyst. However, it is also more complex, requiring additional reference electrodes, and its long-term stability may affect the experimental results. In contrast, the two-electrode system consists of a photoanode or photocathode and a counter electrode. The photoanode is responsible for generating and transporting photogenerated electrons while the photocathode is responsible for generating and transporting photogenerated holes. Platinum or graphite is usually used as the counter electrode material. The two-electrode system is commonly used in photovoltaic devices or bias devices; the former directly uses the photogenerated voltage to drive the reaction, while the latter controls the reaction path by applying a bias voltage through an external power supply. The advantage of the two-electrode system is that the device is simple and suitable for studying the performance of the photocatalyst without external potential regulation, but its potential cannot be accurately controlled, which may affect the product selectivity.

In the PEC CO_2_RR device, the ion exchange membrane (IEM) plays a role in isolating the reaction solution and maintaining the charge balance.^40,41^ Different types of ion exchange membranes are suitable for different electrolytes. They are broadly classified into cation exchange membranes (CEMs), and anion exchange membranes (AEMs). CEMs allow cations to pass through and are suitable for acidic or neutral electrolytes, such as H_2_SO_4_, KHCO_3_, Na_2_SO_4_ or NaHCO_3_ electrolyte.^42–45^ AEMs allow anions to pass through and are suitable for alkaline or neutral electrolytes such as KOH, NaOH or KHCO_3_ electrolyte. In addition, there are solid electrolyte membranes made of metal oxides or inorganic materials, which are suitable for experimental conditions with high temperature or high stability requirements. The reasonable selection of electrolyte and ion exchange membrane types can effectively improve the efficiency and product selectivity of CO_2_ reduction, and provide important support for the efficient utilization of CO_2_.

The four basic parameters in the PEC CO_2_RR are as follows: photoelectrode materials, catalysts, electrolytes and external power sources. They play different roles in the reaction and have a close relationship with each other, determining the efficiency, selectivity and stability of the reaction. Photoelectrode materials are the core elements of photoelectrocatalytic reactions. They are usually made of semiconductor materials and are responsible for absorbing light energy and generating photogenerated electrons and holes. These photogenerated carriers need to be transported to the catalyst site through the surface of the material, where they then participate in the chemical reaction. The choice of photoelectrode material is very important, because it not only determines the absorption range and conversion efficiency of light energy, but also affects the separation and transmission ability of photogenerated carriers. Common photoelectrode materials include TiO_2_, WO_3_, BiVO_4_, Si, Fe_2_O_3_ and ZnO, which are widely used due to their excellent light absorption, stability and conductivity.^46–48^ Catalysts play indispensable roles in photoelectrocatalytic reactions, mainly by providing active catalytic sites and reducing the activation energy of the reaction to accelerate the reduction process of CO_2_. Catalysts can significantly improve the reaction rate and regulate the type of products, which is of great significance for the generation of highly selective products. The choice of catalysts depends on the reaction conditions and the expected products.^49–51^ In addition, the stability and durability of the catalyst are also the key factors affecting the long-term performance of the reaction. As a medium for ion transport, the electrolyte not only provides a suitable chemical environment for the reaction, but also maintains the charge balance of the entire system.^52^ The selection of electrolyte has an important influence on the reaction process, because it may participate in the intermediate steps of the reaction and affect the selectivity of the product. Therefore, depending on the different target products, the reasonable selection of electrolyte type and concentration is important for optimizing reaction performance. The role of external power supply in the photoelectrocatalytic system is mainly to adjust the potential of the system to ensure that the reaction is carried out under ideal conditions. By applying an appropriate voltage, the energy barrier of the reaction can be overcome, and the separation and transport of photogenerated carriers can be promoted, thereby improving the reaction efficiency. In addition, the external power supply can also be used to adjust the working mode of the device, such as switching between the self-driving mode under illumination conditions and the auxiliary driving mode under bias conditions. Proper setting of external power supply parameters, such as applied voltage and current, is essential to optimize reaction performance and achieve highly selective product generation. In summary, the performance and product selectivity of the PEC CO_2_RR are influenced by the photoelectrode materials, catalysts, electrolytes and external power sources. By optimizing the performance of each parameter and coordinating their interactions, the reaction efficiency and product selectivity can be significantly improved, laying a solid foundation for realizing the efficient utilization of CO_2_ as a resource.

#### 2.1.2 Product detection methods and the product selectivity calculation formula

The products of PEC CO_2_RR include various gaseous, liquid, and solid substances, and their types depend on the composition of the photocatalyst, reaction conditions, and device design. To accurately detect these products, it is usually necessary to rely on a variety of devices, depending on the category and nature of the products. The detection of gaseous products mainly relies on gas chromatography (GC) and mass spectrometry (MS). These devices can separate and quantitatively analyze gases such as methane CH_4_, CO, C_2_H_4_ and H_2_. Gas chromatography separates different gas components through a chromatography column and is combined with a flame ionization detector (FID) or thermal conductivity detector (TCD) for quantitative analysis, while mass spectrometry further improves the accuracy and sensitivity of product identification through ionization and mass analysis. The detection of liquid products mainly relies on ion chromatography (IC), high-performance liquid chromatography (HPLC), and nuclear magnetic resonance (NMR). These techniques can detect and analyze liquid products such as HCOOH, ethanol C_2_H_5_OH and CH_3_COOH. For example, IC detects ionic components (HCOO^−^ and CH_3_COO^−^) in liquid phase products and is combined with a conductivity detector or UV-vis detector for quantitative analysis; HPLC separates the components of the liquid products and is combined with a UV-vis detector or fluorescence detector for quantitative analysis; while NMR can provide information on the molecular structure of the products by analyzing changes in the chemical environment. A combination of multiple devices may be required, such as gas chromatography-mass spectrometry (GC-MS) for simultaneously separating and analyzing gaseous products. In conclusion, the detection of the products of PEC CO_2_RR requires the selection of appropriate devices and setups according to the types and characteristics of different products to achieve accurate qualitative and quantitative analysis of the products.

The product selectivity, FE and product photocurrent density in PEC CO_2_ reduction are the key parameters for evaluating the reaction performance.^39^ Their definitions and formulae are as follows:

The product selectivity (PS) refers to the ratio of the yield of a specific reduction product (A) to that of all possible products. Usually, in some studies, only a portion of reduction products is detected to calculate the relative selectivity. Its calculation formula is shown in eqn (1):1
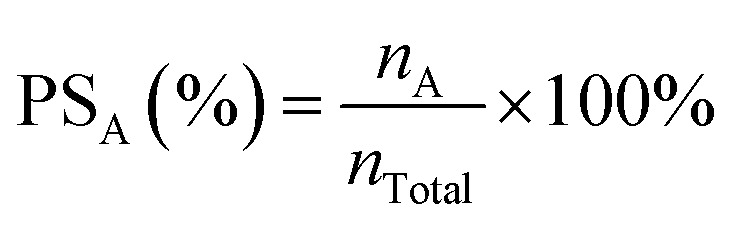
where PS_A_ is the product selectivity of A, the *n*_A_ (mol) is the molar mass of A, and *n*_Total_ (mol) is the molar mass of all reduction products.

FE is used to measure how much electrical charge is used to drive the CO_2_ reduction reaction. Specifically, FE represents the ratio of the number of electrons used to generate a specific reduction product to the total number of electrons involved in the electrochemical reaction. FE is an important indicator for measuring the selectivity and energy efficiency of the reaction. In the PEC CO_2_RR, a higher FE means that more electrical energy is used for the generation of the desired reduction product rather than being consumed by other side reactions (such as water decomposition). An ideal PEC CO_2_RR system should have a high FE, especially in the case of highly selective generation of the target product. Its calculation formula is shown in eqn (2):2
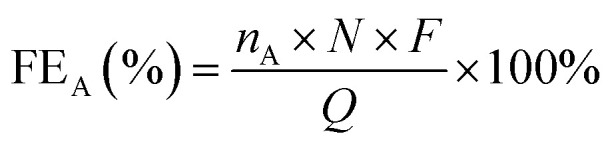
where FE_A_ is the FE of A, *n*_A_ (mol) is the molar mass of A, *N* is the number of electrons required to reduce CO_2_ to produce an A molecule, *F* is the Faraday constant (*F* = 96 485 C mol^−1^) and *Q* (C) is the total amount of charge.

The photocurrent density of the product (*j*_A_) refers to the current density of the product generated on the electrode per unit area. It indicates the current required per unit area on the catalyst surface to generate the target product under a specific voltage. A high photocurrent density usually means that the catalyst can more effectively convert light energy into electrical energy and drive the CO_2_ reduction reaction under given light conditions, thereby increasing the generation rate of the specific product. Its calculation formula is shown in eqn (3):3
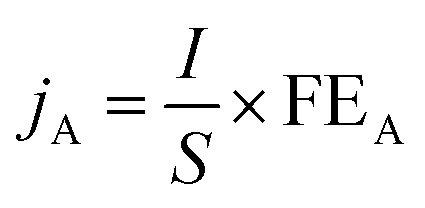
where *j*_A_ (mA cm^−2^) is the photocurrent density of A, *I* (mA) is the current intensity during the test, and *S* (cm^2^) is the effective area of the working electrode.

### 2.2 Factors affecting product selectivity

**Fig. 6:**
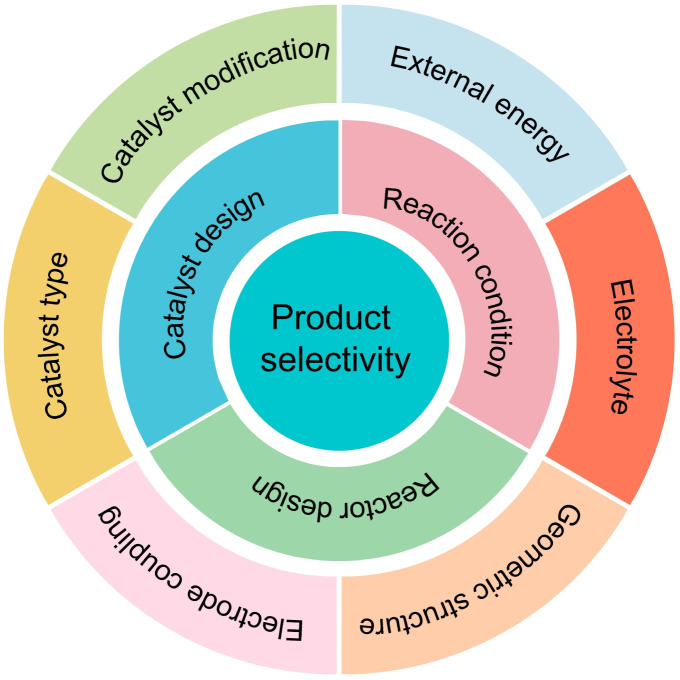
The factors affecting product selectivity: catalyst design, reaction conditions, and reactor design.

There are many parameters that affect the selectivity of products in the PEC CO_2_RR. In this review, we divide them into three aspects: catalyst design, reaction conditions, and reactor design, as shown in Fig. 6.

#### 2.2.1 Catalyst design

The effect of catalyst design on the selectivity of PEC CO_2_RR products is a complex and multi-level problem. Different catalyst types, modification strategies, composite structures and morphology control can affect the reaction path and the selectivity of the final product.

**Fig. 7:**
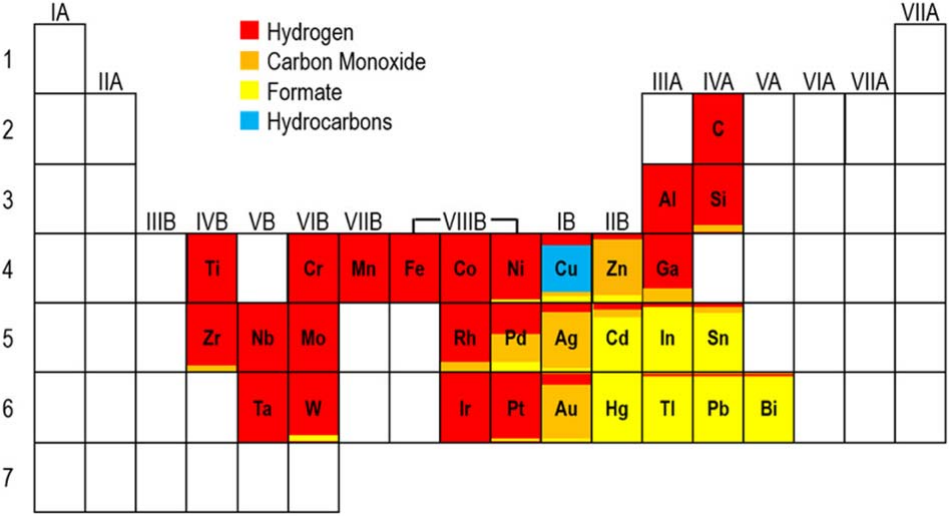
The main reduction products formed on metal and carbon electrodes in a CO_2_-saturated aqueous electrolyte, as represented across the periodic table. Reproduced with permission from ref. 24. Copyright 2015, American Chemical Society.

The choice of catalyst type is one of the important factors determining the selectivity of CO_2_ reduction products. Different metal catalysts exhibit varying selectivity in PEC CO_2_RR, mainly due to their unique electronic structures and active sites.^29,51,53–55^ White *et al.*^56^ summarized the product selectivity for CO_2_ reduction of typical elemental electrocatalysts that are usually attached to semiconductors as cocatalysts (Fig. 7). Precious metals such as Au and Ag are ideal for producing CO due to their moderate adsorption of CO intermediates.^26^ By providing a suitable d-band center, they can effectively stabilize CO intermediates and reduce their generation energy barrier.^21^ In addition, the weak adsorption capacity of these metals for hydrogen intermediates inhibits the HER, improving the selectivity for CO. In contrast, Cu-based catalysts are more suitable for the generation of multi-carbon products, such as (C_2_H_4_ and C_2_H_5_OH) because Cu-based catalysts can promote C–C coupling reaction, which is a key step in the formation of C_2+_ products.^57^ Bi, Sn, In, *etc.* are promising materials for the generation of HCOOH.^58^ Their electronic structure exhibit a moderate binding energy with the key intermediate *OCHO for the reduction of CO_2_ to formic acid, which not only promotes the stability of the intermediate, but also avoids the difficulty of desorption caused by excessive adsorption, thereby reducing the reaction energy barrier. Other transition metals such as Fe, Co, Rh, *etc.* tend to produce a large number of hydrogen by-products during the reaction process due to their low HER overpotential.^59^

The modification strategy of the catalyst greatly affects the selectivity of CO_2_ reduction products. Modification methods such as doping, vacancy, alloying, and crystal plane control can significantly change the electronic structure and surface properties of the catalyst, thereby regulating the adsorption and reaction path of the intermediate.^54,58,60–62^ For example, doping metal or non-metal elements can introduce new active sites or adjust the electron density of the original sites to improve the selectivity of specific products. When CO_2_ is reduced using 6 wt% Re-doped CuO/TiO_2_-NTs, the main products are methanol and ethanol. With a lower Re doping level of 1 wt%, methanol becomes the predominant product. In the absence of Re doping, formaldehyde is the primary product.^63^ In alloying, Bi-based materials can enhance the electronic structure of the Bi surface by introducing elements such as In or Sn, thus promoting the reduction of CO_2_ to HCOOH. In addition, crystal surface control also has an important effect on product selectivity. The exposure of different crystal faces can change the surface electronic structure and the active sites of the catalyst, which can selectively enhance the adsorption and conversion of some intermediates.^64^ For example, the (102) crystal plane of CuSe@BiOI heterojunctions can significantly improve the reduction selectivity of CO_2_ to HCOOH, while the (110) crystal plane of Cu_2_O is more conducive to the formation of ethanol.^65^ In summary, the electronic structure and surface characteristics of the catalyst can be regulated by modification, so as to achieve high selectivity for specific products.

The design of composite catalysts is another important strategy to improve the selectivity of CO_2_ reduction. The combination of different types of catalysts can achieve synergistic effects, optimizing electron transfer and enhancing the stability of intermediates.^66–68^ Metal particle loading can effectively improve the electronic structure of the catalyst to improve the photoelectric separation effect, and provide active sites for the reaction. The composite of Ag and Au nanoparticles with TiO_2_ can enhance the selectivity of CO through interfacial charge transfer, and the LSPR effect can further improve the photocurrent density.^26,69,70^ In addition, heterojunction structures are often constructed to enhance the separation and transport efficiency of photogenerated carriers.^71^ The design of composite catalysts is not limited to intermetallic composites, but also includes organic–inorganic hybrid materials, such as Cu_2_O composites with polyaniline (PANI), which significantly improve the selectivity of methanol by enhancing electron transfer and CO_2_ adsorption capacity.^72^ The microbial composite catalyst combines the high selectivity of microorganisms and the high efficiency of the catalyst to achieve the efficient conversion of CO_2_ to specific chemicals. For example, α-Fe_2_O_3_/g-C_3_N_4_ Z-scheme heterojunction photocathodes significantly increase acetate production by facilitating electron transfer between electrodes and microorganisms.^73^ The design of these composite structures makes full use of the synergy of different materials and significantly improves the selectivity and efficiency of CO_2_ reduction, providing a new idea for the development of efficient and stable catalysts.

The morphology control of catalysts has a significant effect on the selectivity of CO_2_ reduction products. Because of their high specific surface area and unique surface properties, nanostructured catalysts can provide more active sites, thus enhancing the adsorption and activation of CO_2_.^74,75^ For example, Cu_2_O nanocubes significantly improve the selectivity of CO_2_ reduction to formic acid by increasing the specific surface area and optimizing the charge transfer path. In addition, hollow structure catalysts such as CdS/VS-MoS_2_ heterojunctions significantly improve the selectivity of C_2_ products by increasing the probability of internal medium collision.^76^ The morphology control can also affect the product selectivity by changing the exposed crystal surface of the catalyst. For example, Cu_2_O nanowires can selectively generate multi-carbon products by exposing specific crystal faces. In addition, two-dimensional nanosheet structures such as Bi/Bi_2_O_2_CO_3_ significantly improve formic acid selectivity by providing more edge sites and active centers.^77^ Copper nanoflower catalysts exhibit excellent CO_2_RR activity due to their unique layered porous structure, which not only increases local pH but also reduces competitive hydrogen generation.^30^ These morphology control methods not only improve the activity of the catalyst, but also enhance the selectivity of specific products by optimizing the reaction path. The morphology control improves the activity and selectivity of the catalyst, providing a new possibility for the design of efficient photoelectrocatalytic systems.

The design of catalysts has a profound influence on the selectivity of CO_2_ reduction products. By selecting an appropriate metal catalyst, introducing doping or modification, constructing composite catalysts and adjusting catalyst morphology, the path of CO_2_ reduction can be effectively regulated, so as to achieve selective generation of specific products. These design strategies not only improve the reaction efficiency, but also provide theoretical guidance and experimental basis for the development of efficient and selective CO_2_ reduction catalysts. Future studies should continue to explore ways to optimize these design strategies to further improve the selectivity and stability of CO_2_ reduction, providing strong support for achieving sustainable carbon cycle and energy conversion.

#### 2.2.2 Reaction conditions

In the process of photoelectrocatalytic reduction of CO_2_, the selectivity of the product is mainly affected by the type of electrolyte, the applied voltage, the external light source (including light intensity and light wavelength), and so on.^78–83^ These factors directly affect the performance and reaction process of the photocatalyst by changing the reaction conditions and environment, and ultimately affect the type and proportion of the target product.

The applied voltage plays a key role in photoelectrocatalysis by regulating the separation and transport of photogenerated electrons and holes. By applying an appropriate voltage, the photo-generated carriers can be effectively separated, avoiding the recombination of electrons and holes, thereby enhancing the reaction efficiency.^84^ Meanwhile, regulating the applied voltage can change the reaction path and induce the reduction of CO_2_ to generate different target products. Ren *et al.*^85^ summarized and discussed the effect of varying the applied voltage on copper catalysts on the selectivity of CO_2_ reduction products. Specifically, the selectivity of the main products changed significantly across different voltage windows. When the applied potential is close to −0.7 V, the main products are CO and HCOO^−^, and the total FE can reach up to 75%, especially around −0.55 V. As the potential becomes negative and reaches between −0.8 V and −1.1 V, the production of C_2_H_4_ and C_2_H_5_OH increases significantly, especially for all oxide-derived copper catalysts. In addition, the process of generating syngas is very common. The ratio of CO and H_2_ was regulated by changing the applied voltage. The selective regulation of different CO_2_ reduction products can be achieved in different voltage windows by reasonably controlling the applied potential.

The light absorption and reaction efficiency of photocatalysts are directly affected by the intensity and wavelength of external light.^79,81,86,87^ An increase in light intensity usually increases the current density and increases the reaction rate. The role of light wavelength is also critical; different wavelengths of light are absorbed by the photocatalyst to different degrees, so selecting an appropriate light wavelength can maximize the light absorption efficiency of the photocatalyst, thereby improving the photogenerated carrier generation rate and reaction selectivity. Dong *et al.*^88^ found that the selectivity of the product did not change significantly with the increase in light intensity, that is, the FE of CO and H_2_ was almost the same under different light intensities, which meant that the selectivity of the reaction did not change with the change in light intensity. In addition, Sohn *et al.*^89^ studied the change in product selectivity under different lighting conditions. For ZGO/ZnO composites, the production of CO increased significantly under UVC light irradiation, while the production of CH_3_OH decreased. In contrast, under UVB light, the production of CH_3_OH increases significantly, becoming the most dominant product, followed by CO, while the production of CH_4_ and other C_2_ compounds decreases significantly.

The reaction temperature plays a crucial role in determining the reaction kinetics of PEC CO_2_RR.^8,90,91^ Light irradiation on the catalyst surface not only excites electron–hole pairs but also generates substantial heat through non-radiative relaxation, resulting in localized temperature increases that can range from several tens to over one hundred degrees Celsius.^92–94^ Such temperature rises can be achieved by incorporating carbon materials or utilizing the localized plasmonic effects of metal nanoparticles.^95^ On one hand, according to the Arrhenius equation, an increase in temperature generally accelerates the reaction rate, thereby enhancing the current density of CO_2_ reduction. For example, Cobb *et al.*^96^ employed a reaction setup with external heating to precisely control the system temperature. At 45 °C and 0.85 V *vs.* SHE, the current density increased by 60% compared to that at 20 °C. The current density for CO_2_ conversion to formate reached 0.47 ± 0.05 mA, and after 10 h, 42 ± 8 mmol of formate was produced. Additionally, the entropy effect at elevated temperatures leads to a reduction in Gibbs free energy, and the onset potential for water oxidation shifts slightly to lower values, thereby reducing the overall reaction energy barrier. On the other hand, temperature also affects the formation and stability of intermediates, thus altering the distribution of final products. According to the latest research results from Jing's team,^97^ compared to single photocatalysis, electrocatalysis, and thermal catalysis, thermally assisted photoelectrocatalysis achieves higher product yields and greater C_2_ product selectivity in carbon-based products, with the contribution of hot electrons to C_2_ product selectivity reaching 31.9%. This enhancement may be attributed to the reduced C–C coupling energy barrier between * = C=O and *CHO intermediates. However, increasing the reaction temperature is not always advantageous, as factors such as apparatus tolerance, catalyst stability, and other related issues must be considered. For instance, the enzyme formate dehydrogenase (FDh) catalyst tends to lose activity above 45 °C.^96^ Agbo's team investigated a flow cell setup and found that rapid heat dissipation of integrated components improved the solar-to-energy conversion efficiency by up to 10%.^81^

The type of electrolyte has a great influence on the type and selectivity of the products by affecting the stability and reaction path of the reaction intermediates.^87,98–103^ Different types of electrolytes provide different chemical environments, which may affect the properties of the active site on the photocatalyst surface.^104,105^ Liu *et al.*^82^ investigated the impact of the proton concentration of the electrolyte on the performance of CO_2_ reduction by testing three representative solvents, namely aprotic solvents with (methanol–acetonitrile) and without (acetonitrile) proton donors, and a protic solvent (methanol). The addition of proton donors not only increases the partial photocurrent and selectivity of CO, but also reduces photocorrosion. However, in methanol, the HER dominates the reduction of CO_2_, indicating that a high concentration of protons promotes the occurrence of HER. Compared with acetonitrile, the surface state concentration in methanol–acetonitrile significantly decreases, indicating that the presence of protons activates another reaction pathway (through stable intermediates), thereby accelerating charge transfer. The additives in the electrolyte significantly change the selectivity of CO_2_ reduction. For example, in an electrolyte containing 1-ethyl-3-methylimidazole tetrafluoroborate ([Emim]BF_4_), the selectivity of the Cu_2_O/TiO_2_ nanoarray for CO_2_ reduction to ethanol is greatly improved, and the FE reaches 82.7%.^103^ This is because the cation in the ionic liquid (IL) is enriched in the cathode region under external bias, which is conducive to capturing CO_2_, and forming a strong interaction with the catalyst surface, improving the separation efficiency and migration rate of photogenerated charges. In addition, the differing solubilities and adsorption capacities of electrolytes for CO_2_ reflect their mass transfer capabilities. Gao *et al.*^103^ utilized [Emim]BF_4_ ionic liquid as the electrolyte, which enhanced the CO_2_ solubility of the system and increased the local CO_2_ concentration at the catalyst surface. Furthermore, the [Emim]BF_4_ electrolyte activates the CO_2_ molecules and forms an [Emim^+^–CO_2_] complex, thereby lowering the energy barrier for the reduction reaction. Throughout the reaction process, [Emim]BF_4_ establishes an electron transfer channel between the catalyst and CO_2_, significantly enhancing the selectivity and activity for the conversion to C_2_H_5_OH.

By reasonably regulating the reaction conditions and environment such as the applied voltage, the intensity and wavelength of the external light, reaction temperature and the type of electrolyte, the product selectivity in the photoelectrocatalytic CO_2_ reduction process can be effectively controlled, so as to improve the generation proportion of the target product and enhance the reaction efficiency. This systematic regulation strategy not only helps to improve the performance of photoelectrocatalytic technology, but also provides a new idea for the efficient selective reduction of CO_2_.

#### 2.2.3 Reactor design

In the PEC CO_2_RR process, the design of devices plays a key role in the selectivity of products.^9,78,96,106^ The area and synergistic oxidation of the photoelectrode directly affect the absorption of light energy and the efficiency of charge transport, thus affecting the selectivity of the product. In addition, the design of the reactor also has an important influence on the transport of reactants, the exposure of the catalyst and the selectivity of the product.^70,107^ The design of the photoelectrode area needs to comprehensively consider the light absorption efficiency and charge transport capacity. An excessively large area may lead to uneven absorption of light energy or hinder charge transport, which in turn affects the selectivity of the product. Andrei *et al.*^30^ found that when the effective area of the catalyst was about 4 mm^2^, the FE of C_2_ hydrocarbons reached 9.8%, which is much higher than that of larger catalysts. This is because the smaller catalyst area can provide higher current density per unit area, which is conducive to the formation of C_2_ products. In contrast, if the catalyst area is too large, it will lead to a decrease in current density, which in turn affects the selectivity of C_2_ products. Reasonable area design ensures the efficient utilization of light energy and the effective separation of charges, thereby improving the selectivity and efficiency of the reaction. In order to further improve the overall performance of the system, the researchers also incorporated the glycerol oxidation reaction (GOR) in the system, which not only reduced the required photovoltage, but also increased the photocurrent density of the C_2_ part by 200 times.

**Fig. 8:**
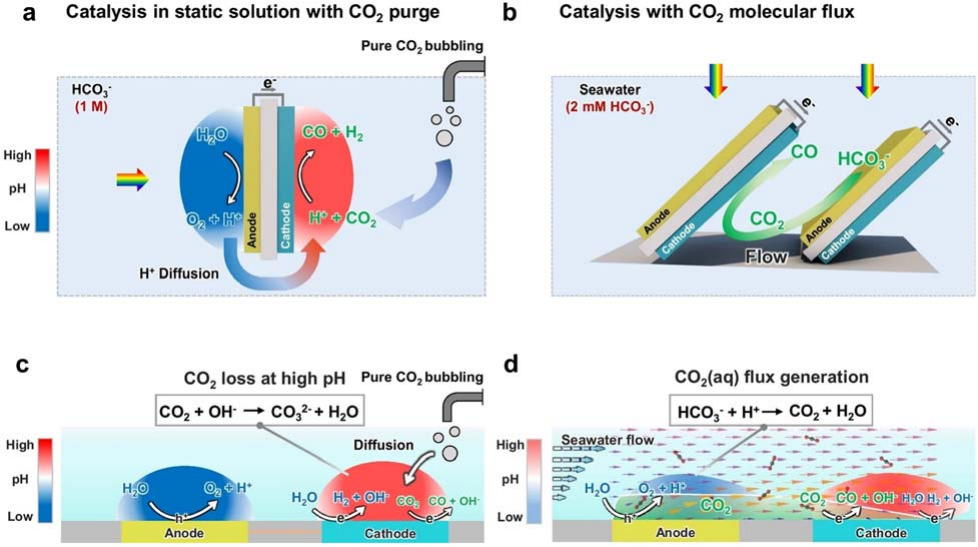
(a) Schematic diagram and (c) mechanism diagram of the traditional PEC device using static solution. (b) A schematic diagram and (d) mechanism diagram of CO_2_ flux catalysis with clear boundary layer shear flow. Reproduced with permission from ref. 109. Copyright 2025, Springer Nature.

Reasonable design of the reactor's geometric structure can optimize the light distribution and ensure that the surface of the photocatalyst is uniformly exposed to light energy, thereby avoiding the reaction inconsistencies caused by local overheating or uneven light intensity.^96^ Meanwhile, the transport path of reactants in the reactor needs to be reasonably designed to ensure that CO_2_ or other reactants can fully contact the surface of the catalyst, thus reducing the occurrence of side reactions and increasing the proportion of target products.^108^ In conventional reactors, CO_2_ transport is primarily governed by diffusion, leading to a near-zero concentration of gaseous CO_2_ in the static electrolyte.^109^ As a result, achieving high conversion efficiency and product selectivity is extremely challenging. In Fig. 8a, the electrolyte in the PEC device is static, so diffusion serves as the primary transport mechanism, followed by the generation of ionic currents carried by H^+^, HCO_3_^−^, and other major ionic species. Acid–base reactions occur during the diffusion and transport of CO_2_ (aq), H^+^, and HCO_3_^−^ (Fig. 8c). The pH gradient between the anode and cathode, ion transport, and acid–base buffering reactions constitute the main sources of transport losses. To address this, Hu's team^109^ designed a series of BiVO_4_ photoanodes arranged in parallel and inclined toward Si photocathodes, creating a boundary layer flow effect (Fig. 8b). This configuration enables continuous flow of gaseous CO_2_ reactants to the catalyst, thereby enhancing CO_2_ mass transfer. Specifically, as shown in Fig. 8d, the establishment of boundary laminar flow alters the electrolyte flow direction from the photoanode to the photocathode, and the resulting increase in H^+^ flux further reduces the pH gradient. Importantly, the continuous flux of CO_2_ is also significantly improved. The photoanode generates H^+^, which acidifies HCO_3_^−^, resulting in the *in situ* production of gaseous CO_2_ that rapidly reaches the photocathode catalyst. The customized PEC device significantly increased CO selectivity from 3% to 21%, approaching the theoretical limit of 30% predicted by the multiphysics model. Meanwhile, a solar-to-fuel (STF) efficiency of 0.71% was achieved.

**Fig. 9:**
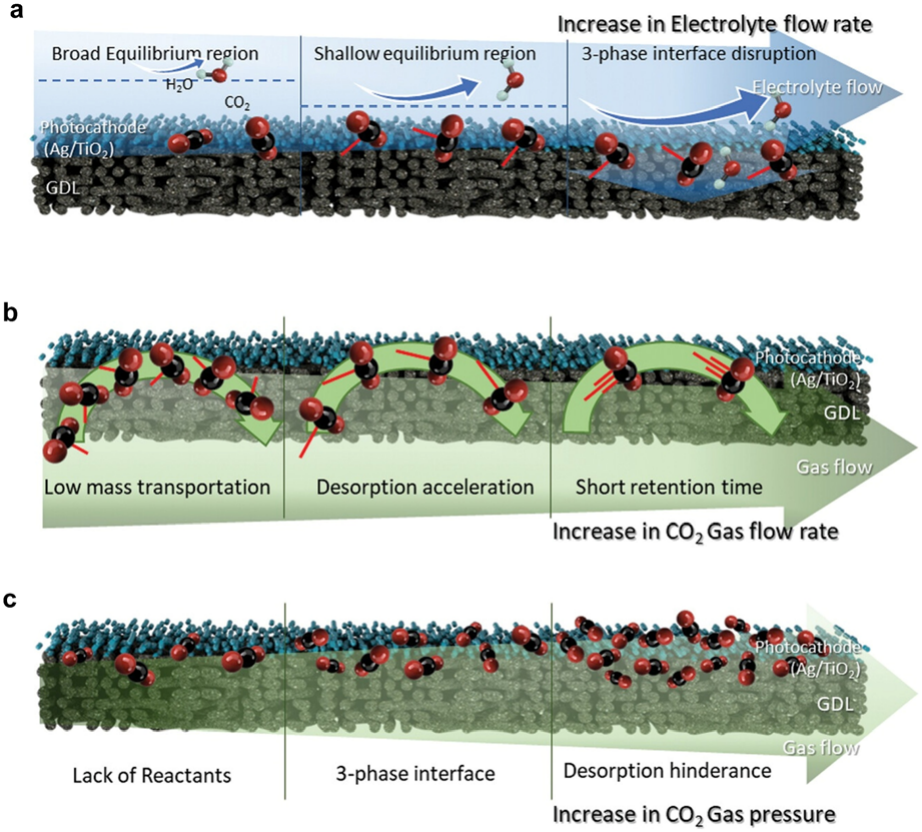
Diagrams of the surface of the gas diffusion photocathode at different (a) electrolyte flow rates, (b) gas flow rates and (c) gas pressures. Reproduced with permission from ref. 110. Copyright 2024, Wiley-VCH GmbH.

As a cutting-edge PEC device, the PEC flow cell incorporates a GDE to overcome the low solubility of CO_2_ in aqueous electrolytes, thereby significantly improving both the reaction rate and the selectivity for specific products.^39^ However, due to the unique structure of the reactor, the mass transfer of CO_2_ within the GDE is influenced by the electrolyte flow rate, the CO_2_ gas flow rate, and the CO_2_ gas pressure.^110^ As shown in Fig. 9a, a low electrolyte flow rate is insufficient to effectively remove accumulated molecules near the catalyst layer, resulting in low mass transfer efficiency at the catalyst surface. Conversely, an excessively high electrolyte flow rate increases hydraulic pressure, leading to the gradual infiltration of the electrolyte into the catalyst layer and significantly hindering mass transfer within the gas layer. Only at an appropriate electrolyte flow rate can the balance between hydraulic and gas pressures at the catalyst surface be maintained, optimally establishing the three-phase interface within the catalyst layer. In addition to the electrolyte flow rate, the CO_2_ gas flow rate is also a critical parameter. Fig. 9b illustrates that a continuous increase in the CO_2_ gas flow rate enhances the mass transfer of gaseous reactants at the catalyst surface. However, an excessively high CO_2_ flow rate reduces the residence time of CO_2_ molecules, limiting their effective adsorption, catalytic reaction, and product desorption on the catalyst surface. Furthermore, CO_2_ pressure significantly affects mass transfer, although it is often not specifically reported during testing. As shown in Fig. 9c, excessively low gas pressure hinders the passage of CO_2_ molecules through the GDE, reducing their contact with the catalyst. In contrast, excessive gas pressure disrupts the three-phase interface, disturbing the equilibrium between the adsorption and desorption of reactants and products. Maintaining optimal gas pressure is essential for sustaining an effective three-phase interface and optimizing photocathode performance. Therefore, the design of the reactor also affects the selectivity of the product by regulating the contact time of the gas–liquid interface and the flow state of the reaction medium. Appropriately prolonging the contact time or optimizing the flow state can increase the residence time of the reactant on the catalyst surface, thereby improving the selectivity of the product. However, excessive extension of the contact time may lead to an increase in by-products, so it is necessary to find a balance point in the design to achieve efficient and selective generation of the target product.

In summary, the design of PEC CO_2_RR technology requires comprehensive consideration of many factors such as the area and structure of optical devices and the design of reactors. By optimizing these design parameters, the utilization of light energy and enhancement of CO_2_ mass transfer can be maximized, the charge transfer efficiency can be improved, and the reactant contact and reaction path can be optimized, so as to achieve effective regulation of product selectivity and further improve the overall performance and efficiency of the reaction.

### 2.3 Dynamics and thermodynamics of product selectivity

The product selectivity of PEC CO_2_RR is affected by both kinetic and thermodynamic factors. The CO_2_ adsorption model, band gap structure and reaction free energy are the key influencing factors.

**Fig. 10:**
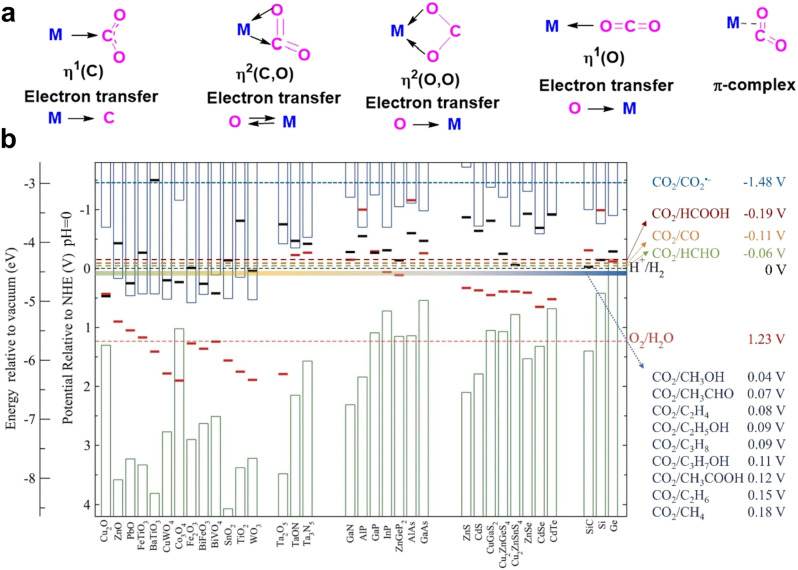
(a) Common methods of CO_2_ activation. Reproduced with permission from ref. 112. Copyright 2023, American Chemical Society. (b) The bias potentials, conduction band positions and valence band positions of various CO_2_ reduction reactions, and the calculated semiconductor oxidation and reduction potentials. Reproduced with permission from ref. 115. Copyright 2024, published by Elsevier B.V.

The first step in CO_2_ reduction is the adsorption and activation of CO_2_ on the surface sites of the catalyst, so the adsorption model also plays an important role in determining the reaction pathway and product selectivity (Fig. 10a).^111,112^ It directly affects the activation of CO_2_ molecules and the subsequent reaction path by adjusting the electronic structure and active site properties of the catalyst surface.^113^ The CO_2_ molecule reacts with the surface atoms of the catalyst through the chemical adsorption process, resulting in the mutual repulsion between C and O atoms, forming a curved CO_2_ molecule (CO_2_^−^). The formation of *CO_2_^−^ reduces the energy barrier of electron absorption, thus accelerating the electron transfer process from the electrode to *CO_2_^−^. The adsorption of CO_2_ can be divided into three main modes: C coordination, O coordination and mixed coordination. Specifically, O coordination forms a stable η^2^(O, O) coordination structure with the O atom on the catalyst surface. This adsorption mode is usually conducive to the formation of HCOOH.^90,114^ In contrast, C coordination or mixed coordination forms an incompletely activated M–CO_2_ structure by binding to metal or semiconductor sites on the catalyst surface. This adsorption mode tends to promote the further reduction of CO_2_ molecules to produce C_1_ products such as CO. Mixed coordination refers to CO_2_ binding to multiple active sites on the catalyst surface through carbon and oxygen atoms to form a more complex adsorption structure. This adsorption mode may promote a variety of reaction pathways, including the formation of C_1_ products and C_2+_ products. In addition, the adsorption mode is not only affected by the catalyst material, but also closely related to the reaction conditions. These factors work together to determine the activation degree of CO_2_ molecules and the selectivity of their subsequent reaction pathways. Therefore, the adsorption characteristics of CO_2_ on the catalyst surface not only determine the path selectivity of the subsequent reaction, but also further affect the selectivity of the final product by regulating the stability of the reaction intermediate and the reaction barrier.

The band gap structure is an important feature of the energy band characteristics of a photocatalyst. The band alignment directly determines the energy distribution of photogenerated electrons and holes (Fig. 10b).^115^ When the semiconductor material absorbs photon energy, the electrons in the valence band transition to the conduction band to form a carrier pair. At this time, the band gap width between the conduction band bottom and the valence band top not only determines the light absorption range, but also affects the driving force of the subsequent redox reaction through the initial kinetic energy of the carrier. The band gap size of the photocatalyst affects the excitation process of electrons and holes after illumination, as well as their energy matching, which determines the required energy input and the energy demand of the generated product to a certain extent. This energy transfer characteristic causes catalysts with different band gap structures to exhibit significant selectivity differences during photocatalytic reactions. Compared with the reduction potential system of the standard hydrogen electrode, it can be found that the formation of various carbon-based products corresponds to a specific energy threshold. The existence of this energy gradient requires that the conduction band position of the catalytic material must exceed the theoretical reduction potential of the target product to achieve effective conversion. That is to say, the main purpose of tuning the band structure of the photocatalyst is to control the reaction from a thermodynamic perspective. The conduction band minimum (CBM) must be sufficiently negative to provide the potential required for CO_2_ reduction.^15^ Different reduction products require different reduction potentials, so the position of the CBM must match the potential of the desired product. For example, the standard potential for the reduction of CO_2_ to CO is approximately −0.11 V *vs.* NHE (pH = 0), and a CBM < −0.11 V is more favorable for this process. Furthermore, by adjusting the band position, certain reaction pathways can be “screened” while others can be suppressed. For instance, if the CBM is positioned between the reduction potentials for the formation of CO and HCOOH, only the generation of CO is allowed, while the formation of HCOOH is inhibited. The band position of a semiconductor can be precisely regulated through band engineering strategies (such as doping,^116^ introduction of vacancies,^117^ and construction of heterostructures^118,119^) to achieve the required potential for generating the target product.

However, it is quite challenging to regulate product selectivity solely through the band structure, because many reduced products have standard reduction potentials lower than that of CO, and the standard reduction potentials of different products are often very close to each other. The surface of the catalyst supported by semiconductors possesses its own inherent “adsorption/desorption energies for reaction intermediates” (for example, gold tends to desorb CO, while copper can catalyze C–C coupling), which ultimately determines the selectivity of the catalyst toward different reaction pathways.^120^ An ideal semiconductor–catalyst combination can provide an appropriate driving force *via* the semiconductor CBM, while the catalyst surface offers suitable adsorption and activation capabilities to achieve a synergistic effect. When regulating the bandgap between the semiconductor and catalyst to achieve product selectivity, the following two principles should be considered. (1) Appropriate thermodynamic driving force: the reduction level of the electrode should match the reduction potential of the target product. In other words, the conduction band position needs to be sufficiently negative to provide the required electron energy for the reduction reaction of the product, thereby effectively driving the formation of the specific product. (2) Synergistic interfacial kinetics: the energy levels between the photocathode and the catalyst also need to be properly aligned. Only when the energy level alignment is appropriate can the photogenerated charge carriers efficiently transfer from the semiconductor to the catalyst surface, thereby promoting the catalytic reaction. If the energy levels are mismatched, the transfer efficiency of charge carriers decreases, leading to reduced catalytic activity and product selectivity. Therefore, by rationally designing the energy level structures of the semiconductor and catalyst, it is possible to effectively regulate the reaction pathway and achieve precise control over product selectivity.

The change in Gibbs free energy has a profound influence on product selectivity. The formation paths of different reduction products correspond to significantly different thermodynamic driving forces. Standard Gibbs free energy changes (Δ*G*^0^) for overall CO_2_ reduction with different products are presented in Table 1.^24^ Taking the two-electron transfer product as an example, the formation path of CO involves the formation and desorption of *COOH intermediates. The standard free energy change is 257.4 kJ mol^−1^, which shows a slight energy advantage over the 270.4 kJ mol^−1^ generated by HCOOH. This subtle energy difference can be significantly amplified by variations in the electronic structure of the catalyst surface. For example, metal catalysts such as gold and silver can effectively promote the selective formation of CO by optimizing the adsorption strength of *COOH intermediates, while metal catalysts such as Bi and Sn can effectively promote the selective formation of HCOOH. For deeply reduced products requiring multiple electron transfer, such as methanol (Δ*G* = 702.8 kJ mol^−1^) and methane (Δ*G* = 919.6 kJ mol^−1^), the reaction path involves a continuous proton-electron coupling transfer process, and the free energy changes at each step together form a complex energy barrier network. The formation mechanism of multi-carbon products shows a higher dimension of free energy correlation. Taking the synthesis of C_2_H_4_ as an example, it involves 12 electron transfer steps, and its standard free energy change exceeds 1332 kJ mol^−1^, which involves the dimerization of CO intermediates, the formation of carbon–carbon bonds and the subsequent hydrogenation process. There may be energy bottlenecks at each stage of such reactions. For example, the coverage and bonding strength of CO intermediates on the surface of copper-based catalysts directly affect the feasibility of the carbon–carbon coupling step. Meanwhile, the high free energy state of some intermediates in the reaction path may trigger competitive side reactions, resulting in a shift in product distribution to a more thermodynamically favorable direction. This requires effective stabilization of key intermediates of different products. The Gibbs free energy difference of the key intermediates (such as *CO, *CHO, *etc.*) in different reaction paths directly affects the reaction kinetics. The formation of CO requires a stable *CO intermediate, while the formation of CH_4_ involves the formation of a more stable *CHO intermediate. The stability of these intermediates directly influences the reaction pathway. If the Gibbs free energy of the *CO intermediate is low, the reaction tends to generate CO; if the Gibbs free energy of *CHO intermediate is lower, it is more conducive to the formation of CH_4_. For C_2+_ products, the key is the change in Gibbs free energy of the C–C coupling process. This process usually requires the interaction of two C_1_ intermediates (such as *CHO or *CO), and its energy demand determines the difficulty of the coupling reaction and the selectivity of the product. If the energy barrier for coupling some C_1_ intermediates is low, a specific C_2+_ product may be preferentially generated.

**Table 1:** Standard Gibbs free energy changes (Δ*G*^0^) for overall CO_2_ reduction with different products

Reaction	Δ*G*^0^ (kJ mol^−1^)
CO_2_(g) → CO(g) + 1/2O_2_(g)	257.4
CO_2_(g) + H_2_O(l) → HCOOH(l) + 1/2O_2_(g)	270.4
CO_2_(g) + H_2_O(l) → HCHO(l) + O_2_(g)	521.9
CO_2_(g) + 2H_2_O(l) → CH_3_OH(l) + 3/2O_2_(g)	702.8
CO_2_(g) + 2H_2_O(l) → CH_4_(g) + 2O_2_(g)	919.6
2CO_2_(g) + 2H_2_O(l) → CH_3_COOH(l) + 2O_2_(g)	963.8
2CO_2_(g) + 2H_2_O(l) → C_2_H_4_(g) + 3O_2_(g)	1332
2CO_2_(g) + 3H_2_O(l) → C_2_H_5_OH(l) + 3O_2_(g)	1327

In summary, the process of CO_2_ reduction is very complex. Both kinetic and thermodynamic factors determine the product selectivity of PEC CO_2_RR. In this section, the band gap structure, adsorption characteristics and thermodynamic parameters of the reaction path of the optimized photocatalyst are briefly described, so as to provide experience for the selective formation of the target product and the improvement of the reaction efficiency.

## 3 Research progress on selectivity of CO_2_ reduction products

### 3.1 C_1_ products

#### 3.1.1 CO

CO is an important industrial raw material and is widely used in the production of chemicals. As an important intermediate, CO is used in the synthesis of chemicals such as formaldehyde, formic acid, and formamide. In addition, CO can also react with H_2_ through the Fischer–Tropsch synthesis to produce various liquid fuels such as CH_3_OH, gasoline, and diesel. In the PEC CO_2_RR process, CO is the most easily obtainable product, and the formation of CO is usually unavoidable. This is mainly because the energy barrier for CO generation is relatively low, and its reaction path is relatively simple. Specifically, the generation process of CO starts from the crucial *COOH intermediate, which is further transformed into the CO intermediate, and finally desorbs from the catalytic site to form the CO molecule.^121^ This process not only involves fewer steps but also avoids the formation of complex multi-step reactions and by-products, thereby resulting in higher reaction efficiency. Another important reason is that among all carbon-containing products, the generation of CO only requires the transfer of two electrons and two protons, which is the lowest reaction requirement. In contrast, the generation of other high value-added products usually requires more electron and proton transfer steps, leading to an increase in reaction complexity and an increase in the energy barrier. Therefore, from both kinetic and thermodynamic perspectives, the generation of CO has significant advantages over other products: it proceeds with a faster reaction rate, and the required activation energy is lower, resulting in higher selectivity and yield in the photoelectrocatalytic system. Currently, a large number of studies on the PEC CO_2_RR focus on the generation of the CO product, and the FE can usually easily reach about 90%, and some studies have even achieved selectivity close to 100%. The performance of PEC CO_2_RR for CO is summarized in Table 2.

**Table 2:** Summary of the PEC CO_2_RR performance for CO

Catalyst	Electrolyte	Light source	Product and performance	Ref.
CuBi_2_O_4_–Bi_2_O_3_	0.1 M KHCO_3_	AM 1.5G of 100 mW cm^−2^	FE = 86.03% at 0.1 V *vs.* RHE	122
Bi/ZnO@ZnSe	0.5 M KHCO_3_	AM 1.5G of 100 mW cm^−2^	FE = 88.9% at −0.9 V *vs.* RHE	123
Au_25_ nanocluster inside a porphyrin nanoring	0.5 M KHCO_3_	300 W Xe lamp	FE = 92.1% at −0.67 V *vs.* RHE	124
CuGaS_2_/PPy	0.1 M KHCO_3_	300 W Xe lamp with a 420 nm cutoff filter	FE = 6% at −0.6 V *vs.* Ag/AgCl	125
HJ-Si-Ag	0.1 M KHCO_3_	AM 1.5G of 100 mW cm^−2^	FE = 97.4% at −0.2 V *vs.* RHE	126
Cu_2_O/C/PTFE	0.1 M KHCO_3_	300 W Xe lamp of 100 mW cm^−2^	FE = ∼70% at −0.7 V *vs.* RHE	127
In-doped GaN	0.5 M KHCO_3_	300 W Xe lamp	FE = ∼45% at −0.75 V *vs.* RHE	128
Molecular Re/porous Si	phenol/MeCN	AM 1.5G of 100 mW cm^−2^ 400–1100 nm	FE = 90% at −1.7 V *vs.* Fc^+^/Fc	129
CoPc/K_7_HNb_6_O_19_	0.5 M KHCO_3_	300 W Xe lamp	FE = 98.6% at −0.85 V *vs.* RHE	130
Ni–NC/Si	0.1 M KHCO_3_	AM 1.5G of 100 mW cm^−2^	FE = 100% at −0.6 V *vs.* RHE	131
ZnO/ZnTe	0.1 M TBAPF_6_-MeOH	AM 1.5G of 100 mW cm^−2^	FE = 93.88% at −2.58 V *vs.* Fc^+^/Fc	132
Fe_2_O_3_/Cu_2_O/Cu	0.1 M TBAPF_6_-MeOH	AM 1.5G of 100 mW cm^−2^	FE > 89% at −0.8 to −1.2 V *vs.* Fc^+^/Fc	133
ZnTe/SnS_2_	0.1 M TBAPF_6_-MeOH	AM 1.5G of 100 mW cm^−2^	FE = 87% at −1.78 V *vs.* Fc^+^/Fc	134
Ag-modified CuBi_2_O_4_	0.1 M KHCO_3_	AM 1.5G of 100 mW cm^−2^	FE = 92% at −0.2 V *vs.* RHE	69
CuBDC-*x*NH_2_/CF	0.1 M KHCO_3_	300 W Xe lamp with a 420 nm cutoff filter	FE = 51.5% at −0.3 V *vs.* RHE	135
STA-GO/CoPc	0.1 M KHCO_3_	300 W Xe lamp with a 400 nm cutoff filter	FE_CO_ = 86% at −0.28 V *vs.* RHE; FE_HCOOH_ = 8% at −0.62 V *vs.* RHE	136
Cu_2_O–SnO_2_	0.1 M KHCO_3_, 0.1 M KOH	AM 1.5G, 450 W Xe lamp	FE_CO_ = 37%; FE_HCOOH_ = 26%	137
PPy/Cu_2_O/FeOOH	0.1 M Bu_4_NPF_6_	AM 1.5G	FE = 90% at −1.8 V *vs.* Ag/Ag^+^	138
Cu_2_O–SnO_2_	0.1 M KHCO_3_, 0.1 M KOH	AM 1.5G of 100 mW cm^−2^	FE_CO_ = 35.47% and FE_HCOOH_ = 19.58% at −0.2 V *vs.* RHE	139
Ni–Mn–Cu–FeAl HELO	0.5 M KHCO_3_, 1 M KOH	300 W Xe lamp	909.55 μmol g^−1^ h^−1^ at −0.8 V *vs.* RHE	140
Re supramolecular	0.05 M NaHCO_3_	AM 1.5G of 100 mW cm^−2^	FE = 94%, −0.2 V *vs.* Ag/AgCl	141
Re@NH_2_-frac-MOF	0.1 M TBAPF_6_-MeOH	300 W Xe lamp with a 400 nm cutoff filter	FE = 97.9% at −0.7 V *vs.* Ag/AgCl	142
Cu@Au/SiNW	0.1 M KHCO_3_	Simulated solar light	FE = 72% at −1 V *vs.* Ag/AgCl	99
p-Si/TiO_2_/TaO_*x*_/Cu	0.1 M KHCO_3_	AM 1.5G	FE = ∼65% at −1.26 V *vs.* RHE	143
CoII(BrqPy)	0.1 M KHCO_3_	AM 1.5G of 100 mW cm^−2^	FE = 100% at −0.11 V *vs.* RHE	144
ZnGa_2_O_4_/ZnO	0.1 M NaHCO_3_	365 nm LED	FE = 28.7% at −1.4 *vs.* Ag/AgCl	89
Ag/Si or Cu/Si	1 M KOH	AM 1.5G of 100 mW cm^−2^	Ag, FE = 90%; Cu, FE_C_2+__ = 53%	145
In/Cu_2_O	0.1 M KHCO_3_	AM 1.5G of 100 mW cm^−2^	FE = 82% at −0.7 V *vs.* RHE	146
Cupric porphyrin	0.1 M TBAPF_6_-MeOH	AM 1.5G of 100 mW cm^−2^	FE = 87% at −2.5 V *vs.* Fc^+^/Fc	147
Au/GaN/Si	0.5 M KHCO_3_	100 mW cm^−2^	FE = ∼90% at 0.17 V *vs.* RHE	148
Au-NR	0.5 M KHCO_3_, 1 M KOH	AM 1.5G of 100 mW cm^−2^	FE = 85% at −1 V *vs.* RHE	70
Mn(TPP)Cl	0.1 M [NBu_4_][BF_4_]-MeCN	Halogen lamp of 90 mW cm^−2^	FE = 48.2% at −1 *vs.* Ag/AgCl	149
CuGa_3_Se_5_	0.1 M KHCO_3_	Fiber-coupled LED	FE = ∼80% at −0.4 V *vs.* RHE	150
CsAgBr_2_-PNC	Na_2_CO_3_	150 W Xe lamp	FE = 50% at −0.6 V *vs.* Ag/AgCl	151
AgCl/GaNNW/Si	0.1 M KHCO_3_	300 mW cm^−2^	FE = 80% at −0.4 V *vs.* RHE	88
Au/TiO_2_/n + p-Si	0.1 M KHCO_3_	AM 1.5G	FE = 86% at −0.8 V *vs.* RHE	152
[Cd(DPNDI)(TDC)]_*n*_	0.1 M [*n*-Bu_4_N]PF_6_–MeCN	100 W Hg lamp with a UV cut off filter	FE = 78% at −1.3 V *vs.* Fc^+^/Fc	153
CoPor-N_3_	Saturated KHCO_3_	300 W Xe lamp	FE = 96% at −0.5 V *vs.* RHE	154
Au–TiO_2_/InP	0.1 M KHCO_3_	AM 1.5G	FE = 84.2% at −0.11 V *vs.* RHE	155
Au	0.1 M TBAPF_6_-10 M H_2_O	LED white of 300 mW cm^−2^	FE = 90% at −1.68 V *vs.* Ag/AgCl	156
ZnTe	0.1 M KHCO_3_	455 nm LED	FE = 50.4% at −1.0 V *vs.* RHE	157
Si/viologen polymer/Au	0.1 M KHCO_3_	AM 1.5G of 100 mW cm^−2^	FE = 25% at 1.2 V *vs.* RHE	158
Single-atom Co–N_5_	0.5 M K_3_BO_3_	AM 1.5G of 100 mW cm^−2^	FE = 90.6% at 1.23 V *vs.* RHE	159
Si/Al-PMOF(Co)	0.1 M KHCO_3_	AM 1.5G of 100 mW cm^−2^	FE = 80.7% at −0.4 V *vs.* RHE	160
Cu(In,Ga)S_2_	0.1 M KHCO_3_	1 sun illumination	FE_CO_ = 30% and FE_HCOOH_ = 14% at −0.4 V *vs.* RHE	161
ZnTe	0.1 M KHCO_3_	AM 1.5G of 100 mW cm^−2^	FE_CO_ = 45% and FE_HCOOH_ = 15% at −0.6 V *vs.* RHE	162
Ox-Au	0.5 M KHCO_3_	1 Sun illumination	FE = ∼98% at −0.5 V *vs.* RHE	163

**Fig. 11:**
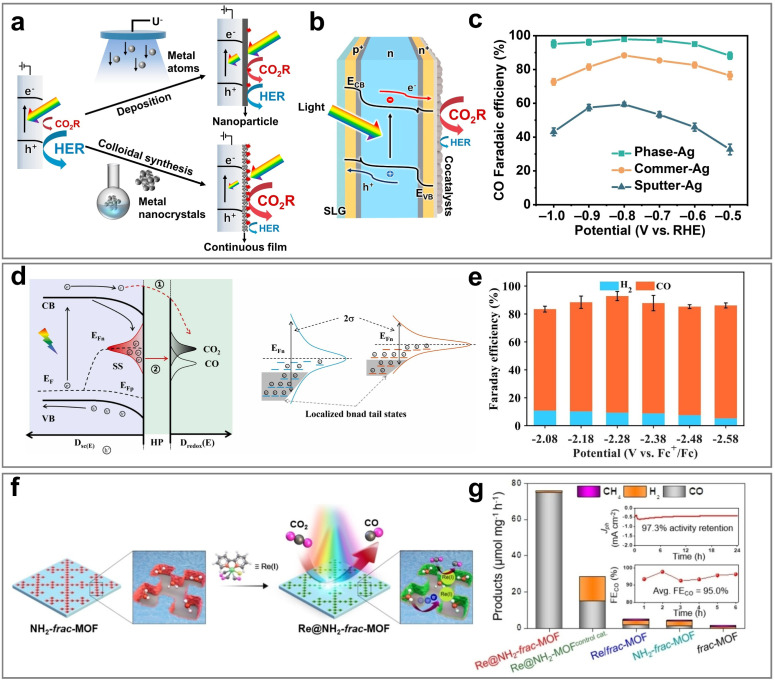
(a) Diagrammatic representation of various synthetic methodologies for co-catalyst deposition on front-side-illuminated photocathodes. (b) Structural configuration of HJ-Si photocathodes designed for CO_2_ conversion. (c) FE of CO production on different catalysts under controlled electrochemical conditions. Reproduced with permission from ref. 126. Copyright 2024, published by PNAS. (d) Energy band diagram of a photoelectrode for CO_2_ reduction to CO. (e) Adjusting the surface states by reducing disorder. Reproduced with permission from ref. 132. Copyright 2024, Elsevier B.V. All rights reserved. (f) Schematic presentation for Re@NH_2_-frac-MOF. (g) Plot of the product obtained after 6 h of PEC CO_2_RR with different catalysts and its proportion to the total mass of the catalyst. Reproduced with permission from ref. 142. Copyright 2023, Wiley-VCH GmbH.

A large number of studies have confirmed that Ag-based and Au-based catalysts show significant advantages in the CO generation path, which is mainly due to the unique characteristics of their electronic structures. Specifically, the suitable d-band center position enables the catalyst to produce a moderate adsorption effect on the key intermediates (*COOH and *CO), which not only guarantees the kinetic stability of the intermediates, but also avoids the problem of the increased desorption energy barrier caused by excessive adsorption. Combined with its weak surface adsorption characteristics (especially low binding energy to CO), the energy required for desorption is significantly reduced, thereby promoting the efficient release of CO. In addition, the weak adsorption capacity of Ag and Au for the hydrogen intermediate (*H) effectively inhibits the kinetic process of the competitive HER. The localized charge transfer between Au and TiO_2_ in the Au–TiO_2_ heterogeneous interface system enhances the binding strength of the positively charged Au interface site to the CO* intermediate, thereby optimizing the thermodynamic path of CO_2_ photoreduction to CO.^155^ The experimental results show that the FE of CO reaches 84.2% when the Au–TiO_2_/InP nanorod array photocathode is applied at −0.11 V *vs.* RHE bias under simulated AM 1.5G illumination. However, limited by the efficiency of the mass transfer process and the complex electric field environment around the active site, the activity and selectivity of the existing catalytic system still face obvious bottlenecks. Bi *et al.*^70^ innovatively used a continuous flow cell reactor coupled with plasma Au nanorods (NRs) to successfully achieve efficient PEC CO_2_ to CO conversion. Benefiting from the fast mass transfer process of flow field optimization and the local CO_2_ enrichment effect on the surface of Au NRs, the photocurrent density of the system reached 1.0 mA cm^−2^, which was nearly 5 times higher than that of the traditional H-type reactor (0.17 mA cm^−2^). At the same time, the localized surface plasmon resonance (LSPR) effect of Au NRs increased the CO FE by about 20% under illumination. For atomically precise gold nanocluster systems, their size-dependent quantum effects and ligand assembly strategies have shown unique potential in the field of CO_2_ directional conversion, but the agglomeration phenomenon that tends to occur during the catalytic process seriously restricts its stability. The research group designed a host–guest composite structure in which pyridinethiol-functionalized Au_25_ nanoclusters were embedded in six zinc porphyrin unit nanorings.^124^ The assembly not only significantly improved the stability of gold clusters, but also showed excellent activity and selectivity in the photochemical CO_2_ reduction to CO and singlet oxygen generation reaction. The FE of CO reached 92.1% at −0.67 V *vs.* RHE. Compared with Au, the cost advantage of Ag made it more practical. By combining plasmonic Ag nanoparticles with semiconductor photocatalysts, the carrier separation efficiency can be effectively enhanced. Ag nanoparticles modified CuBi_2_O_4_ inverse opal photocathode (CuBi_2_O_4_–IOs–Ag) was prepared by the sacrificial template method.^69^ The three-dimensional ordered structure optimizes both mass transfer kinetics and light capture efficiency. The ohmic contact between Ag nanoparticles and CuBi_2_O_4_ significantly improves the surface charge distribution, inhibits carrier recombination and optimizes kinetic behavior. The experimental results showed that the CO FE reached 92% at 0.2 V *vs.* RHE bias, which was 1.6 times higher than that of the original CuBi_2_O_4_ film. The crystal phase regulation of Ag nanoparticles also had a key effect on the catalytic performance. A hexagonal close-packed (hcp) crystalline Ag nanocrystal co-catalytic layer was constructed on the heterojunction silicon (Si) substrate (Fig. 11a–c).^126^ The unconventional hcp phase accelerated the charge transfer process of the CO_2_RR, and the light absorption/catalytic layer decoupling design of the Si substrate avoided parasitic light absorption. The CO FE of the photocathode exceeded 90% in a wide potential window (−0.2 V *vs.* RHE), up to 97.4%. At the CO_2_/CO equilibrium potential, the photocurrent density for CO production was −2.7 mA cm^−2^ (0.1 M KHCO_3_ electrolyte), and the FE was 96%, which was superior to that of the precious metal Au-based reference system. In addition, the ion valence regulation strategy of Ag has attracted much attention in the field of photoelectrocatalysis. The team systematically studied the CO_2_RR performance of GaN nanowire (NW)/n^+^–p–Si heterojunction photocathodes loaded with different silver halide (AgX, X = Cl, Br, I) cocatalysts.^88^ The nitrogen-terminated GaN-NW as a chemically stable substrate could effectively load silver halide, and the halogen element significantly improved the catalytic activity by reducing the energy barrier for *COOH intermediate formation. The experimental results showed that the CO FE was more than 80% at −0.4 V *vs.* RHE, and the constant potential is stable at ∼0.2 V *vs.* RHE.

In addition to precious metal systems such as Au and Ag, researchers are working on the development of low-cost and high-abundance metal-based photoelectrocatalytic materials such as Cu, Bi, Ni, Fe and Zn to build a more economical and sustainable CO_2_ conversion system. The In/Cu_2_O core–shell nanowire Schottky junction constructed by physical vapor deposition exhibited a space charge region reconstruction effect, which significantly optimized the photogenerated carrier dynamics.^146^ Experiments showed that the heterostructure increased electron mobility by 2.3 times, achieved a CO yield of 75.94 μmol cm^−2^ h^−1^ at −0.7 V *vs.* RHE bias, and maintained 12 hours of continuous operation (FE = 82%). Combined with *in situ* Fourier transform infrared spectroscopy (*in situ* FTIR) analysis, it was confirmed that the introduction of In promoted the CO desorption kinetics by enhancing the adsorption energy of the COOH* intermediate (Δ*E*_ads_ = −1.2 eV). Gao's team^138^ innovatively constructed a three-level functional Cu_2_O-based photocathode system: (1) the Cu_2_O structure was used for efficient light collection, and the light capture efficiency was increased to 92%; (2) the FeOOH hole transport layer enabled directional carrier migration; (3) the PPy modification layer provided continuous multi-electron transfer channels. The integrated system achieved a record CO yield of 46.17 mmol h^−1^ at −2.0 V *vs.* Ag/AgCl voltage, a half-cell solar-to-fuel conversion efficiency (hSTC) of 1.58%, a quantum efficiency breakthrough of 3.08%, and extended continuous operation stability to 7 h. In view of the limitations imposed by semiconductor/electrolyte interface states on energy conversion efficiency, the surface modification of ZnTe with an ultra-thin ZnO layer in the ZnO/ZnTe gradient band heterojunction reduces energy disorder and improves the electron utilization rate in the CO_2_ reduction reaction (Fig. 11d and e).^132^ In a CO_2_-saturated acetonitrile system containing 0.1 M tetrabutylammonium hexafluorophosphate (TBAPF_6_), the photocathode achieved a current density of −7.70 mA cm^−2^ and a CO selectivity of 93.88% at a potential of −2.58 V *vs.* Fc+/Fc. The precise construction of single-atom catalysts provided a new paradigm for the regulation of active sites. The Ni single atom anchored on a silicon nanowire substrate enabled CO_2_ reduction to achieve nearly 100% CO selectivity at −0.6 V *vs.* RHE potential, and the TOF value (3.2 × 10^−2^ s^−1^) was two orders of magnitude higher than that of the nanoparticle system.^131^ The introduction of single-atom nickel sites provided sufficient active sites for CO_2_ reduction, thereby improving the selectivity of CO formation.

Molecular catalysts are a class of catalysts with well-defined active sites and precisely tailored structures.^164^ The active center is usually composed of mononuclear or multinuclear metal ions (such as Fe, Co, Ni, *etc.*) or specific organic functional groups, and is precisely regulated by the coordination group and the surrounding chemical environment. In the PEC CO_2_RR system, such catalysts can act as photosensitizers or catalytically active sites, directly participate in the transport of photogenerated carriers and the chemical conversion of CO_2_ molecules, and generally exhibit excellent CO product selectivity. In the past five years, the molecular catalysts commonly used in the PEC CO_2_RR are molecular Re catalyst,^142^ molecular Co catalyst,^130^ molecular Mn catalyst,^149^ molecular Pd catalyst.^141^ The low-temperature calcination process realized the coupling of cobalt phthalocyanine (CoPc) with oxygen vacancy-enriched K_7_HNb_6_O_19_, and successfully constructed a Z-scheme heterojunction structure and applied it to the photoelectrocatalytic reduction of CO_2_ to CO.^130^ Experiments showed that the heterojunction catalyst exhibits >90% FE and significantly improved turnover frequency (TOF) in a wide potential window of −0.65 to −1 V (*vs.* RHE). Its excellent PEC performance stemmed from two key designs: first, the intrinsic electric field formed by the Z-scheme heterojunction interface significantly enhanced the separation efficiency of photogenerated electron–hole pairs; second, the oxygen vacancies in K_7_HNb_6_O_19_ not only optimized the activation path of CO_2_ adsorption, but also effectively inhibited the agglomeration of CoPc molecules through the steric hindrance effect, thereby improving the structural stability of the catalyst. Density functional theory calculations further confirmed that the synergistic effect of oxygen vacancies and CoPc significantly reduced the CO_2_ activation energy barrier. Li's research group^144^ innovatively immobilized the cobalt complex CoII(BrqPy) (BrqPy = 4′,4′-bis(4-bromophenyl) −2,2′:6′,2′′:6′′,2′′′-quaternary pyridine) on the surface of a multi-walled carbon nanotube (CNT) modified TiO_2_ protective layer on an *n*-type Si electrode (Si|TiO_2_) *via* π–π stacking. Under standard solar light intensity, the composite catalytic system was continuously operated in a neutral electrolyte for 2 h to obtain a photocurrent density of 1.5 mA cm^−2^, and achieve 100% CO selectivity and FE. The performance improvement was attributed to the high dispersion of molecular active sites and CNT-mediated fast charge transfer channels. In order to further improve the loading efficiency of molecular catalysts, Lee's research group^142^ developed a lattice-guided anisotropic etching strategy based on 2D MOF-5 nanocrystals, and successfully constructed a carrier material with hierarchically ordered nano-mesoporous structure for the stable loading of Re(bpy)(CO)_3_Cl complexes (Fig. 11f and g). The highly exposed active sites at the boundary of the fractal pores enabled the catalyst to achieve a CO yield of 74.8 μmol mg^−1^ h^−1^ and a FE_CO_ of 97.9% at −0.7 V (*vs.* Ag/AgCl), while maintaining a continuous and stable photocurrent output for 24 h. The important role of microscopic pore engineering in improving the loading density and interfacial mass transfer efficiency of molecular catalysts was revealed.

**Fig. 12:**
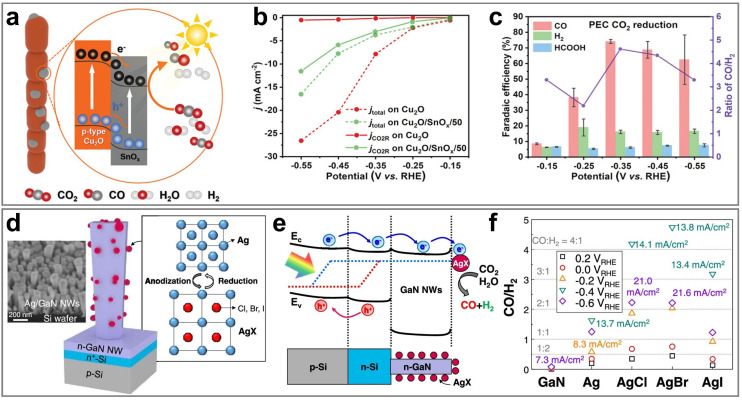
(a) Schematic illustration of the Cu_2_O/SnO_*x*_ heterojunction for PEC CO_2_ reduction. (b) Total current densities and partial current densities. (c) FE of different products and the proportions of syngas. Reproduced with permission from ref. 168. Copyright 2021 Wiley-VCH GmbH. (d) Preparation mechanism diagram of the AgX catalyst (X = Cl, Br, I). (e) Energy band diagram. (f) CO/H_2_ ratio diagram of different photocathodes at different potentials from 0.2 to −0.6 V *vs.* RHE. Reproduced with permission from ref. 88. Copyright 2022, American Chemical Society.

In the process of CO formation, although there is a problem that the competitive HER leads to a decrease in product selectivity, the controllable preparation of syngas can be realized by reasonably regulating the yield ratio of CO to H_2_. Syngas is a key chemical raw material composed of H_2_ and CO, and its molar ratio regulation plays a decisive role in downstream industrial applications.^173^ The performance of PEC CO_2_RR for syngas is summarized in Table 3. For example, the molar ratio of H_2_/CO in the Fischer–Tropsch synthesis process needs to be 2 : 1, while in methanol synthesis the ratio should be regulated in the range of 2 : 1–3 : 1. Based on this, the dynamic adjustment of syngas components through reaction condition optimization and catalyst structure design has become the focus of current research. The band-matched SnO/g-C_3_N_4_ p–n heterojunction catalyst exhibited significantly improved light absorption efficiency (*λ* > 420 nm) and photogenerated carrier separation efficiency.^42^ Under an applied bias of −1.5 V *vs.* RHE, the system achieved a total FE of 70%, and the H_2_/CO ratio could be linearly changed in the range of 1.5–10 by voltage regulation. As shown in Fig. 12a–c, the Cu_2_O–SnO_*x*_ hybrid nanowire (NW) photocathode achieved a total FE of 90.32% at −0.35 V (*vs.* RHE), and the CO/H_2_ ratio could be precisely controlled in the range of 2.2 : 1 to 4.6 : 1.^168^ The mechanistic study showed that the electrochemical deposition of SnO _x_ on the surface of Cu_2_O NWs significantly improved the charge transport kinetics and CO_2_ adsorption capacity (about 3.8 times), and photoexcitation accelerated the formation kinetics of *COOH intermediates, thus promoting the efficient desorption of CO. As shown in Fig. 12d–f, Ag and AgX (X = Cl, Br, I) cocatalysts were loaded on the surface of the GaN NWs/Si heterojunction photocathode.^88^ Compared with the pure Ag system, AgCl and AgBr increased the CO selectivity from 62% to 82%, and the AgX/GaN/Si system exhibited an onset potential shift of 0.2 V (*vs.* RHE) and a current density of > 20 mA cm^−2^ at −0.6 V (CO/H_2_ > 2). It is worth noting that the AgBr/GaN/Si cathode in the flow cell achieved a photocurrent density of 92 mA cm^−2^ under 3 solar intensity (300 mW cm^−2^), and the CO/H^2^ ratio remained stable for 12 h. The Ag_3_Cu/TiO_2_/ZnTe metal–insulator–semiconductor (MIS) photocathode system exhibited a significant and stable photocurrent density of 5.10 mA cm^−2^ at 0.20 V (*vs.* RHE) under AM 1.5G illumination, and its CO : H_2_ generation ratio could reach up to 6.8, showing excellent syngas composition control ability.^169^

**Table 3:** Summary of the PEC CO_2_RR performance for syngas

Photocathode	Electrolyte	Light source	Product and performance	Ref.
PCE10:EH-IDTBR	0.5 M KHCO_3_	AM 1.5G of 100 mW cm^−2^	(Syngas) 15 mA cm^−2^ at 1.23 V *vs.* RHE	83
Sn/SnO_*x*_–Cu_2_O/Ga_2_O_3_/TiO_2_	100 mM SnCl_2_	AM 1.5G	FE_syngas_ = 62%, CO : H_2_ ratio: 0.5; FE_HCOOH_ = 32% at −1 V *vs.* RHE	165
SnO/g-C_3_N_4_	Na_2_SO_4_	100 mW cm^−2^	FE_syngas_ = 70% at −1.3 V *vs.* RHE	42
Au/TiO_2_/GaN/Si	0.5 M KHCO_3_	AM 1.5G of 100 mW cm^−2^	FE_syngas_ = 100% at 0.17 V *vs.* RHE, CO : H_2_ ratio: ∼1	166
Au	2 M KOH	AM1.5G	CO : H_2_ ratio: 10–20	81
Cu_92_In_8_|GE|BiOI	0.5 M KHCO_3_	AM 1.5G of 100 mW cm^−2^	CO : H_2_ ratio: 3.7 at −0.15 V *vs.* RHE	167
Cu_2_O–SnO_*x*_	0.5 M NaHCO_3_	300 W xenon lamp with a 420 nm cutoff filter providing a light intensity of 200 mW cm^−2^	FE_syngas_ = 90.32% at −0.35 V *vs.* RHE, CO/H_2_: 2.2–4.6	168
AgCl/GaNNW/Si	0.1 M KHCO_3_	300 mW cm^−2^	CO/H_2_ ratio > 4 with a high current density >13 mA cm^−2^	88
Ag/Cu_*x*_O@rGO/Mo	0.1 M KHCO_3_	AM 1.5G of 200 mW cm^−2^	CO : H_2_ ratio: 0.65–1.84	80
Ag_3_Cu/TiO_2_/ZnTe	0.1 M KHCO_3_	AM 1.5G	CO : H_2_ ratio: 6.8 at −0.2 V *vs.* RHE	169
CuIn_0.3_Ga_0.7_S_2_	MeOH–MeCN	AM 1.5G	CO : H_2_ ratio: 6.24 at −1.86 V *vs.* Fc^+^/Fc	82
Ag	0.5 M KHCO_3_	Visible light (100 mW cm^2^)	(Syngas) 124.47 μmol (cm^−2^ h) at 1.57 V *vs.* RHE	170
	1 M KOH			
FeNiO_*x*_/WO_3_/W	0.5 M Na_2_SO_4_	300 W Xe lamp with a 420 nm cutoff filter	CO : H_2_ ratio: 0.5 at −1.05 V *vs.* RHE	171
ZnS–Cu_0.8_Ag_0.2_GaS_2_	0.1 M KHCO_3_	300 W Xe-arc lamp (>420 nm)	FE_syngas_ = 95% at 0 V *vs.* RHE	172

In photoelectrocatalytic carbon dioxide reduction systems, the CO formation pathway has become one of the main research directions in this field due to its low reaction energy barrier and excellent selectivity. Studies have shown that noble metal catalysts (such as Ag and Au) can significantly reduce the reaction activation energy and optimize product selectivity due to their moderate adsorption characteristics of key intermediates (*COOH and *CO), thus exhibiting excellent performance in CO selective generation. In recent years, the research focus has gradually shifted to non-precious metal-based catalyst systems such as Cu, Bi and Ni, aiming to reduce production costs and improve process sustainability through material innovation. It is noteworthy that the construction of single-atom catalysts and the precise loading strategy of molecular catalysts have been proven to be effective technical approaches to achieve near 100% FE. By rationally designing the photoelectrocatalytic system, such as optimizing the surface characteristics of the catalyst, improving the light absorption capacity and enhancing the charge separation efficiency, the efficiency and stability of CO generation can be significantly improved. Although the competitive formation of H_2_ and CO may affect the product selectivity, the ratio of CO to H_2_ can be precisely controlled by adjusting the reaction conditions and catalyst design, so as to meet the needs of downstream processes such as syngas. In general, the generation of CO in PEC CO_2_RR has significant advantages. With the further optimization of catalyst performance and the improvement of reaction conditions, this technology is expected to achieve greater breakthroughs in efficiency and selectivity in the future.

#### 3.1.2 HCOOH

As an important industrial chemical and energy carrier, the market value of HCOOH is 0.74 $ per kg, which is significantly higher than that of CO. Formic acid is not only an important intermediate for the synthesis of formate, formamide and other chemicals, but can also be used as a hydrogen storage carrier (releasing H_2_ by dehydrogenation) or directly applied to formic acid fuel cells. In the field of carbon dioxide reduction, formic acid has gradually become the focus of research and is regarded as a potential product with high added value. The formation path is based on the adsorption of CO_2_ molecules and the formation of key *OCHO intermediates, which are directly converted into formic acid through a two-step proton-coupled electron transfer process. The reaction requires only two electrons and two protons, with a low energy barrier and simple reaction steps, effectively avoiding complex multi-step reactions and the formation of by-products. Therefore, the key to the formation of HCOOH is to effectively stabilize the *OCHO intermediate. If the *COOH intermediate is formed, it is more likely to produce other products such as CO. Sn, Bi, and In-based catalysts exhibit high formic acid selectivity due to their stable adsorption of OCHO intermediates and inhibition of the HER. Therefore, the research on the efficient and selective generation of HCOOH is gradually increasing, and some studies have shown that FE can reach 100%. The performance of PEC CO_2_RR for HCOOH is summarized in Table 4.

**Table 4:** Summary of the PEC CO_2_RR performance for HCOOH

Catalyst	Electrolyte	Light source	Product and performance	Ref.
VS-ZnIn_2_S_4_/TiN	0.1 M KHCO_3_	AM 1.5G of 200 mW cm^−2^	FE = 81% at −0.45 V *vs.* RHE, 1173.2 μM h^−1^ cm^−2^	174
In–Bi_2_O_3_/Cu_2_O	0.5 M KHCO_3_, 0.5 M KOH	300 W Xe lamp with a 420 nm cutoff filter	FE = 97.8% at −0.87 V *vs.* RHE	175
STO/a-R TNTAs	0.5 M Na_2_SO_4_	300 W Xe lamp of 1240 mW cm^−2^	FE = 90.70%	33
FeOOH/p–nCu_2_O/Co:CdS	0.5 M KHCO_3_	AM 1.5G, 350–800 nm	FE = 82.9% at −0.75 V *vs.* RHE	176
Bi-doped InOCl	0.5 M NaHCO_3_	300 W Xe lamp	FE = 89.9% at −0.9 V *vs.* RHE	177
Bi	0.5 M KHCO_3_	LED lamp, 450 nm of 100 mW cm^−2^	FE = 79.1% at −1.8 V *vs.* Ag/AgCl	178
CuBi-MOF/Si	0.5 M KHCO_3_	300 W Xe lamp with a 420 nm filter providing an intensity of 200 mW cm^−2^	FE = 95% at −0.3 V *vs.* RHE	179
2D Bi/Bi_2_O_2_CO_3_	0.5 M KHCO_3_	Xe lamp	FE = 92.68% at −0.95 V *vs.* RHE	77
LD-Bi	0.5 M KHCO_3_	AM 1.5G of 100 mW cm^−2^	FE = 93.8% at −2.2 V *vs.* RHE	180
4:1-BiVO_4_/ZIF-8	0.1 M KHCO_3_, 0.05 M KOH	300 W Xe lamp	FE = 91.24% at −0.9 V *vs.* RHE	181
Formate dehydrogenase	0.067 M NaHCO_3_	AM 1.5G, 3 sun	FE = 97%	96
CuSe@BiOI	0.5 M KHCO_3_	AM 1.5G of 150 mW cm^−2^	FE = 80.5% at −1.2 V *vs.* RHE	65
Sn–Bi_2_O_3_	0.5 M KHCO_3_, 0.5 M KOH	AM 1.5G of 100 mW cm^−2^	FE = 94%	182
Bi–Sn/SiNW	0.1 M KHCO_3_	AM 1.5G of 100 mW cm^−2^	FE = 88.67% at −1.02 V *vs.* RHE	183
PCN-*n*-SiO_*x*_	0.1 M KHCO_3_	AM 1.5G	283.0 μmol L^−1^ at −0.8 V *vs.* OCP	184
Bi_2_Se_3_/Bi_2_O_3_	0.5 M KHCO_3_, 0.5 M KCl	300 W Xe lamp	FE = 81.33% at −0.9 V *vs.* RHE	185
Cu8%-BiVO_4_	1 M Na_2_SO_4_	300 W Xe lamp	FE = 87.15% at −1 V *vs.* RHE	186
TiO_2_/Ti_3_CN Mxene	0.1 M KHCO_3_	300 W Xe lamp	45.6 μM cm^−2^ h^−1^ at −0.8 V *vs.* SCE	187
Bi/GaN/Si	0.5 M KHCO_3_, 1 M KOH	AM 1.5G of 100 mW cm^−2^	FE = 85.2% at −0.2 V *vs.* RHE	188
Hybrid CuGaO_2_	0.3 M BMIM·TfO-MeCN	450 W Xe lamp with an AM 1.5G filter	FE = 81% at ^−1.5^ mA cm^−2^	189
CuFe_2_O_4_/CFs-DSAC	1.5 M KHCO_3_	Visible light	FE = 52% at −0.8 V *vs.* RHE	190
Bi@ZFOPEC	0.1 M KHCO_3_	300 W Xe lamp	FE = 61.2% at −0.65 V *vs.* RHE	60
Bi/GaN/Si	0.1 M KHCO_3_	AM 1.5G of 100 mW cm^−2^	FE = 98% at −0.3 V *vs.* RHE	191
Bi–Bi_2_O_3_/ZnO/p-Si	0.1 M KHCO_3_	AM 1.5G of 200 mW cm^−2^	FE = 84.3% at −0.95 V *vs.* RHE	192
TOAgx	0.5 M K_2_SO_4_	300 W Xe lamp	FE = 73% at −1.2 V *vs.* Ag/AgCl	193
10-Bi_2_S_3_/ZIF-8	0.5 M NaHCO_3_	300 W Xe lamp with a 420 nm cutoff filter	FE = 74.2% at −0.7 V *vs.* RHE	194
g-C_3_N_4_/SnO_2_	0.5 M NaHCO_3_	300 W Xe lamp	FE = 58% at −0.5 V *vs.* RHE	195
Bi_2_CuO_4_	0.5 M KHCO_3_, 0.5 M Na_2_SO_4_	AM1.5G filter	273.56 μmol cm^−2^ h^−1^ at −0.9 V *vs.* RHE	196
CoPcS/GO-COO	0.1 M KHCO_3_	AM 1.5G of 100 mW cm^−2^	FE = 83.9% at −1.0 V *vs.* Ag/AgCl	197
Cu–Bi_2_Se_3_	1 M Na_2_SO_4_	300 W Xe lamp	FE = 65.31% at −0.5 V *vs.* RHE	198
Cu_2_O-AF-PSi	0.2 M Na_2_SO_4_	1 Sunlight	FE = 61% at −0.3 V *vs.* Ag/AgCl	199
Cu–SnO_2_/ZIF-8	0.5 M NaHCO_3_	300 Xe lamp	FE = 68.96% at −0.364 V *vs.* RHE	200
Ni@In/SiNW	0.10 M KHCO_3_	AM 1.5G of 100 mW cm^−2^	FE = 87% at −1.2 V *vs.* RHE	31
IO-TiO_2_|FDH	86 mM MOPS, 50 mM NaHCO_3_, 50 mM CsCl	AM 1.5G of 100 mW cm^−2^	FE = 83% at 0.4 V *vs.* RHE	100
Cu_6_Sn_5_	0.05 M H_2_SO_4_	AM 1.5G of 100 mW cm^−2^	FE = 61% at −0.91 V *vs.* SHE	201
Si/dT/Bi	0.10 M KHCO_3_	AM 1.5G of 100 mW cm^−2^	FE = 82.7% at −0.2 V *vs.* RHE	202
BiVO-/ZIF-8	0.5 M NaHCO_3_	300 W Xe lamp	FE = 91.24% at −0.9 V *vs.* RHE	119
Mn complexes	2 M triethylamine and 2 M isopropanol-MeCN	1 Sun illumination	FE = 96% at −1.75 V *vs.* Fc^+^/Fc	203
Cu_2_O/Ga_2_O_3_/TiO_2_/In	0.10 M KHCO_3_	AM 1.5G	FE = 62.1% at −0.9 V *vs.* Ag/AgCl	204
OPV|IO-TiO_2_|FDH + CA	0.05 M NaHCO_3_–KCl	AM 1.5G	FE = ∼100% at 0.6 V *vs.* RHE	205
ITO-CF|FDH_NvH_	0.1 M MOPS-DMSO-NAD + -acetophenone	AM 1.5G of 100 mW cm^−2^	FE = 99% at −0.25 V *vs.* RHE	206
CNX-ITO|FDH	100 mM NaHCO_3_, 50 mM KCl, and 7.5 mM MBA	AM 1.5G of 100 mW cm^−2^	FE = 94%	207
Ultra-thin TiO_2_	0.10 M KHCO_3_	1 Sun illumination	FE = ∼98% at −0.5 V *vs.* RHE	208

**Fig. 13:**
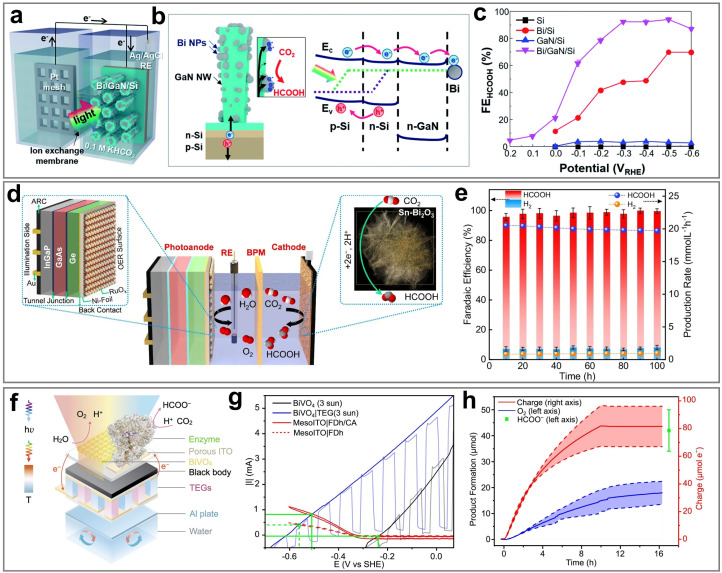
Schematic diagram of (a) PEC CO_2_ RR and (b) energy band diagram. (c) FE of HCOOH with different samples. (d) Mechanistic demonstration of the PEC device during the CO_2_RR. Reproduced with permission from ref. 191. Copyright 2022, The Royal Society of Chemistry. (e) FE of HCOOH and H_2_ production rates with the Sn–Bi_2_O_3_ NCs cathode. Reproduced with permission from ref. 182. Copyright 2024, Springer Nature. (f) The device for CO_2_RR to HCOO^−^. (g) Cross-over plots under 3-sun irradiation. (h) Product detection from PEC experiments. Reproduced with permission from ref. 96. Copyright 2024, published by Elsevier Inc.

As a star candidate material in the field of photoelectrocatalytic reduction of CO_2_ to formate, Bi-based materials have excellent properties due to their unique electronic structure characteristics. The binding energy between the 5p orbital of Bi and the key intermediate OCHO is in the optimal range, which can not only promote the stabilization of the intermediate by moderate adsorption, but also avoid the difficulty of product desorption caused by excessive adsorption, thus effectively reducing the reaction energy barrier. In addition, the weak adsorption of H on the Bi surface significantly inhibits the HER, leading to the preferential flow of electrons to the CO_2_ reduction path. At present, the main research systems include pure Bi-based, Bi–Cu-based, Bi–Sn-based, *etc.*, and performance optimization is achieved through strategies such as alloying, doping control, and heterostructure construction. In terms of interface engineering research, Dong *et al.*^191^ revealed the enhancement mechanism of interfacial electron interaction on catalytic activity by constructing a bismuth nanoparticle (Bi-NP)/gallium nitride nanowire (GaNNW) interface system (Fig. 13a–c). Theoretical calculations confirmed that the Bi-GaN binary system can optimize the synthesis pathway of HCOOH and enhance the electron transfer efficiency from GaNNWs to Bi–NPs. The experimental results showed that the optimized Bi/GaN/Si photocathode has a FE_HCOOH_ of 98% under standard AM 1.5G illumination conditions and −0.3 V *vs.* RHE potential. The nanosheet structure of Bi/Bi_2_O_2_CO_3_ composite films prepared *in situ* significantly improved the reaction kinetic parameters.^77^ At −0.95 V *vs.* RHE potential, the FE of HCOOH reached 92.68%, and the performance remained stable over 10 h. It is worth noting that its highest bias photon current efficiency (1.19%) and cathode energy efficiency (61%) indicate that the catalyst has excellent energy conversion ability. In addition, the interaction between Bi and Cu elements also contributes to the formation of HCOOH. Cu_2_O@Bi-300/Si photocathodes were prepared using Bi-MOF as a template.^179^ The characterization showed that the bridging effect of Cu–O–Bi bonds and the synergistic effect of coordinated unsaturated Bi sites lead to excellent carrier separation efficiency. Under the condition of −0.3 V *vs.* RHE, the formation rate of formate reached 101.1 μmol cm^−2^ h^−1^ (FE = 95%), which was 10.2 times higher than that of the Bi-300/Si system, and the stability was maintained for 50 h. Combined with *in situ* FT-IR data and DFT calculations, it was confirmed that Cu_2_O-induced electron-rich Bi sites can enhance the chemical adsorption of CO_2_ and stabilize key intermediates. The CuSe@BiOI heterojunction system was regulated using an epitaxial growth strategy, and its BiOI (102) anisotropic charge transport properties significantly improved the charge separation efficiency.^65^ In 0.5 M KHCO_3_ electrolyte, the system exhibited a formate selectivity of 80.5%. In-based and SN-based materials have also demonstrated potential for HCOOH production, and are often combined with Bi-based materials to enhance HCOOH selectivity. As shown in Fig. 13d and e, the integrated system comprising an InGaP/GaAs/Ge photoanode and a Sn-modified BiO_*x*_ cathode achieved 100 h of continuous operation in a non-auxiliary two-electrode system, with an average FE of 88%, a yield of 17.3 mmol L^−1^ h^−1^, a solar-fuel conversion efficiency (STF) of 12%, and an electrical energy efficiency (EE) of 60%.^182^ The mechanism analysis showed that the metal-semiconductor heterostructure formed by the Sn–BiO_*x*_ interface can optimize the electron transport path. At the potential of −1.02 V *vs.* RHE, the FE of formate reached 88.67%, and the yield was 80.07 μmol h^−1^ cm^−2^.^69^ The band structure analysis confirmed that the cascade band structure formed by Bi–Sn alloy and Si nanowires significantly promotes photogenerated electron migration. The In-doped Bi_2_O_3_/Cu_2_O foam photocathode had a three-dimensional hierarchical nanoflower structure, which maintained FE_HCOOH_ ≥ 90% in a wide potential window of −0.87 ∼ −1.17 V *vs.* RHE, with a peak efficiency of 97.8% (*J* = 14.41 mA cm^−2^).^175^ The oxygen vacancies induced by In doping and the Bi_2_O_3_/Cu_2_O heterojunction synergistically enhance the efficiency of intermediate adsorption and charge separation.

In addition to Bi-based materials, the material systems based on Cu, In, Sn, and Cd can also achieve highly selective catalytic conversion of HCOOH products through component optimization and structural regulation. The bifunctional Cu–SnO_2_/ZIF-8 catalyst constructed by the combination of hydrophobic ZIF-8 and hydrophilic Cu–SnO_2_ enables the direct use of gas phase CO_2_ to improve the molecular activation efficiency and effectively inhibit the HER.^200^ Under an overpotential of −364 mV *vs.* Ag/AgCl, the FE of formic acid increased to 68.96%, and a peak current density of 12.8 mA cm^−2^ was obtained at −1.4 V *vs.* Ag/AgCl. The FeOOH/Cu_2_O/Co:CdS multistage heterojunction photocathode constructed by sequential electrodeposition combined with chemical bath deposition showed significantly enhanced photovoltage, which was 1.9 times higher than that of the existing photocathode, and the FE of CO_2_ reduction to formic acid reached 82.9%.^176^ The Co:CdS quantum dots on the outer layer of the photocathode significantly promoted formic acid formation by introducing additional hybrid orbitals that enhance the binding energy to the key intermediate *OCHO. In addition, doping Co into the composite material containing CdS introduces impurity levels, which not only reduce the band gap of the photocathode, but also improve its visible light absorption capacity, thus significantly improving the photochemical properties. Wei *et al.*^174^ proposed a new strategy for constructing VS-ZnIn_2_S_4_/TiN-*x* foliated heterojunctions. Due to its plant cell-like morphological characteristics and enhanced electron mobility from the heterojunction interface to surface active sites, the system demonstrated excellent catalytic performance with a product formation rate of 1173.2 μM h^−1^ cm^−2^.

Organic catalyst systems, including organic polymers, molecular catalysts, biological enzyme catalysts, *etc.*, show high reduction selectivity toward HCOOH products. The composite photocatalyst of polymer carbon nitride (PCN) and defective nano-silica (*n*-SiO_*x*_) has a higher specific surface area, improved photoresponse and reduced charge recombination.^184^ The synergistic effect of the PCN-*n*-SiO_*x*_ interface promoted photoinduced charge transfer from PCN to *n*-SiO_*x*_, which inhibited carrier recombination. The resulting PCN-*n*-SiO_*x*_ composite was three times more effective in reduction of CO_2_ to methanol and formic acid. In addition, during the CO_2_ reduction process, the composite selectively produced 283.0 μmol L^−1^ formic acid, which was five times higher than that produced using bare PCN (57.0 μmol L^−1^). Nandal *et al.* combined cobalt tetrasulfonamide phthalocyanine (CoPcS) with carboxylated graphene oxide.^197^ The chemical connection of the CoPcS complex unit to the carboxylated GO carrier effectively provided enough interfacial area to promote the adsorption of CO_2_ and increase CO_2_ concentration, thus achieving a higher conversion rate. Under simulated sunlight irradiation, a high current density (−1.5 mA cm^−2^) was obtained at a voltage of −1.0 V *vs.* Ag/AgCl, and the selective formation rate of formate was 2.35 mmol h^−1^ cm^−2^. As shown in Fig. 13f–h, Cobb *et al.*^96^ innovatively used formate dehydrogenase (FDh) as a model catalyst to achieve the selective conversion of CO_2_ to formate under minimum overpotential. The catalytic activity of the FDh cathode and BiVO_4_ photoanode was facilitated by solar heating, which allowed the non-biased semi-artificial FDH-TE-BIVO_4_ device to produce HCOOH with a FE of 97% under 3 Suns of solar irradiation.

The formation pathway of HCOOH has the advantages of simple reaction steps and a low thermodynamic barrier. Its formation mechanism mainly depends on the stabilization of key intermediates *OCHO or *COOH in the CO_2_ reduction process, and then the formation of HCOOH. And the selective formation of formic acid faces less competition from by-products. Therefore, the available results show high selectivity. The Bi-based catalysts (including their alloying structure, heterojunction complex and doping modification) have become promising materials to achieve highly selective formic acid generation (FE > 90%). Other materials such as Cu base, In-base and Sn-base also show good formic acid selectivity, and some materials can achieve FEs close to 90% after reasonable design. It is worth noting that systematic studies of non-metallic catalysts (such as organic polymers, molecular catalysts and bioenzyme catalysts) have also confirmed their significant selective advantages, providing an important research direction for the development of efficient and low-cost catalytic systems. In general, with the continuous development of catalyst design, photoelectric catalytic CO_2_ reduction to formic acid has shown considerable application potential.

#### 3.1.3 CH_3_OH

As an important C_1_ platform compound, methanol has important application value in the pharmaceutical industry, polymer material synthesis and clean energy fields. As a new fuel substitute and a highly efficient hydrogen carrier, methanol has shown significant advantages in the energy transition strategy. Compared with CO and HCOOH, the six-electron reduction process of methanol is more complex. The reaction path involves a continuous multistep proton–electron cooperative transfer mechanism. Key reaction intermediates include the surface adsorbed states *COOH, *CHOH, *CH_2_OH, *etc.* The whole reduction process requires the coordinated transfer of 6 e^−^ and 6 H^+^. Despite the high energy barrier required for methanol generation, its formation still has certain advantages compared with other more complex high value-added products, especially in the design and optimization of catalysts. Researchers have explored a variety of catalytic materials, such as Ti-based, Cu-based, AgCu alloys, and certain transition metal-based catalysts, which help to enhance the hydrogenation capacity of *CO intermediates, thereby improving methanol selectivity.^209^ In recent years, researchers have made notable progress by adjusting the surface structure, electronic properties and reaction conditions of the catalyst, and the FE of methanol has exceeded 90%. The performance of PEC CO_2_RR for CH_3_OH is summarized in Table 5.

**Table 5:** Summary of the PEC CO_2_RR performance for CH_3_OH

Catalyst	Electrolyte	Light source	Product and performance	Ref.
CuO-graphene-ZnFe_2_O_4_–TiO_2_	0.04 M NaHCO_3_	UV light (*λ* = 254 nm)	FE = 44.8%	210
BiOBr	KHCO_3_	—	FE = 60.68% at 1.2 V *vs.* RHE	211
Macroporous conjugated polymer	0.10 M KHCO_3_	AM 1.5G	FE = 85% at 0 V *vs.* RHE	212
TNT/Cu_2_O/Au	0.1 M Na_2_SO_4_	300 W Xe lamp	FE_MeOH_ = 43% and FE_Methane_ = 27% at 0.2 V *vs.* Ag/AgCl	213
g-C_3_N_4_/ZnO	0.1 M KHCO_3_	UV LED light (365 nm, 4 W) of 80 mW cm^−2^	FE = 23% at −1 V *vs.* RHE	214
CNT/CoPc-NH_2_	0.1 M KHCO_3_	300 W Xe lamp	FE = 20% at −10.7 V *vs.* RHE	215
BiVO_4_	0.5 M NaHCO_3_	100 W LED	(MeOH) 22 and (HAc) 5.5 mmol cm^−2^ at −1.0 V *vs.* Ag/AgCl	216
CoPc/CN	[BMMIm]Br	300 W Xe lamp in the 350–780 nm range	(MeOH) 6465.9 and (EtOH) 218.6 mmol cm^−2^ at −1.2 V *vs.* RHE	101
Cu_2_O/PANI/SiPY	0.5 M CuSO_4_	Visible light (70 W)	FE = 57.66% at −1.3 V *vs.* SCE	72
Mo/CuGaS_2_/CdS/TiO_2_	0.1 M Na_2_SO_4_	AM 1.5G of 100 mW cm^−2^	FE = 65% at −0.7 V *vs.* RHE	217
Ti/TiO_2_/PDA/phosphorene	0.1 M Na_2_SO_4_	300 W Xe lamp	458 μmol L^−1^ at −0.8 V *vs.* Ag/AgCl	104
rGO-CuO/Cu	0.5 M Na_2_SO_4_	Xe lamp of 100 mW cm^−2^	3.3 g L^−1^ h^−1^ at −0.8 V *vs.* Ag/AgCl	218
Ag/α-Fe_2_O_3_	0.1 M KHCO_3_	AM 1.5G	FE = 51% at −0.5 V *vs.* RHE	209
Re doped CuO/TiO_2_ NTs	0.1 M NaHCO_3_	300 W Xe lamp	(MeOH) 19.9 and (EtOH) 7.5 mmol 5 h^−1^ at −0.4 V *vs.* RHE	63
TiO_2_-NT/GNR	0.1 M Na_2_SO_4_	150 W Xe lamp	FE_MeOH_ = 84.17% and FE_EtOH_ = 13.78% at 1.0 V *vs.* Ag/AgCl	219
CuFeO_2_/CuInS_2_	0.1 M CH_3_COONa-10 mM pyridine	Xe lamp of 100 mW cm^−2^	FE = 87% at −0.65 V *vs.* SCE	220
Ti/TiO_2_NT-Ru_3_(BTC)_2_	0.1 M Na_2_SO_4_	300 W Xe lamp	314 μmol L^−1^ at −0.5 V *vs.* Ag/AgCl	221
Au/α-Fe_2_O_3_/RGO	0.1 M KOH	A Xe lamp (cutoff range: 400–450 nm)	FE = 91% at −0.6 V *vs.* RHE	222
Cu/Cu_2_O–Cu(BDC) MOF	0.1 M Na_2_SO_4_	300 W Xe lamp	225 μmol L^−1^ h^−1^ at 0.1 V *vs.* RHE	223
Au@TNT	0.5 M KHCO_3_, 0.5 M Na_2_SO_4_	AM 1.5G	(MeOH) 2.5 and (EtOH) 2 mM h^−1^ at 1 V *vs.* Ag/AgCl	224
Cu	0.1 M NaHCO_3_	470 nm light source	FE = 73% at −0.4 V *vs.* NHE	225
rGO/Sn_3_O_4_/SnO_2_	0.5 M Na_2_SO_4_	300 W Xe lamp	FE = 45% at −0.3 V *vs.* Ag/AgCl	226
Ag–TiO_2_/RGO	1.0 M KOH	UV-vis light	FE = 60.5% at −0.7 V *vs.* RHE	227
Cu@Cu_2_O/TiO_2_/Ti_3_C_2_	0.1 M KHCO_3_	300 W Xe lamp	(MeOH) 1276.2 and (EtOH) 960.5 μMg^−1^ h^−1^ at −0.8 V *vs.* SCE	118

Early studies showed that TiO_2_, as a typical semiconductor material, can effectively promote the CO_2_RR due to its excellent photocatalytic properties, especially in catalyzing the conversion of CO_2_ to CH_2_OH.^24^ With advances in photocatalysis technology, TiO_2_ nanotubes (TNTs) have been developed as electrode substrate materials due to their unique structural advantages. The ordered nanoarray structure of TNTs has both a high specific surface area and unique nanoscale porosity characteristics, which not only greatly increases the density of active sites, but also promotes the separation efficiency of photogenerated electron–hole pairs by constructing directional charge transport channels, thereby systematically improving the catalytic performance. Further studies have shown that surface modification with transition metals or precious metal doping can effectively regulate the energy band structure of TNTs, and optimize the selectivity and kinetics of the CO_2_ reduction reaction path. A Ru_3_(BTC)_2_ metal–organic framework (MOF) film was constructed on the Ti/TiO_2_ nanotube substrate to develop a new photocathode system. The design cleverly combines the porous adsorption properties of the Ru based MOF with the photoresponse advantage of anatase type TiO_2_, showing enhanced photocurrent response characteristics under UV-visible light irradiation.^221^ The experimental data showed that under optimal reaction conditions, 314 μmol L^−1^ methanol could be generated by the photocatalytic reaction of the composite electrode for 3 h, with a yield 2.3 times higher than that of the traditional photocatalytic system, which confirmed the effectiveness of the interface engineering strategy. Zanoni's research group^213^ innovatively introduced a polydopamine (PDA) interface layer as an electron transport medium by constructing a heterogeneous structure of Cu_2_O–Au/TiO_2_ nanotubes. The results showed that the π-conjugated electron system of PDA can significantly promote the interfacial charge transfer, which enabled the cube-like catalyst to exhibit better performance than the octahedral catalyst, achieving the selective co-production of methanol (43%) and methane (27%). This finding provides an important experimental basis for the study of morphology–property correlation. The plasma silver–titanium dioxide/reduced graphene oxide (Ag–TiO_2_/RGO) nanohybrid system designed by Banat's team^227^ showed excellent performance. The material prepared by hydrothermal synthesis has clear structural characteristics: 4 nm silver nanoparticles and 7 nm TiO_2_ nanoparticles are uniformly loaded on the surface of micron-scale two-dimensional RGO nanosheets. The photochemical test showed that the photocathode achieved a current density of 23.5 mA cm^−2^ and a methanol yield of 85 μmol L^−1^ cm^−2^ at an initial potential of −0.7 V under UV-visible irradiation in a 1.0 M KOH electrolyte system. The quantum efficiency was 20% and FE was 60.5%. Otgonbayar *et al.*^210^ constructed a CuO–graphene–ZnFe_2_O_4_–TiO_2_ quaternary heterojunction that exhibited unique advantages. The results showed that the material achieved 44.08% FE in the buffer electrolyte system under ultraviolet irradiation, and maintained 42.2% FE in an electrolyte system with NaHCO_3_. The mechanism study showed that the gradient band arrangement built in the heterojunction effectively promotes carrier separation. Notably, the significant effect of proton concentration on the FE value reveals the critical role of electrolyte engineering in optimizing the reaction system.

**Fig. 14:**
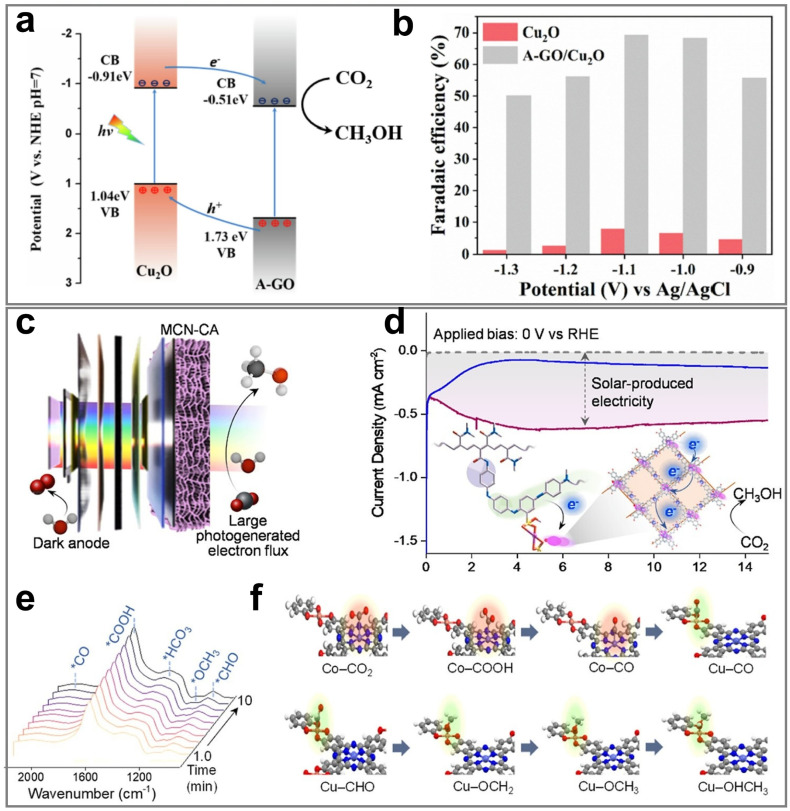
(a) A schematic diagram of the charge transfer mechanism of A-GO/Cu_2_O in the PEC CO_2_RR. (b) FE of CH_3_OH production. Reproduced with permission from ref. 228. Copyright 2021, Elsevier B.V. All rights reserved. (c) Schematic depiction of solar CH_3_OH production by the MCN photoelectrode with enhanced photogenerated electron flux. (d) Photocurrent density generated by solar irradiation of MCN-CA and MCN-C. (e) *In situ* FT-IR spectra of cobalt–phthalocyanine catalyst assembly. (f) Schematic illustration of the cooperative CO_2_-to-CH_3_OH conversion mechanism for cobalt–phthalocyanine catalyst assembly. Reproduced with permission from ref. 212. Copyright 2024, American Chemical Society.

The graphene oxide (GO) based catalytic system has shown unique advantages in the field of methanol synthesis. As shown in Fig. 14a and b, Liu's research team^228^ innovatively integrated adenine functionalized graphene oxide (A-GO) as an interface modification layer on the surface of the Cu_2_O photocathode, and constructed an A-GO/Cu_2_O composite catalytic system. The A-GO interface layer significantly optimized charge transport kinetics at the electrode interface through its abundant π–π conjugated network, thereby simultaneously improving the photoelectrochemical activity and cyclic stability of the system. The photocurrent density of the A-GO/Cu_2_O photocathode at −1.1 V *vs.* Ag/AgCl potential was 2.74 mA cm^−2^, which was 2.23 times higher than that of the bare Cu_2_O and GO/Cu_2_O systems, and 1.56 times higher than that of the bare Cu_2_O and GO/Cu_2_O systems, respectively. The mechanism study showed that the Lewis base site in the adenine molecule and the N atom in the pyrimidine ring could cooperatively regulate the adsorption energy barrier of CO_2_ intermediates, thus optimizing the PEC CO_2_RR path. The system achieved a 69.25% FE of methanol products in the continuous reaction, which confirmed the effectiveness of the interface engineering strategy of biomolecules and graphene oxide. In addition, Banat's team^222^ developed a PEC electrolyzer based on the Au/α-Fe_2_O_3_/RGO photocathode and Ru/RGO anode. This innovative design synchronized the bi-functional reaction of CO_2_ reduction at the cathode to methanol and the oxidation of furfural (FF) at the anode to produce 2-furanoic acid (2-FA) and 5-hydroxyfuranoic acid (5-HFA). The photocathode performance test showed that under visible light irradiation and −0.6 V bias, the system achieved a quantum efficiency of 21.5%, a stable methanol yield of 63 μmol L^−1^ h^−1^ cm^−2^, and a FE record of 91%. The simultaneous anodic reaction showed that the conversion rate of furfural was as high as 82%, and the yield of 2-FA and 5-HFA was 37% higher than that of conventional electrochemical system. This mechanism improves the overall energy efficiency of the system and provides a new paradigm for PEC system design.

A variety of organic catalytic systems also show significant potential in selectivity. Xiao's team^215^ successfully realized the efficient synthesis of methanol by constructing a micro-space environment using the CoPc-NH_2_ molecular catalyst. The silicon micropillar array structure significantly improved the catalyst loading and interface bonding strength while maintaining the light absorption efficiency by expanding the electrode surface area, and enriched the gaseous intermediates near the active site of the catalyst through the confinement effect. The introduction of superhydrophobic coating effectively inhibited the occurrence of side reactions, and realized the local enrichment of reaction intermediates through capillary action. After the single-electron reduction process of the molecular catalyst, the semiconductor–catalyst interface structure changed from an adaptive junction to an embedded junction, and the interface reconstruction provided sufficient thermodynamic driving force for the CO_2_ reduction reaction. The above synergistic effect enabled the system to achieve a 20% methanol FE during the CO_2_ electroreduction process, with a partial current density of 3.4 mA cm^−2^ which was 17 times higher than that of the planar silicon photoelectrode reference system. Inspired by the porous structure of photosynthetic organelles, Shan's research team^212^ developed a novel photoelectrode based on a single-pore macroporous conjugated polymer network (MCN) (Fig. 14c–f). The material achieved supramolecular assembly of photocatalytic components through rich functional groups, overcoming the geometric limitations of traditional inorganic materials. A stable interface was formed between MCN and the photocatalyst through a strong chemical bond network. The results showed that the Co-based catalyst and MCN composite system can produce highly reducing electrons under light conditions, and realize the CO_2_ to CH_3_OH conversion (conversion efficiency 70%), and the process can run for more than 100 h, with only a slight decline in activity. It is worth noting that when the electrode area was enlarged from 1 cm^2^ to 100 cm^2^, the system still maintained a stable photocurrent output of 0.25 A, and continued to generate CH_3_OH at 85% conversion efficiency, which confirmed the feasibility of large-scale application. In the field of organic–inorganic hybrid materials, Hocine's team^72^ found that the combination of polyaniline (PANI) and cuprous oxide (Cu_2_O) significantly improved the CO_2_ reduction performance of the PEC system. This enhancement effect was due to the enhancement of CO_2_ chemisorption capacity on the photocathode surface and the enhancement of photogenerated e^−^/h^+^ on the separation efficiency. The electrochemical test showed that the application of potential could significantly regulate the stability of photocurrent. When the Cu_2_O/PANI/SiPY heterojunction was used as the photocathode, a methanol FE of 57.66% was obtained at −1.2 V (*vs.* SCE) bias. Chidambaram's research group^214^ prepared a composite catalyst of g-C_3_N_4_ organic material supported by ZnO inorganic nanorods. At −1 V bias, the photocurrent density of the composite was 2.8 times higher than that of bare g-C_3_N_4_. The reduction efficiency of PEC CO_2_ to methanol was 2.86 times greater than that of the bare ZnO catalyst. The performance enhancement was mainly due to three factors: (1) the higher specific surface area provides abundant active sites; (2) increased carrier generation rate; (3) reduced e^−^/h^+^ pair recombination probability. Compared with a single component, g-C_3_N_4_/ZnO composites showed significant advantages in methanol yield.

Therefore, as a liquid C1 reduction product, methanol has been increasingly studied in recent years. Although the methanol generation process is relatively complex, it still has certain advantages compared with other high value-added products. In recent years, researchers have significantly improved the efficiency of methanol generation and FE by optimizing catalyst designs, such as the use of TiO_2_ nanotubes, Cu-based catalysts, and graphene oxide carriers. In addition, some organic catalysts and organo–inorganic hybrid materials also showed excellent catalytic activity in methanol generation. In particular, the precise regulation of the microspace environment may be a key factor in achieving selective methanol generation.

#### 3.1.4 CH_4_

CH_4_ is the only C_1_ gas product without oxygen. It can be seen that the formation process of methane requires not only the breaking of all C–O bonds, but also the formation of four new C–H bonds. Compared with the formation process of CO, the complexity is greatly increased. This process involves multiple electron and proton transfer steps, and usually 8 electrons and 8 protons are required to reduce CO_2_ to methane. Multiple intermediates will be formed during the reaction, and the reaction energy barrier is high, and the steps are more complicated. One of the main challenges in the selective generation of methane is how to promote the further hydrogenation of the *CO intermediate to form the *CHO intermediate and inhibit other competitive reactions, especially the desorption of CO.^23^ Therefore, at present, in the PEC CO_2_ reduction reaction, the regulation of the selective generation of methane is more difficult, and the corresponding research reports are relatively few. The performance of PEC CO_2_RR for CH_4_ is summarized in Table 6.

**Table 6:** Summary of the PEC CO_2_RR performance for CH_4_

Catalyst	Electrolyte	Light source	Product and performance	Ref.
Ag_*x*_Cu100_*x*_-Si MP	0.1 M KHCO_3_	AM 1.5G	FE_syngas_ = 16.7% and FE_methane_ = 9%	229
Si/TiO_2_/trzpOs	0.5 M Na_2_SO_4_	AM 1.5G of 100 mW cm^−2^	FE = 91.8% at 0 V *vs.* RHE	230
Cl–Cu_2_O/ZnO	0.1 M KHCO_3_	AM 1.5G	FE = 88.6% at −0.3 V *vs.* RHE	102
PbS/CsPbBr_3_-PNC	0.1 M TBAPF_6_	300 W Xe lamp	(CO) 2.94 and (methane) 0.36 μmol cm^−2^ h^−1^ at −0.6 V *vs.* Ag/AgCl	231
CuBDC/CF	0.1 M KHCO_3_	300 W Xe lamp	FE = 5% at −0.1 V *vs.* RHE	232
Cu–Ag	0.1 M KHCO_3_	100 mW cm^−2^	FE_CO_ = 79.8% at 1.0 V *vs.* RHE; FE = 59.3% at −1.4 V *vs.* RHE	233
Au/p-GaN	0.05 M KHCO_3_	Visible light	FE = 32.9% at −1.3 V *vs.* Ag/AgCl	234

**Fig. 15:**
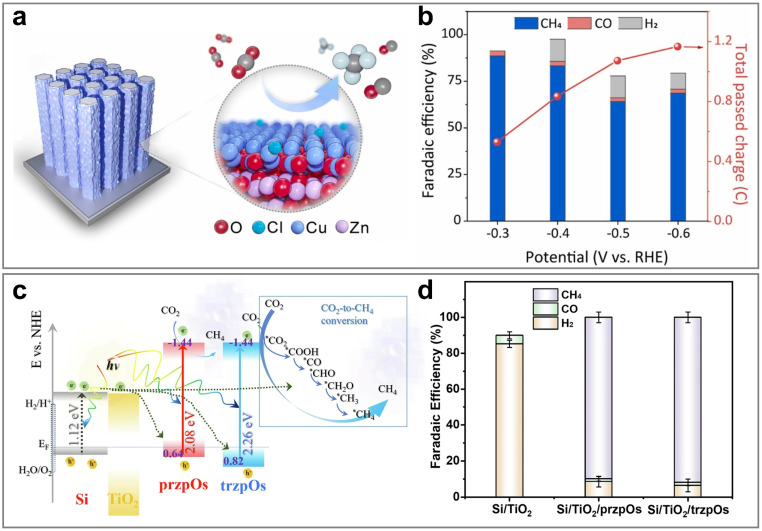
(a) Schematic illustration of the CCZO, (b) FE and total passed charge at different applied potentials for CCZO. Reproduced with permission from ref. 102. Copyright 2022, Elsevier B.V. All rights reserved. (c) Solar-driven CO_2_-to-CH_4_ conversion on the Si/TiO_2_ electrode in the presence of the [Os] complex. (d) FE of Si/TiO_2_, Si/TiO_2_/przpOs and Si/TiO_2_/trzpOs at 0 V *vs.* RHE. Reproduced with permission from ref. 230. Copyright 2024, Springer Nature.

In recent years, studies have shown that Cu-based catalysts are commonly used catalysts for the selective generation of CH_4_. As shown in Fig. 15a and b, Liu's team^102^ achieved a methane faradaic efficiency of 88.6% and catalytic stability for more than 5 hours by constructing a chlorine-element-modified Cu_2_O/ZnO heterostructure system. The study revealed that the Cl^−^ ions at the heterointerfaces effectively inhibited the photocorrosion phenomenon of Cu_2_O through passivation, and the stabilized Cu^+^ active sites significantly enhanced the hydrogenation process of the CO intermediate. Density functional theory calculations showed that compared with the energy barrier of 0.344 eV required for simple CO desorption, the CO intermediate in this heterostructure system tended to form the *CHO intermediate state through a low-energy barrier path of 0.220 eV. This characteristic explains the underlying reason why its methane selectivity is significantly better than the 60% benchmark value reported in earlier literature. In the study of bimetallic systems, the Cu–Ag composite catalyst also showed the advantage of regulating the selectivity of CH_4_.^229^ When the thickness of the Cu film decreased, its grain boundary density increased, and the Ag layer (3 nm) deposited on the surface showed a discrete island-like distribution feature.^233^ The uncoordinated Cu atoms at the grain boundaries are prone to spontaneous oxidation in an ambient atmosphere, and this interfacial oxidation phenomenon directly led to a decrease in the faradaic efficiency of CO and CH_4_. It is worth noting that when the thickness of the Cu layer reached more than 80 nm, the grain boundary oxidation process was effectively suppressed. At this time, the thin film catalyst showed catalytic performance comparable to that of the bulk Cu–Ag material. The optimized Cu (100 nm)–Ag (3 nm) composite film achieved a CO selectivity of 79.8% and a CH_4_ selectivity of 59.3% at −1.0 V *vs.* RHE and −1.4 V *vs.* RHE potentials, respectively, showing significant bifunctional catalytic characteristics. Further research has been extended to the photoelectrochemical system. By constructing a patterned array of Cu–Ag thin films on the surface of the p-type silicon photocathode and introducing a SiO_2_ passivation layer, the research team successfully developed a new type of composite photocatalyst. Under standard light intensity (100 mW cm^−2^), this structure simultaneously improved the selective production efficiency of CO and CH_4_, providing an innovative device design scheme for solar-driven CO_2_ conversion.

To significantly improve the selectivity of methane products in the photoelectrochemical carbon dioxide reduction reaction (PEC CRR), researchers have introduced molecular organic catalyst systems into this field. As shown in Fig. 15c and d, Jian's team^230^ innovatively used two osmium-based complexes—pyrazole-functionalized osmium complex (przpOs) and triazole-functionalized osmium complex (trzpOs)—as molecular catalysts for the selective reduction of CO_2_ to prepare CH_4_. Kinetic studies showed that przpOs and trzpOs exhibit high catalytic rate constants of 0.544 s^−1^ and 6.41 s^−1^, respectively, in the catalytic reduction of CO_2_, and this difference revealed the significant regulatory effect of the ligand structure on the reaction kinetics. Under AM1.5G standard solar irradiation, the optimally designed Si/TiO_2_/trzpOs composite photocathode shows excellent catalytic performance. The faradaic efficiency of its main product, CH_4_, reached 91.8%, and a photocurrent density of −14.11 mA cm^−2^ was achieved at 0.0 V *vs.* RHE potential. The nitrogen sites on the bipyrazole and triazole ligands effectively stabilized the adsorption configuration of the key intermediate *COOH through multiple coordination, promoting the reaction path toward deep hydrogenation to generate CH_4_, thereby endowing the system with ultra-high CO_2_-to-CH_4_ selectivity. It is worth noting that the comprehensive performance indicators of this system (including faradaic efficiency and current density) have reached the cutting-edge level among silicon-based photocathode materials for CO_2_ reduction, fully verifying the application potential of molecular catalysts in photoelectrochemical methane synthesis. In addition, Khezri's research group^235^ successfully achieved a methane faradaic efficiency of 74% by regulating the dopamine polymerization time to 15 hours to form a polymer coating layer and combining with the surface modification of copper nanoparticles, which further expanded the design idea of organic–inorganic hybrid catalysts in the field of CO_2_ reduction.

In PEC CO_2_RR, the selective production of CH_4_ is more challenging than the conversion of CO_2_ to CO because the generation of methane involves more complex multi-step reactions, including the breaking of the C–O bond and the formation of the C–H bond. Although Cu-based catalysts show certain advantages in the selective generation of methane, the problem of excessive generation of CO intermediates still needs to be overcome. Further modification of Cu-based catalysts (such as constructing bimetallic systems, heterostructures, and other modification methods) is still needed to significantly improve the faradaic efficiency of methane and improve the stability of the catalyst. In addition, organic catalysts also show great potential in improving the selectivity of methane. Therefore, in-depth studies on catalyst design and reaction mechanisms are needed, especially by regulating the surface structure of the catalyst and the generation path of intermediates, and it is expected that more efficient and selective methane generation can be achieved in the future.

### 3.2 C_2+_ products

In the process of PEC CO_2_RR, the controllable synthesis of C_2+_ products faces more significant technical bottlenecks compared to C_1_ products. The challenge stems from the complexity of the multistep reaction pathways and the dual effects of thermodynamic and kinetic co-constraints.^19^ On the one hand, in the CO_2_ reduction reaction, the generation of C_2+_ products often competes with side reactions such as the formation of CO, hydrogenation reactions, *etc.* Therefore, these side reactions need to be suppressed and the appropriate reaction pathways need to be promoted to improve the selectivity of C_2+_ products. Especially, the efficiency and selectivity of C–C coupling directly affect the yield of C_2+_ products. The intermediates generated during the reaction (such as *CH_2_ and C_2_H_4_O) have low stability and are prone to desorption or further decomposition into C_1_ products, making the selectivity difficult to control. On the other hand, the formation of C_2+_ products requires multiple consecutive PCET steps (usually requiring more than 8 electrons to participate) and involves high-energy barrier C–C coupling reactions (such as the dimerization of *CO to form the *OCCO intermediate), imposing higher demands on the design of the active sites of the catalyst and the efficiency of electron transfer.

#### 3.2.1 CH_3_COOH

CH_3_COOH is an important organic acid that is widely used in the fields of chemistry, food, medicine, and energy. As a basic C_2_-level liquid product, acetic acid serves not only a key raw material for the production of chemicals such as acetate esters, cellulose acetate, pharmaceuticals, and dyes, but also as a solvent and food additive. Its market price is much higher than that of C_1_ and some C_2_ products, being 3.9 times higher than that of HCOOH and 2.2 times higher than that of ethylene, respectively. Its formation involves a complex multi-step reduction and C–C coupling process, requiring an 8-electron and 8-proton transfer pathway. In recent years, studies on CH_3_COOH have been limited. Moreover, during the generation process, products such as HCOOH and CO are easily produced along with it, making it difficult to improve the selectivity. Moreover, CH_3_COOH can be naturally produced by microorganisms. The highest selectivity (about 80%) of FE_HAc_ at present is achieved by the combined action of photoelectrocatalysis and microorganisms.^107^ Integrating microorganisms with a photoelectrocatalytic system offers a promising approach for a CH_3_COOH generation. The performance of PEC CO_2_RR for CH_3_COOH is summarized in Table 7.

**Table 7:** Summary of the PEC CO_2_RR performance for CH_3_COOH

Catalyst	Electrolyte	Light source	Product and performance	Ref.
*S. ovata*/p-Si	Bacterial medium, 0.1 M glycerol in 1 M KOH	Red light (740 nm) of 20 mW cm^−2^	FE = 80% at 0 V *vs.* RHE	107
CO_3_O_4_/TiO_2_	0.5 M Na_2_SO_4_	300 W Xe lamp	(HAc) 7, (MeOH) 6.5 and (HCOOH) 5.2 mg L^−1^ cm^−1^ h^−1^	240
BiOI–PdCu	0.1 M KHCO_3_	420 nm LED lamp	(HCOOH) 0.14 μmol g^−1^ h^−1^ at −0.12 V *vs.* NHE; (HAc) 0.18 μmol g^−1^ h^−1^ at 0.11 V *vs.* NHE	239
Ag/Cu_2_O	0.1 M Na_2_SO_4_	455 nm LED lamp (100 W cm^−2^)	FE = 54% at −0.4 V *vs.* Ag/AgCl	241
CuO/g-C_3_N_4_-microorganisms	—	100 W iodine–tungsten lamp	5.1 g L^−1^ at −1.05 V *vs.* RHE	242
Cu_2_O/Ag	0.1 M KHCO_3_	AM 1.5G	FE = 47.7% at −0.7 V *vs.* RHE	238
α-Fe_2_O_3_/g-C_3_N_4_-microorganisms	—	100 W iodine–tungsten lamp	0.33 g per L per day at −0.9 V *vs.* Ag/AgCl	73
Zn–Cu_2_O	0.1 M KHCO_3_, 0.5 M Na_2_SO_4_	300 W Xe lamp of 200 mW cm^−2^	FE = 58.1% at −0.5 V *vs.* Ag/AgCl	237
TiO_2_/Cu_2_O	0.1 M KHCO_3_	300 W Xe lamp	20 μmol cm^−2^ at −0.4 V *vs.* RHE	243
Cu_2_O–TiO_2_	0.1 M KHCO_3_, 1 M NaOH	300 W Xe lamp	FE = 61.9%	236

**Fig. 16:**
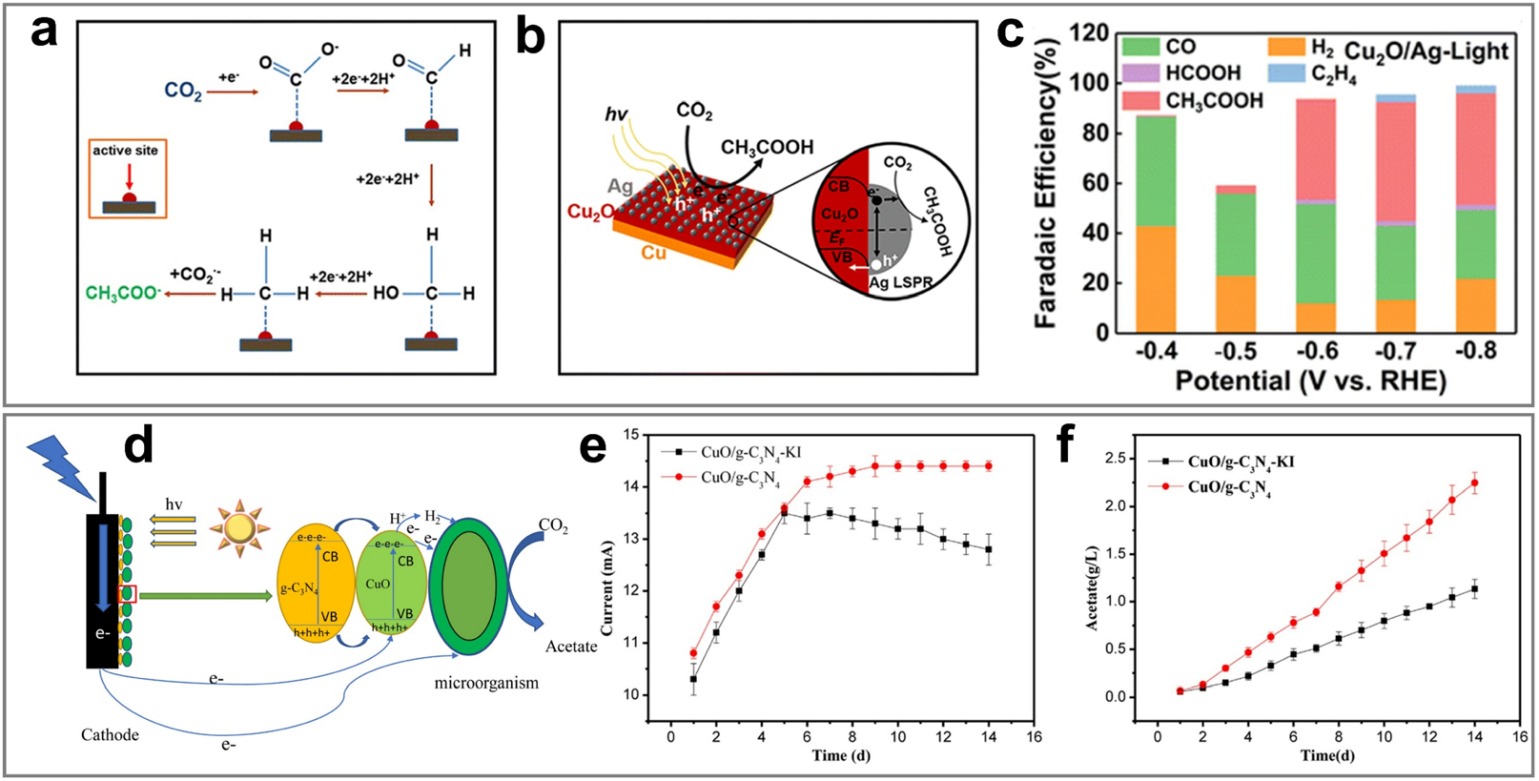
(a) Schematic diagram of reaction pathways. (b) Charge transport diagram of Cu_2_O/Ag for PEC CO_2_RR. (c) FE of products on the cathode under different applied potentials. Reproduced with permission from ref. 238. Copyright 2022, The Royal Society of Chemistry (d) Photocatalytic mechanism of MES. Comparison of (e) current density and (f) acetate yield. Reproduced with permission from ref. 242. Copyright 2021, published by Elsevier B.V.

Cu-based catalysts are a common choice in the catalysis of C_2+_ products. In the reduction reaction of Hac, Cu-based catalysts often exist in the form of metal oxides or alloys. Studies have shown that when applied to the reduction reaction of HAc, copper-based materials often present their catalytic activity in the form of metal oxides or alloys. Giusi *et al.*^236^ reported the successful construction of a Cu_2_O–TiO_2_ heterogeneous structure catalytic system through ultrasound-assisted coprecipitation. Experimental data showed that in the bare Cu_2_O/GDL electrode system, the production rates of formic acid and acetic acid were 31.8 and 80.6 μmol h^−1^ g^−1^, while the catalytic performance of the Cu_2_O–TiO_2_/GDL composite electrode was significantly improved, with the corresponding yields reaching 0.69 and 2.59 mmol h^−1^ g^−1^. Among them, the FE of acetic acid production was further increased to 61.9%. This performance enhancement can be attributed to the synergistic electrocatalytic effect at the Cu_2_O–TiO_2_ heterogeneous interface. This structure effectively promotes the formation of C–C bonds by optimizing the electron transfer path, providing a new approach for the sustainable synthesis of C_2+_ chemicals and fuels. Further studies showed that the coupling system of a Au-loaded nitrogen-doped TiO_2_ nanoarray photoanode and a Zn-doped Cu_2_O dark cathode realized the efficient conversion of carbon dioxide to acetic acid at a potential of 0.5 V (*vs.* Ag/AgCl), with a FE of 58.1% (carbon selectivity of 91.5%).^237^ The analysis showed that the Zn dopant improved the catalytic selectivity in the Cu_2_O lattice through a dual mechanism: on the one hand, it regulated the local electronic structure, and on the other hand, it modified the configuration of the active sites. The two synergistically promote the formation of the key intermediate *CH_2_/*CH_3_ and its C–C coupling process. At the same time, the efficient carrier separation ability of the nitrogen-doped TiO_2_-based photoanode provides sufficient electron supply for the multi-electron transfer CO_2_ reduction reaction. As shown in Fig. 16a–c, another innovative study used plasma metal (Ag) to modify the Cu_2_O nanowire array to construct a photoelectrochemical catalytic system with enhanced charge separation efficiency.^238^ Experimental results showed that the acetic acid production rate of the Cu_2_O/Ag composite photoelectrode reached 212.7 μmol cm^−2^ h^−1^ under a bias of 0.7 V (*vs.* RHE) under light conditions, and its FE was 47.7%, which was 4.8 times higher than that under dark conditions (44.4 μmol cm^−2^ h^−1^), confirming the key role of the plasma effect in strengthening the surface catalytic reaction. In the area of multi-metal composite catalyst research, the catalytic performances of three composite materials, BiOI–Pd, BiOI–Cu, and BiOI–PdCu, were systematically compared.^239^ Electrochemical tests showed that at a potential of −0.85 V (*vs.* RHE), the Faraday current density of the BiOI–PdCu composite electrode reached 3.15 mA cm^−2^, which was significantly better than that of the BiOI–Pd (2.06 mA cm^−2^) and BiOI–Cu (2.15 mA cm^−2^) systems. It is worth noting that this bimetallic composite catalyst showed a unique light response characteristic under visible light irradiation, and its main reduction products were formic acid and acetic acid, providing an important reference for the development of a photo-electrochemical catalytic system.

Combining the reaction device with a microbial system is an effective research strategy to achieve the synthesis of HAc. The research team successfully constructed a self-biased microbial photoelectrochemical cell (LMPC) based on the synergy of a gas diffusion photoelectrode cathode and a microbial anode.^240^ In this system, while achieving the oxidative degradation of anode pollutants, the process of reducing CO_2_ to liquid products at the photoelectrode cathode was simultaneously completed. Experimental data showed that after 10 hours of operation under light conditions, the production rate of acetic acid reached 70.4 ± 4.8 mg L^−1^ cm, which was 1.7 times higher than that of the unmodified photoelectrode cathode system. It is worth noting that this process was accompanied by the generation of a significant amount of methanol and formic acid by-products, and the selectivity of the acetic acid product was approximately 37%. It should be particularly pointed out that this technical approach does not directly utilize the acetic acid synthesis capability of microorganisms, but rather indirectly improves the system performance by enhancing the carrier separation efficiency through the microbial degradation effect. In the field of visible light-responsive photoelectrode microbial electrosynthesis (MES), recent studies have made significant progress. The Z-scheme heterojunction system constructed based on α-Fe_2_O_3_/g-C_3_N_4_ exhibits excellent photogenerated electron–hole separation characteristics.^73^ The unique energy band structure of α-Fe_2_O_3,_ characterized by a low Fermi level facilitated the combination of anode transferred electrons and photogenerated holes, thereby providing an additional electrochemical driving force for the improvement of MES performance. More notably, the introduction of α-Fe_2_O_3_ significantly improved the electron transfer efficiency at the electrode–microorganism interface. Experimental results showed that the acetic acid yield of this composite material reached 0.33 g per L per d, which is 3 times higher than that achieved with the traditional carbon felt cathode system. This finding provides a new idea for the design of MES photoelectrode cathodes. Further innovative research showed that the CuO/g-C_3_N_4_ photoelectric material can be directly integrated into the MES system employing a mixed culture biocatalyst (Fig. 16d–f).^242^ This composite material not only exhibited excellent light absorption characteristics, but also achieved an ideal level of electron–hole separation efficiency. Mechanism analysis revealed that CuO/g-C_3_N_4_ mainly enhances the electron supply of electroautotrophic microorganisms through the direct electron transfer mechanism (rather than the traditional hydrogen-mediated indirect pathway), while the photogenerated holes produce a synergistic enhancement effect by capturing anode electrons. This photoelectrode cathode significantly reduced the charge transfer resistance of the system, improved the electron transfer rate, and ultimately achieved a significant 2.6-fold increase in the acetic acid yield by optimizing the biocatalyst loading and regulating the microbial community structure of the cathode.

Therefore, for this product, acetic acid, the catalytic process using Cu-based catalysts is similar to the generation process of other C_2+_ products to achieve efficient C–C coupling. Although its production can be achieved, the reaction environment of Cu-based catalysts requires strict regulation, often accompanied by the generation of a large number of additional products, resulting in a low selectivity of Hac. However, this product is special as some microorganisms can directly achieve its production, and it can be considered to be combined with the photoelectric system to stimulate microbial production with electrons. Therefore, constructing a photoelectrode microbial electrosynthesis (MES) system is a potential method for the highly selective reduction of carbon dioxide to acetic acid.

#### 3.2.2 C_2_H_4_

Ethylene is the most common C_2_ gaseous product in the photoelectrocatalytic carbon dioxide reduction reaction system and its generation involves a concerted complex 12-electron–proton transfer process. In the initial stage of the reaction, the CO_2_ molecule undergoes a continuous two-electron and two-proton transfer process to gradually reduce and generate the key intermediate CO (CO_2_ → *COOH → *CO); subsequently, adjacent CO intermediates undergo surface migration and form high-energy *OCCO or *CO–COH intermediates through C–C coupling.^14^ This step is highly sensitive to the catalyst surface adsorption energy. Excessively strong chemical adsorption limits the migration of intermediates, while insufficient adsorption energy makes it difficult to maintain the stability of the coupling configuration; next, the OCCO intermediate undergoes a concerted continuous 10-electron-proton transfer process and is gradually converted into metastable intermediate products such as *CH_2_CH_2_O and *C_2_H_4_O through a multi-step hydrogenation/deoxygenation reaction pathway, finally releasing C_2_H_4_ through the deoxygenation step.^7^ This process imposes dual requirements on catalyst performance: it is necessary to precisely regulate the surface electronic structure to achieve the selectivity of C–C coupling, while suppressing the formation of ethane (C_2_H_6_) or C_1_ by-products caused by excessive hydrogenation. As an important target product in the field of PEC CO_2_RR, the synthesis of ethylene faces multiple challenges: the high activation energy barrier of the C–C coupling step, the competitive reaction pathways of the multi-electron transfer process, and the complexity of product selectivity regulation. Current research data show that under typical experimental conditions, the FE of the ethylene product rarely exceeds 70% in the system. The performance of PEC CO_2_RR for C_2_H_4_ is summarized in Table 8.

**Table 8:** Summary of the PEC CO_2_RR performance for C_2_H_4_

Catalyst	Electrolyte	Light source	Product and performance	Ref.
Cu-cluster/GaN	0.1 M KHCO_3_	AM 1.5G of 100 mW cm^−2^	FE = 61% at −0.74 V *vs.* RHE	22
Cu_2_O	0.1 M KHCO_3_	100 W Xe lamp	FE = 57% at −1.4 V *vs.* RHE	106
Cu–TiO_2_	1 M KOH	UV LED (365 nm)	FE = 46.6% at −1.8 V *vs.* Ag/AgCl	46
CuNP/SiNW	0.1 M KHCO_3_	AM 1.5G	FE = 25% at −0.5 V *vs.* RHE	244
Cu_2_O	0.1 M KHCO_3_	AM 1.5G of 100 mW cm^−2^	FE = 60% at −1.2 V *vs.* Fc^+^/Fc	2
Cu_2_Ta_4_O_11_	0.1 M KHCO_3_	AM 1.5G of 100 mW cm^−2^	FE = 13.9% at 0 V *vs.* RHE	245

**Fig. 17:**
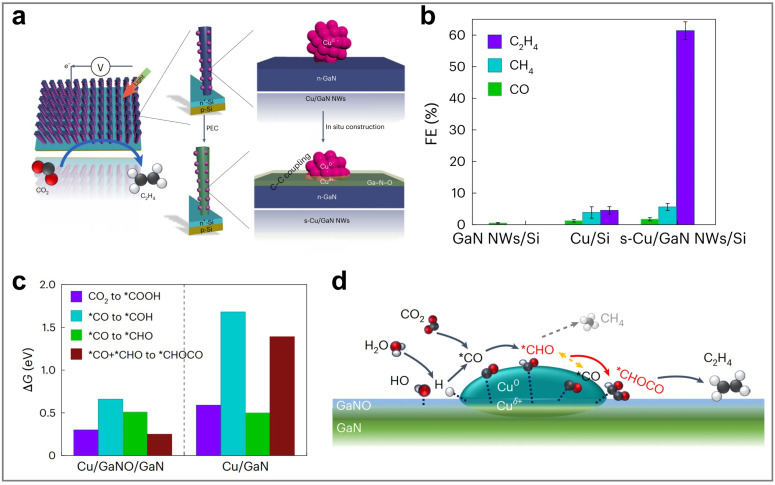
(a) Schematic diagram of the *in situ* construction of copper clusters and photocathodes. (b) FE of products at different photocathodes. (c) Energy barriers at different steps in the free energy diagram. (d) Possible reaction mechanism of CO_2_RR to C_2_H_4_. Reproduced with permission from ref. 22. Copyright 2024, Springer Nature.

In the research on photoelectrocatalysts for ethylene generation, copper-based materials have always been dominant. Roh *et al.*^244^ constructed a coupled system consisting of a silicon nanowire (SiNW) photocathode and a copper nanoparticle (CuNP) ensemble, which exhibited selectivity for CO_2_ conversion to C_2_H_4_ at −0.50 V *vs.* RHE, with a faradaic efficiency close to 25% and achieved a partial current density exceeding 2.5 mA cm^−2^. Notably, further stability tests showed that the CuNP/SiNW composite system could maintain stable CO_2_ reduction performance under constant potential illumination conditions for 50 hours. The above results experimentally confirmed the feasibility of the nanowire/Cu catalyst system as a modular platform, which could not only achieve efficient separation of photogenerated carriers but also promote the complex reaction pathways of C_2_H_4_ products through interface regulation. Particularly, copper oxides, where active sites formed by low-valence copper can efficiently promote C–C coupling, may be one of the important factors promoting ethylene formation. Liu *et al.*^2^ proposed a charge separation strategy based on the Ag co-catalyst, by constructing a Z-scheme heterojunction to accelerate photogenerated electron transfer and selectively extract holes, thereby effectively suppressing the self-reduction of Cu_2_O under light conditions and hole-induced oxidative degradation. The optimized photocathode showed a stable CO_2_ reduction photocurrent response, with its ethylene faradaic efficiency increased to about 60%, and no significant attenuation after continuous operation for several hours, while the bare Cu_2_O electrode experienced significant performance attenuation within a few minutes. Recent breakthroughs in interface engineering strategies have further promoted the development of copper-based catalysts for ethylene generation. As shown in Fig. 17, Zhang *et al.*^22^ developed an *in situ* interface coupling architecture of low – coordinated copper cluster catalysts and GaN nanowire photocathodes, achieving a synergistic improvement with 61% faradaic efficiency for ethylene and 14.2 mA cm^−2^ partial current density, with system stability extended to 116 hours. The researchers revealed the dynamic self-optimization mechanism of the Ga–N–O interface: this interface not only stabilized the active cuprous oxide species on the copper cluster surface (as an efficient site for C–C coupling) but also regulated the CO hydrogenation process through the hydrogen spillover effect of GaN, thereby guiding the specific coupling pathway of *CHO intermediates.

Cu-based catalysts play an important role in ethylene generation, especially in improving selectivity by regulating the catalyst surface structure and promoting C–C coupling reactions. In recent years, researchers have made some progress through innovative catalyst design, such as the combination of Cu nanoparticles and silicon nanowires, low-coordinated copper cluster catalysts, *etc.*, and some studies have achieved a faradaic efficiency as high as 61% and made significant breakthroughs in stability. However, there are still very few related studies, and future research should continue to focus on the stability and selectivity of catalysts.

#### 3.2.3 C_2_H_5_OH

C_2_H_5_OH is a high value-added liquid C_2_ product in the PEC CO_2_RR. Its formation mechanism requires a 12-electron/12-proton transfer process, the same as that of ethylene, but it faces the challenge of regulating the *CHO/*COH intermediates. Although ethanol shows advantages such as high energy density, ease of separation and storage for energy storage and transportation applications, which drives research breakthroughs, its selective generation is still limited by the sharing of active sites with competitive products such as methanol and by kinetic control problems.^246^ Recently, the selective generation of ethanol has received more research attention compared to other C_2_ products, and the FE in a few research studies has reached 90%, or even close to100%. The performance of PEC CO_2_RR for C_2_H_5_OH is summarized in Table 9.

**Table 9:** Summary of the PEC CO_2_RR performance for C_2_H_5_OH

Catalyst	Electrolyte	Light source	Product and performance	Ref.
Cu_*x*_S-ReS_2_/TiO_2_NTs	0.1 M KHCO_3_	250 W Xe lamp of 200 mW cm^−2^	6 μmol cm^−2^ h^−1^ at −0.8 V *vs.* RHE	247
FeS_2_/TiO_2_	0.1 M NaHCO_3_	300 W Xe lamp	1170 μmol L^−1^ cm^−2^ at −0.7 V *vs.* RHE	248
C@SiC	0.5 M KHCO_3_	AM 1.5G of 100 mW cm^−2^	FE = 87.8%	249
BiFeO_3_	0.5 M KHCO_3_	300 W Xe lamp	FE = 23.2% at −0.7 V *vs.* RHE	250
ZnO/Cu_2_O/Cu	0.1 M NaSO_4_, 0.1 M KHCO_3_	365 nm UVA-LED and 430 nm vis-LED	FE_EtOH_ = 16% and FE_MeOH_ = 14% at 0.5 V *vs.* RHE	79
B-gC_3_N_4_/ZIF-67	0.1 M NaHCO_3_	Visible light	32.1096 μmol cm^−2^ h^−1^ at −1 V *vs.* NHE	251
CuFeO_2_/CuO	H_2_O-TEOA (9 : 1)	500 W Xe lamp of 100 mW cm^−2^	FE = 66.73% at −0.6 V *vs.* Ag/AgCl	252
CuS/MgFe_2_O_4_/Graphite	0.1 M KHPO_4_	20 W LED lamp, visible light	FE = 35%	253
GO/SiC	0.5 M KHCO_3_	300 W Xe lamp	FE = 99% at −0.05 V *vs.* Ag/AgCl	254
Cu_2_O/TiO_2_	[Emim]BF_4_	AM 1.5G	FE = 82.7% at −0.9 V *vs.* RHE	103
Cu_2_ZnSnS_4_/TiO_2_NTs	0.1 M NaHCO_3_	250 W Xe lamp	7.0 mmol cm^−2^ −0.6 V *vs.* RHE	255
Cu_*x*_O/GO-Cu-MOF	0.1 M KHCO_3_–Na_2_SO_4_ (4 : 1)	AM 1.5G	FE = 43% at −0.5 V *vs.* Ag/AgCl	256
Si/ZnO/Cu_2_O	0.1 M KHCO_3_	Simulated sunlight	FE = 60% at −0 V *vs.* RHE	257
Zn-TPY-TTF CPG	0.2 M Na_2_SO_4_, 0.5 M KHCO_3_	AM 1.5G of 100 mW cm^−2^	FE = 13.9% at 0 V *vs.* RHE	258
Carbazole-BODIPY	0.1 M TBAPF_6_-MeCN	50 W halogen lamp	FE = 34.79% at −1.15 V *vs.* NHE	259
Ag/Cu_2_O/g-C_3_N_4_	0.5 M KHCO_3_	300 W Xe lamp	FE = 44.0% at −1.0 V *vs.* RHE	260
CuO–MoO_3_/TiO_2_NTs	1 M NaHCO_3_	300 W Xe lamp of 200 mW cm^−2^	FE = 89% at −0.5 V *vs.* SCE	261

It is worth mentioning that the TiO_2_ photocatalyst not only has the catalytic ability to reduce carbon dioxide for methanol preparation, but also shows significant application potential in the field of ethanol synthesis. The research team successfully constructed the FeS_2_/TiO_2_ p–n heterojunction photoelectrode system by using the electrochemical anode oxidation–electrodeposition coupled sulfidation process.^248^ By systematically exploring the effects of the FeS_2_ loading amount and the applied bias voltage on the photoelectrochemical performance, the experiment found that the photocurrent polarity inversion phenomenon is significantly correlated with the FeS_2_ loading amount, and the essence of this phenomenon lies in the dynamic evolution of the energy band structure at the semiconductor/electrolyte interface. Through surface site analysis, it is confirmed that the active center for ethanol generation is localized on the surface of TiO_2_ nanotubes rather than at the FeS_2_ interface. The introduction of FeS_2_ not only realizes the expansion of the visible light response range, but also, more importantly, forms a p–n heterojunction structure with TiO_2_ to optimize the space charge layer. Under the synergy of −0.7 V bias voltage and ultraviolet-visible light (UV-vis), the FeS_2_/TiO_2_ sample with an electrodeposition time optimized to 15 minutes showed the optimal ethanol yield, reaching 1170 μmol L^−1^ cm^−2^ after a reaction time of 3.5 hours. Compared with pure TiO_2_, the ethanol yield of the FeS_2_/TiO_2_ system was significantly improved due to the synergistic effect of the optimized loading amount, enhanced UV-vis light capture ability, and efficient separation–migration of carriers at the p–n junction interface. Similarly, the TNTs also showed application potential in the field of ethanol synthesis. The Cu_2_ZnSnS_4_/TiO_2_NTs composite electrode was prepared by the pulse electrodeposition combined with the high temperature sulfidation process.^255^ After parameter optimization, the light response current density of this material reached 7 mA cm^−2^, and the ethanol yield remained stable at 7.0 mmol cm^−2^ during a 5 h photoelectrocatalytic reaction. The characterization analysis showed that no new phase is generated during the catalytic process, and the electrode still maintained a stable output performance at 33.2% of the initial photocurrent density after the reaction.

**Fig. 18:**
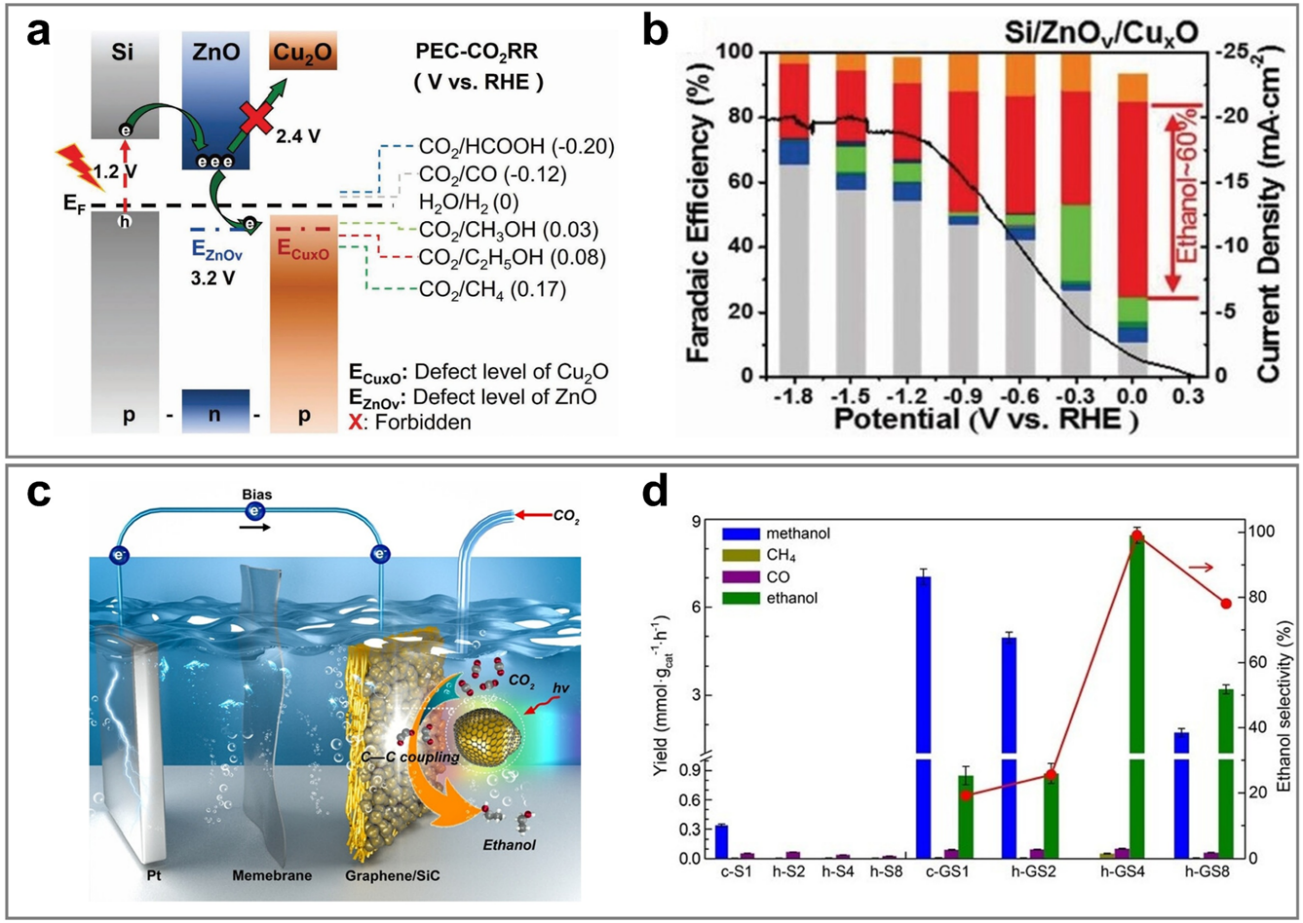
(a) Schematic illustration of the designed defect nanomaterial structure for selective PEC CO_2_RR to ethanol. (b) FE of ethanol. Reproduced with permission from ref. 257. Copyright 2023, Wiley-VCH GmbH (c) Mechanistic demonstration of the PEC CO_2_RR. (d) Comparison of PEC CO_2_RR properties of different samples. Reproduced with permission from ref. 254. Copyright 2023, Wiley-VCH GmbH.

Cu-based catalysts are also a major class of materials for producing ethanol. As shown in Fig. 18a and b, Kan *et al.*^257^ designed and constructed a Si/ZnO/Cu_2_O p–n–p heterojunction potential well structure with an electron tunnelling effect to achieve the selective photoelectrochemical CO_2_ reduction to ethanol. This heterojunction was formed by the controllable growth of n-type ZnO nanosheets between the defect-rich p-type Cu_2_O nanoparticles and the nanoporous p-type Si substrate. Driven by the built – in potential of approximately 0.6 V, the photogenerated electrons could be captured and enriched in the *n*-ZnO layer under low applied bias conditions, and then directionally migrated to the defect energy level of Cu_2_O through the tunneling effect. When the Si/ZnO/Cu_2_O photoelectrode was used for the photoelectrochemical CO_2_ reduction in the aqueous phase system under simulated sunlight irradiation, its onset potential relative to the reversible hydrogen electrode was positively shifted to 0.2 V. Due to the confined electron energy level distribution characteristics at the heterojunction interface, the product selectivity significantly shifted from the traditional CO or formate to ethanol, and the FE of ethanol exceeded 60% at 0 V *vs.* RHE. Regarding the photocorrosion problem of Cu-based catalysts, Lu *et al.*^252^ developed a 0D/1D CuFeO_2_/CuO nanowire heterojunction array with a high specific surface area. This heterojunction exhibited excellent photoelectrochemical CO_2_ reduction activity at −0.6 V *vs.* Ag/AgCl, and the FE of ethanol could reach up to 66.73%. The analysis showed that the built-in electric field at the heterojunction interface promoted the directional migration of photogenerated holes from the valence band of CuO to the valence band of CuFeO_2_, while the photogenerated electrons transferred from the conduction band of CuFeO_2_ to the conduction band of CuO. This synergistic mechanism significantly improves the efficiency of the highly selective reduction of CO_2_ to ethanol. Leng *et al.*^79^ constructed a ZnO/Cu_2_O/Cu composite photocathode by wet chemical oxidation combined with the hydrothermal method. The experimental results showed that at −0.5 V *vs.* RHE bias, the yields of CH_3_OH and C_2_H_5_OH were 10.0 μmol L^−1^ and 5.6 μmol L^−1^, respectively.

In addition to copper-based materials, recent studies have shown that some non-copper-based materials also have the potential to catalyse the reduction of CO_2_ to prepare ethanol. The spin polarization effect of the BiFeO_3_ catalyst improved the electron migration and utilization efficiency, successfully achieving the highly selective catalytic preparation of CO_2_ to ethanol.^250^ In the photoelectrocatalytic system, this material showed an excellent ethanol yield (7.05 μmol cm^−2^ h^−1^), and under a – 0.7 V *vs.* RHE bias voltage, the ethanol FE reached 23.2%. The characterization results showed that the external electric field induces the formation of a multi-electric field coupling effect in BiFeO_3_, effectively enhancing the carrier separation efficiency and optimizing the surface reaction path, thereby significantly improving the CO_2_ reduction performance. Feng *et al.*^249^ developed a carbon@silicon carbide (C@SiC) composite catalyst, which showed excellent CO_2_ photoelectroreduction performance under simulated solar radiation conditions, with a CO_2_ conversion rate of 487 μmol g_cat_^−1^ h^−1^ and an ethanol selectivity of up to 87.8%. The study confirmed that the optimal ratio of sp^2^/sp^3^ hybridized carbon in the carbon layer not only significantly promoted the efficient transfer of photogenerated electrons from SiC to the carbon layer, but also optimized the C–C coupling kinetics of the key intermediates, ultimately achieving the efficient conversion of CO_2_ to ethanol. As shown in Fig. 18c and d, Feng *et al.*^254^ carried out the photoelectrochemical reduction of CO_2_ on the graphene/silicon carbide (GO/SiC) catalyst. The FE of ethanol was >99%, and the CO_2_ conversion rate reached as high as 17.1 mmol g^−1^ h^−1^. Experimental and theoretical studies showed that the optimal interface layer helped the photogenerated electrons transfer from the SiC substrate to the monolayer graphene coating, which was beneficial for the efficient conversion of CO_2_ to C_2_H_5_OH.

It can be seen that TiO_2_ materials play a special role in the generation of alcohol materials; however achieving high selectivity for ethanol still needs further exploration. Cu-based catalysts also occupy an important position in the generation of ethanol, especially through the design of heterojunctions and improvement of the electron transfer efficiency to improve product selectivity. Non-copper materials such as BiFeO_3_ and C@SiC catalysts also show good ethanol generation ability and have high selectivity. In the future, it is necessary to further optimize and regulate the structure of TiO_2_ and Cu-based materials to reduce the generation of by – products, and at the same time, it is necessary to continuously explore new materials with high selectivity for ethanol production.

#### 3.2.4 Others

The C_2+_ products that have been studied extensively are ethylene, ethanol, and acetic acid. There are few reports on other C_2_, C_3_, and even C_4_ products, or they are expressed in terms of the overall FE of C_2+_. Therefore, this part of the content will describe the overall C_2+_ products that are not described in detail. These products involve a multi-electron proton transfer process involving more than 12 electrons and 12 protons. In addition, for C_3+_ and other products, multiple carbon–carbon couplings are required, involving higher reaction energy barriers, thus greatly increasing the difficulty of product generation. Therefore, the generation of these products is very limited, and further in-depth research is still needed.

In order to generate oxalic acid, Bergamini *et al.*^262^ employed a CsPbBr_3_/graphite composite system to achieve the directional synthesis of oxalic acid, obtaining an oxalic acid FE of 43.9% at a potential of −0.8 V *vs.* Ag/AgCl. Pawar *et al.*^263^ proposed the construction of an N-heterocyclic polymer coordination activation system to regulate the thermodynamic stability and reaction path of CO_2_ molecules. Studies have shown that by precisely selecting electrode materials and applying bias voltage parameters, the kinetics and thermodynamics of the photoelectrochemical CO_2_ reduction reaction could be doubly regulated. A solar-driven photoelectrocatalytic system based on a multilayer Cu/rGO/PVP/Nafion hybrid cathode achieved the selective generation of formaldehyde and acetaldehyde; however, the product distribution analysis showed the existence of significant side reactions, and the overall selectivity toward C_2_ products remained low. In addition, other C_2_ products were also generated, and the FE was usually expressed as the overall efficiency of C_2_. The CuN_*x*_/CuO heterojunction photoelectrode achieved a photocurrent density of −1.0 mA cm^−2^ at 0.2 V *vs.* RHE, which is 2.5 times higher than that of the pure CuO system.^264^ The total FE of this composite system for C_2_ products reached 15.2% at the same potential. Density functional theory calculations confirmed that the asymmetric d–p orbital hybrid Cu–N sites anchored on the CuO substrate can significantly reduce the C–C coupling free energy (Δ*G* = 0.86 eV), and this effect arises from the optimized regulation of the adsorption strength of *OCCO and *COCH_2_ intermediates. In addition, the local charge redistribution characteristics of the Cu–N sites enhance the electrical conductivity of the system, thereby improving the photogenerated electron transfer efficiency and photocurrent density. Cao *et al.*^265^ developed a NiMoO_4_/ZnO-3 heterojunction composite photoelectrode to achieve a synthesis rate of oxygen-containing compounds of 29.2 mmol cm^−2^ h^−1^, with a C2 product selectivity reaching 72.6%. The photoelectrochemical conduction rate was 3 times higher than that of a single photo/electrocatalytic system, indicating that the photo-electric synergy significantly enhances the reaction kinetics. Mechanism studies showed that the Ni–Mo dual active centers achieved the efficient and selective synthesis of C_2_ products by reducing the C–C coupling energy barrier. Wan *et al.*^68^ constructed a silicon nanowire array-supported copper-coordinated covalent triazine framework (Si@CuCTF_6_) heterostructure, which showed an ultra-high selectivity of 95.6% for multi-carbon products and an apparent quantum efficiency of 0.89% for carbon-based products. Synchronous radiation characterization confirmed that the active site stemmed from the synergistic effect of the coordination structure of the triazine ring nitrogen atom and the bipyridine unit Cu–N, and its ordered porous framework structure was conducive to the mass transfer of reactants and the stability of intermediates. Zhuang *et al.*^266^ reported a NiCO_2_O_4_@h-BN-6 organic–inorganic heterojunction system, which achieved 100% selective synthesis of C2 products for the first time. The detection of N–H species confirms that the nitrogen active site can efficiently adsorb/activate the proton–electron pair to generate highly active hydrogen atoms. Verification experiments showed that this material will oxidize the intermediate to acetic acid, with the boron atom existing as a hole in the semiconductor.

In particular, metal sulfide composite materials show significant potential in the catalytic synthesis of C_2_ products. Liu *et al.*^76^ successfully constructed a CdS/VS-MoS_2_ hollow heterojunction structure with sulfur vacancy (VS) enrichment characteristics. Benefiting from the improved mass transfer efficiency of the reaction medium in the confined cavity structure, the FE of the C2 product on its inner surface reached 67.0%. It was confirmed that the efficient capture of the H intermediate by surface S atoms could promote the kinetics of CO_2_ reduction. Cao *et al.*^93^ synthesized a porous Cu_2_S/MoS_2_-VS octahedral semiconductor heterostructure by using the MOF template method and revealed its topological confinement effect and photothermal synergy mechanism. Experiments showed that the introduction of sulfur vacancies significantly enhances the chemical adsorption capacity of CO_2_, and the formation of the heterointerfaces increases the charge transfer rate by 3.2 times. Based on DFT calculations, VS could reduce the activation energy for the formation of key intermediates. Liu *et al.*^92^ designed and prepared a series of CdIn_2_S_4_–N/C biomimetic catalytic systems by loading CdIn_2_S_4_ on the surface of nitrogen-doped carbon spheres to construct a plant cell-like structure for application in the photoelectrothermal synergistic catalysis of CO_2_. The hierarchical porous structure of the nitrogen-doped carbon matrix and the LSPR effect jointly endow the catalyst with excellent CO_2_ reduction performance. Among them, the CdIn_2_S_4_–N/C2-800 optimized sample showed excellent catalytic performance, with a carbon-based product generation rate of 168.5 μM h^−1^ cm^−2^, and the electron selectivity for C_2_ products reached 66.9%. Theoretical calculations showed that pyridinic and pyrrolic nitrogen doping sites could reduce the energy barrier of the rate-determining step of CO_2_ reduction. Two key intermediates, =C=O and OC–COH, were proposed based on this to construct the C_2_ product generation path based on the carbene (=C=O) coupling mechanism.

In addition to C_2+_ products, some three-carbon (C_3_) and higher carbon-containing compounds have been reported successively. Guo *et al.*^267^ developed a gallium-doped cuprous oxide catalyst (Ga/Cu_2_O), which showed excellent C_2+_ product selectivity in the CO_2_ electroreduction reaction. At a potential of −1.8 V (*vs.* RHE), the total FE for C_2+_ products reached 20%, with ethanol (CH_3_CH_2_OH) and propanol (CH_3_CH_2_CH_2_OH) contributing 6.5% and 6.64%, respectively. Experimental characterization confirmed that Ga doping induces the generation of an oxygen vacancy concentration gradient in Cu_2_O through the lattice substitution mechanism, promoting the rapid separation of photogenerated carriers, and at the same time strengthening the adsorption and activation process of CO_2_ molecules through electronic structure modulation. Compared with undoped Cu_2_O, the coverage of the *CO intermediate on the surface of Ga/Cu_2_O is significantly increased, and the C–C coupling energy barrier is reduced, which clarified the mechanism of the enhanced selectivity of C_2+_ products from a kinetic perspective. In addition, the p–n heterojunction Si@WO_3_-NS photocatalyst showed an apparent quantum efficiency of 0.49%, which is nearly 25 times higher than that of pure silicon nanowires (0.02% AQE), with a C_2+_ product selectivity of 62.7%.^268^ This performance improvement is attributed to the hierarchical structural design of the biomimetic plant thylakoid, which effectively optimizes the spatial transmission path of photogenerated carriers. Ponnamma's team^269^ further used a high-sensitivity detection technology to identify long-chain hydrocarbons such as octane (C_8_H_18_) and heptane (C_7_H_16_) in the products. In a transition metal (Fe, Co and Ni) doped TiO_2_ system prepared by the sol–gel method, the bandgap regulation effect of Fe–TiO_2_ is the most significant, and its bandgap value increases in the order of Fe–TiO_2_ < Co–TiO_2_ < Ni–TiO_2_. The gas chromatography characterization results of the reduction products confirmed the generation of hydrocarbons, and Fe–TiO_2_ showed the highest product abundance due to its optimal bandgap characteristics. It should be particularly noted that the quantitative analysis of multi-carbon gaseous products by gas chromatography requires high-sensitivity detection conditions or an extended retention time, and technical limitations often hinder actual detection.

Therefore, compared with C_2_ products such as ethylene, ethanol, and acetic acid, the generation of other products (such as ethane, propionic acid, propylene, *etc.*) faces significant challenges, and the difficulty mainly stems from the steep increase in the thermodynamic energy barrier and the complexity of the kinetic path: thermodynamically, more electron and proton migrations are required (for example, C_3_ products often require > 12 electrons and 12 protons), and kinetically, it involves the coordinated regulation of multiple steps of C–C coupling, proton migration, and intermediate stabilization. This imposes more stringent requirements on the design of the active sites of the catalyst (such as multi-metal synergy or confined microenvironments). It is worth mentioning that the ability of metal sulfide composite materials to reduce CO_2_ to C_2_ products is not limited to photoelectrocatalysis, and there are also a large number of reports in the field of photocatalysis. Thus, metal sulfide composite materials deserve attention. In addition, the precise detection of multi-carbon products also limits the accurate assessment of selectivity.

## 4 Reaction durability

Although considerable attention has been paid to activity and selectivity, the stability of product selectivity, which is often overlooked, is equally crucial for industrial applications.^123,161,270^ Catalyst failure can result in increased overpotential, reduced current, or diminished selectivity for the target product. A decline in catalyst activity or selectivity during operation not only lowers energy utilization efficiency, but also leads to the formation of by-products, increases the cost of separation and purification, and may even compromise the economic viability of the process. Therefore, enhancing both the selectivity and stability of products is essential for realizing the practical application of PEC CO_2_RR.

Compared with the stable operation of electrocatalytic CO_2_ reduction (EC CO_2_RR), which can last from tens to hundreds of hours, the actual stable operation time of PEC CO_2_RR is often much shorter, typically ranging from several hours to just over ten hours.^201,271^ Significant differences in stability exist between EC CO_2_RR and PEC CO_2_RR, primarily due to differences in energy input methods and material systems. Electrocatalytic CO_2_reduction relies solely on an applied voltage to drive the reaction and does not involve strong light irradiation. As a result, its stability issues are mainly related to catalyst degradation in the electrochemical environment, such as metal ion dissolution, surface reconstruction, electrode passivation, and corrosion of materials by electrolytes. In contrast, PEC CO_2_RR not only faces the same electrochemical stability challenges as EC CO_2_RR, but also contends with additional degradation mechanisms induced by light exposure. Semiconductor photoabsorbers are typically incorporated into PEC CO_2_RR systems. These materials are susceptible to photocorrosion or photo-induced reconstruction under intense illumination, leading to structural damage and performance degradation. Additionally, the accumulation of photogenerated carriers can trigger side reactions or accelerate material degradation, while chemical instability at heterojunctions or interfaces can further compromise the overall stability of the system. Due to the more complex architecture of PEC CO_2_RR devices, which require the coordinated operation of both the catalyst and the light absorber, failure in any single component can significantly impact overall performance and increase the difficulty of maintenance and repair. As a result, the stability of PEC CO_2_RR is generally lower than that of EC CO_2_RR, and its actual operational lifetime is often shorter. In general, the failure modes of PEC CO_2_RR are mainly attributed to photocorrosion, catalyst leaching, and structural collapse. Improving the stability of PEC CO_2_RR requires not only attention to the catalyst itself, but also enhanced protection and optimization of the light absorber and interface structures. This can be achieved through a range of collaborative strategies, such as introducing protective layers, accelerating carrier separation, developing semiconductor materials with improved resistance to photocorrosion, and refining reactor design, with the goal of achieving long-term and efficient CO_2_ reduction.

**Fig. 19:**
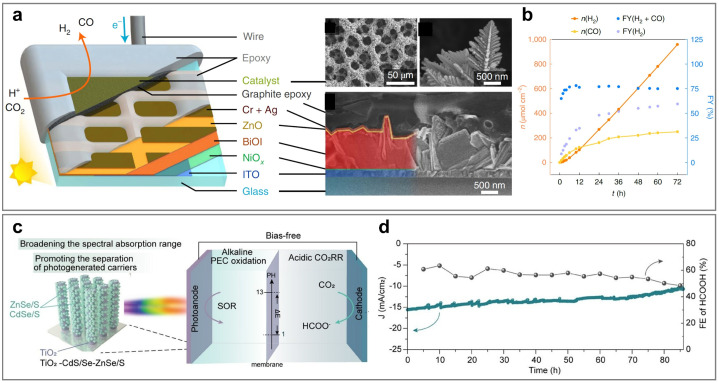
(a) Schematic depiction of the BiOI photocathode with its components. (b) The stability test. Reproduced with permission from ref. 167. Copyright 2022, Springer Nature. (c) Schematic diagram of solar-driven bias-free PEC-CO_2_RR system for formic acid production. (d) The stability test. Reproduced with permission from ref. 201. Copyright 2025, Wiley-VCH GmbH.

Introducing a protective layer into PEC CO_2_RR systems is an effective strategy to enhance the stability of the catalyst. Protective layers are typically applied to the surfaces of semiconductors or catalysts to prevent photocorrosion and chemical degradation of materials under intense illumination and electrochemical conditions. Moreover, a well-designed protective layer can facilitate efficient charge transfer and suppress interfacial carrier recombination, thereby maintaining high catalytic activity and product selectivity. Common protective layer materials include metal oxides (such as TiO_2_ and Al_2_O_3_), carbon materials, and conductive polymers.^243^ These materials not only exhibit excellent chemical stability, but also enable precise control over the catalytic interface by tuning their thickness and structure. For example, Bergamini *et al.*^262^ sputtered Pt or C onto CsPbBr_3_ to fabricate a hybrid photoelectrode with a uniform protective layer of consistent thickness. Owing to the presence of Pt and C layers, the instability of perovskite in aqueous environments was mitigated, and the resulting hybrid electrode retained its ability to absorb visible light. Prabhakar *et al.*^161^ also synthesized CIGS films by vulcanizing Cu–In–Ga metal laminates through sputtering. Their analysis suggests that the stability of CO_2_ reduction may be attributed to a self-protection effect, formed *in situ* by the oxides and hydroxides of Ga and In during operation. In addition to sputtering, oxide coatings represent another promising approach. For example, Andrei *et al.*^167^ extended the operational stability of their device to an impressive 72 h by integrating a BiOI photocatalyst into a robust oxide-based structure sealed with graphite paste (Fig. 19a). Xu *et al.*^272^ coated the organic compound pyridine as a co-catalyst onto Cu_2_O, which enhanced the selectivity of the Cu_2_O surface for CO_2_ reduction while maintaining the stability of the Cu_2_O (110) surface. Protective layers are thus of great significance for stabilizing precious metals and improving catalytic performance. Ziarati *et al.*^124^ reported a host–guest system composed of atomically precise gold nanoclusters incorporated within zinc porphyrin nanorings. Structural characterization revealed strong binding between the pyridine thiol-based Au_25_ nanocluster and the nanoring, which consists of six zinc porphyrin units, thereby enhancing the stability of the gold nanocluster. This system also demonstrated significant activity and selectivity in the photochemical conversion of CO_2_ to CO and in the generation of singlet oxygen.

Accelerating carrier separation is also a widely adopted strategy to mitigate photocorrosion and enhance the stability of PEC CO_2_RR systems. Efficient carrier transfer can substantially mitigate adverse effects, such as structural damage resulting from carrier-induced redox reactions. For example, to address the photocorrosion of Cu_2_O materials, Zhang *et al.*^127^ coated Cu_2_O with a carbon layer and employed a C electron transport layer to accelerate electron transfer, thereby alleviating Cu_2_O corrosion. Jia *et al.*^138^ designed an efficient photocathode system featuring a photonic crystal structure based on Cu_2_O for enhanced light harvesting. The hole transport layer (HTL: FeOOH) mediates and directs interfacial charge transfer from Cu_2_O to FTO, while a polypyrrole (PPy) layer serves as a continuous multi-electron transfer agent for CO_2_ reduction. This integrated photocathode system demonstrated operational stability for 7 h. Liu *et al.*^2^ proposed a protection scheme utilizing a silver catalyst to accelerate the transfer of photogenerated electrons and employing a Z-scheme to extract holes from heterojunctions. The resulting photocathode exhibited a stable photocurrent for CO_2_ reduction under hydrogen equilibrium conditions, achieving a faradaic efficiency of approximately 60% for ethylene over several hours, whereas unprotected Cu_2_O degraded within minutes. It is noteworthy that the reaction system designed by Yao *et al.*^201^ achieved unbiased photoelectrochemical CO_2_ reduction to HCOOH, with a yield of up to 172.9 μmol h^−1^ cm^−2^ over 85 h of long-term testing, outperforming traditional solar-driven systems (Fig. 19b). The oxygen evolution reaction (OER) at the anode was replaced by the sulfur oxidation reaction (SOR, *E* = 0.48 V), which proceeds at a lower reaction rate and effectively suppresses photocorrosion, thereby protecting the anode material.

Currently, research on the development of semiconductor materials resistant to photocorrosion and the optimization of reaction devices remains ongoing, and both areas require further advancement in the future. In the development of new semiconductor materials, Liu *et al.*^82^ employed a bare CuIn_0.3_Ga_0.7_S_2_ photocathode to drive PEC CO_2_RR. Under standard 1 sun illumination, the current density exceeded 2 mA cm^−2^ (at −2 V *vs.* Fc^+^/Fc), and the selectivity for CO production (CO/total products) reached as high as 87%. The system also exhibited long-term stability for syngas production, operating for more than 44 h. Özdemir *et al.*^259^ synthesized a cross-linked boron-dipyrrole methylene (BODIPY) photocatalyst containing a carbazole group. During the course of the reaction, both the turnover number and turnover frequency increased significantly. At a potential of −1.15 V, the faradaic efficiency reached 34.79%. This BODIPY system demonstrated both high activity and long-term stability. Optimizing the reaction device is another effective strategy to enhance system stability. Jung *et al.*^110^ developed a continuous-flow PEC device with a gas-permeable photocathode. By precisely controlling parameters such as the flow rate and pressure of the reaction gas and the flow rate of the electrolyte, both the magnitude of the photogenerated current and the stability of the system could be optimized. Compared to a conventional H-cell using the same photocatalyst material under optimal conditions, the continuous PEC flow reactor achieved a 10-fold increase in the faradaic efficiency for CO, a 30-fold increase in the production rate, and a 16-fold improvement in stability.

## 5 Conclusion and outlook

In recent years, significant progress has been made in the field of PEC CO_2_RR in achieving high selectivity for various products, including CO, HCOOH, CH_3_OH, CH_4_, CH_3_COOH, C_2_H_4_, C_2_H_5_OH and so on. A large number of parameters in the reaction process affect the selectivity of the products. We mainly focus on the effects of the catalyst design, reaction conditions, and the reactor design on product selectivity. The catalyst design is the most widely studied and the most deeply studied aspect at present. It involves aspects such as the type of catalyst, the modification strategy, the composite structure, and the morphology control to achieve the regulation of the active sites on the catalyst surface for the efficient and selective generation of the products. If the design of the catalyst is regarded as the regulation at the micro level, then the design of reaction conditions and the environment represents the regulation at the macro level. The regulation of the catalytic reaction on the catalyst surface can be achieved by changing the external conditions, such as applying voltage, applying external light (including light intensity and light wavelength), reaction temperature, and filling the electrolyte. In addition, the device structure design also affects the absorption of light energy by the photoelectrode, the charge transfer efficiency, and the kinetic mass transfer problem of CO_2_ molecules on the catalyst surface. Although many aspects influencing selectivity need to be considered, there are still patterns to follow. In addition, the thermodynamics and kinetics of the reduction process should also be comprehensively considered during the design to optimize the design of the catalyst, the reaction environment, and the device structure, thereby achieving the regulation of selectivity.

**Fig. 20:**
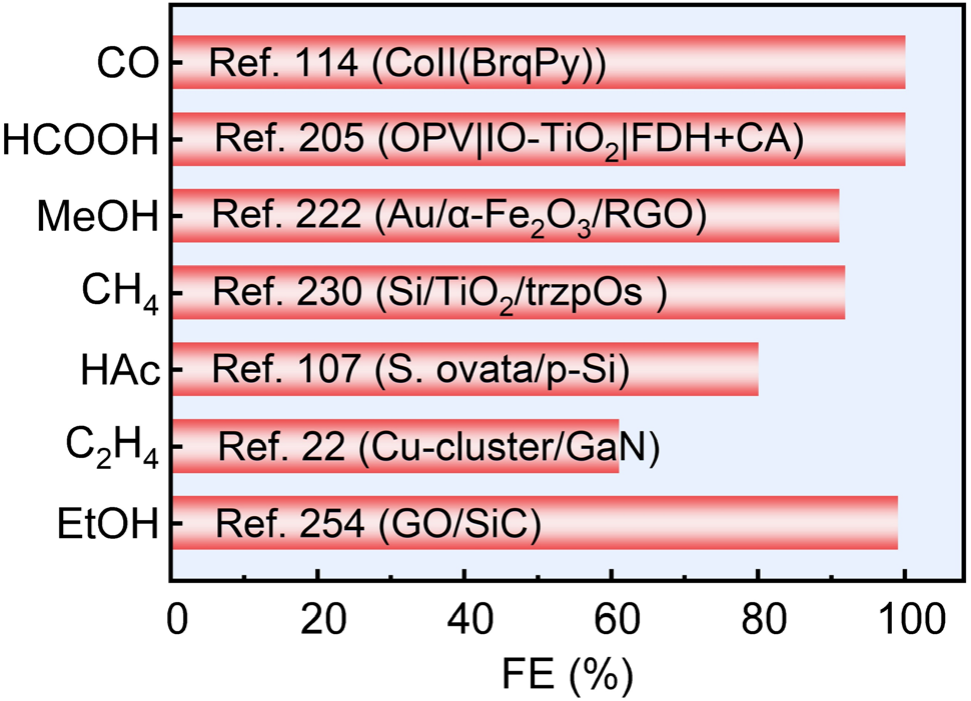
The highest FE of different PEC CO_2_RR products reported in the last five years.

Through a systematic review of the research on PEC CO_2_RR in the past five years, we summarize the optimal FE of the main products (Fig. 20). Among the C_1_ products, the faradaic efficiency of CO and HCOOH is particularly outstanding, with the highest performance reaching nearly 100%. In addition to catalysts with precious metals such as Au and Ag, single-atom catalysts and molecular catalysts can show 100% FE for CO production. The synthesis of HCOOH is dominated by enzyme-based formate dehydrogenase materials and requires precise regulation. In addition, syngas, as a special product combination, has attracted much attention due to its wide application in the industry, and its generation efficiency and adjustable proportion range have also been significantly improved. For the liquid product methanol, important progress has also been made, and the FE of methanol has exceeded 90%. It is worth noting that the high selectivity for methanol in some studies is attributed to catalysts with graphene oxide as the carrier, and it is speculated that there may be a suitable environment for methanol generation on graphene oxide, which still needs further exploration. However, methane is rarely studied, and individual research results show that the FE of methane can reach 90%, and related research still needs to be further advanced. Among the C_2+_ products, research has mainly focused on the reduction of acetic acid, ethylene, and ethanol, and Cu-based catalysts are the most commonly used, but due to the formation of a large number of by-products, it is difficult to improve the selectivity. The generation of acetic acid involves a diversified strategy, in which a combination of Cu_2_O and TiO_2_ as well as cathodic microbial electrosynthesis seem to be good options. In the current research, Cu-based catalysts are the main choice for ethylene production, and new catalyst types still need to be further developed. Ethanol has shown the most significant research progress among the C_2+_ products, involving various types of catalysts, such as TiO_2_, Cu-based materials, and non-Cu-based materials (BiFeO_3_ and SiC). Among them, the application of GO/SiC materials enables the selectivity for ethanol reach almost 100%. For other C_2+_ products, although they can be produced using various types of catalysts, their generation is difficult and the selectivity efficiency is low. In general, the high selectivity and generation efficiency of C_1_ products are still important goals in the current research on PEC CO_2_RR, while C_2+_ products represent the potential for future technological development. Finally, it is important to conduct in-depth investigations into the limited reaction stability of PEC systems, which severely hinders the achievement of high product selectivity. One possible cause is the material instability induced by light irradiation; however, this mechanism requires further detailed exploration. The construction of catalyst coating layers and the enhancement of rapid charge carrier separation have already demonstrated significant improvements in reaction stability, with reaction times extended to approximately 80 h.

The PEC CO_2_RR technology provides an innovative approach for achieving carbon neutrality and the synthesis of high value-added chemicals. However, research in this field is still in its infancy. The precise control of product selectivity still faces multiple challenges at the material, mechanism, and system levels, and a significant amount of work is still required to improve the conversion efficiency and product selectivity.

### 5.1 Precise regulation of catalysts

In the PEC CO_2_RR, the selectivity and efficiency of the catalyst are directly affected by the properties and structure of the catalyst itself. Although metal catalysts such as copper-based catalysts have made significant progress in generating C_2_ products (such as ethylene and ethanol), their selectivity and stability still need to be further improved. Therefore, precisely regulating the microstructure, surface active sites, and electronic properties of the existing catalyst is crucial for optimizing the catalytic performance. By adjusting the size, morphology, and surface functional groups of the metal particles, as well as their coordination with other elements, the product selectivity and FE can be significantly improved. In addition, rationally designing the electronic coupling effect between metals and optimizing the adsorption characteristics of the catalyst surface are also the keys to achieving high selectivity.

### 5.2 Improvement of reaction stability

Reaction stability is one of the core challenges in achieving efficient and sustainable conversion. Currently, photoelectrocatalytic systems commonly suffer from instability issues caused by photocorrosion, interfacial mismatches, and fluctuations in reaction conditions, which severely affect long-term operation and product selectivity. Future research directions for improving reaction stability include the development of novel catalyst materials with enhanced resistance to photocorrosion, the construction of efficient interface engineering strategies, and the optimization of light absorption, charge carrier separation, and transport pathways through advanced device design. For example, employing multilayer heterojunction structures, introducing protective layers or self-healing mechanisms, and integrating advanced device architectures such as microfluidic systems are expected to significantly enhance the overall stability and efficiency of PEC CO_2_RR, thereby advancing their progress toward practical application.

### 5.3 Device design and reaction environment design

The regulation of device design and the reaction environment has an equally important impact on the efficiency and selectivity of the PEC CO_2_RR reaction. By optimizing the structure of the photoelectrocatalytic device, such as the shape, thickness and porosity of the electrodes, as well as the geometric structure of the device, the contact area between reactants can be expanded and the photocurrent density can be increased, thereby promoting the reaction. At the same time, the regulation of the reaction environment, such as the pH of the reaction solution, temperature, and the selection of the electrolyte, can also significantly affect the reaction path and product distribution. By precisely adjusting these parameters, it is possible to achieve the highly selective generation of different products. In the future, the development of reactors with high stability and operability will have a positive impact on the large-scale application of the PEC CO_2_RR technology. In theoretical calculations, the corresponding microenvironment can be included in simulation, but its influence is usually ignored.

### 5.4 Deep analysis of the multi-scale reaction mechanism

A thorough understanding of the mechanism of the PEC CO_2_RR reaction is the basis for improving the performance of the catalyst, optimizing the reaction path, and enhancing the selectivity of the products. Currently, although some studies have revealed details of some reaction mechanisms, the formation of intermediates in the reaction process, the kinetics of electron–proton transfer, and the active sites on the catalyst surface are still not completely clear. In the future, in-depth studies on each stage of the reaction process should be strengthened, especially by using high-resolution characterization techniques (such as *in situ* spectroscopy, electron microscopy technology, *etc.*) to track the evolution process of intermediates, thereby providing a scientific basis for the design and optimization of the catalyst. In addition, with the development of computational chemistry, the use of computational simulations to further reveal the reaction path and catalytic mechanism can accelerate catalyst design and reaction optimization.

This review mainly expounds the factors influencing the selectivity of the PEC CO_2_RR products and summarizes the research progress on commonly studied products, in order to provide insights and ideas for future efforts to improve product selectivity. It is expected that in the near future, the practical application of photoelectrocatalytic CO_2_ to achieve C_1_ products at the industrial scale and significant improvements in the selectivity and efficiency of C_2+_ product generation will be realized.

## Author contributions

All of the authors contributed to the manuscript preparation. G. Zhou conceptualized the work, performed the investigations and wrote the manuscript. Z. Wang and J. Gong provided resources and edited the manuscript. S. Shen and W. Zhong were involved in funding acquisition and project administration.

## Conflicts of interest

There are no conflicts to declare.

## Data Availability

Data availability is not applicable to this article as no new data were created or analyzed in this study.
